# A Design Principle of Group-level Decision Making in Cell Populations

**DOI:** 10.1371/journal.pcbi.1003110

**Published:** 2013-06-27

**Authors:** Koichi Fujimoto, Satoshi Sawai

**Affiliations:** 1 Graduate School of Science, Osaka University, Toyonaka, Osaka, Japan; 2 Graduate School of Arts and Sciences and Research Center for Complex Systems Biology, University of Tokyo, Meguro-ku, Tokyo, Japan; 3 PRESTO, Japan Science and Technology Agency (JST), Kawaguchi, Saitama, Japan; University of Notre Dame, United States of America

## Abstract

Populations of cells often switch states as a group to cope with environmental changes such as nutrient availability and cell density. Although the gene circuits that underlie the switches are well understood at the level of single cells, the ways in which such circuits work in concert among many cells to support group-level switches are not fully explored. Experimental studies of microbial quorum sensing show that group-level changes in cellular states occur in either a graded or an all-or-none fashion. Here, we show through numerical simulations and mathematical analysis that these behaviors generally originate from two distinct forms of bistability. The choice of bistability is uniquely determined by a dimensionless parameter that compares the synthesis and the transport of the inducing molecules. The role of the parameter is universal, such that it not only applies to the autoinducing circuits typically found in bacteria but also to the more complex gene circuits involved in transmembrane receptor signaling. Furthermore, in gene circuits with negative feedback, the same dimensionless parameter determines the coherence of group-level transitions from quiescence to a rhythmic state. The set of biochemical parameters in bacterial quorum-sensing circuits appear to be tuned so that the cells can use either type of transition. The design principle identified here serves as the basis for the analysis and control of cellular collective decision making.

## Author Summary

Although the genetic circuits underlying state switching at the single-cell level are well understood, how such circuits work in concert among many cells to support the population-level switching of cellular behaviors is not fully explored. Experiments using microbial signaling systems show that group-level changes in cellular state occur in either a graded or an all-or-none fashion. We show that the type of group-level decision making used by populations is uniquely determined by a single dimensionless parameter that compares the quorum-signaling molecules accumulated within the cells with those secreted by the population. Bacterial quorum-sensing circuits appear to be tuned so that the cells can convert between the two types of decision-making in response to slight biochemical variations. Furthermore, the role of the parameter is universal such that it not only applies to the autoinducing circuits typically found in bacteria but also to the more complex gene circuits involved in transmembrane receptor signaling and negative feedback. The design principle that we describe thus serves as the basis for the analysis and control of collective cellular decision making in general.

## Introduction

**Figure 1:**
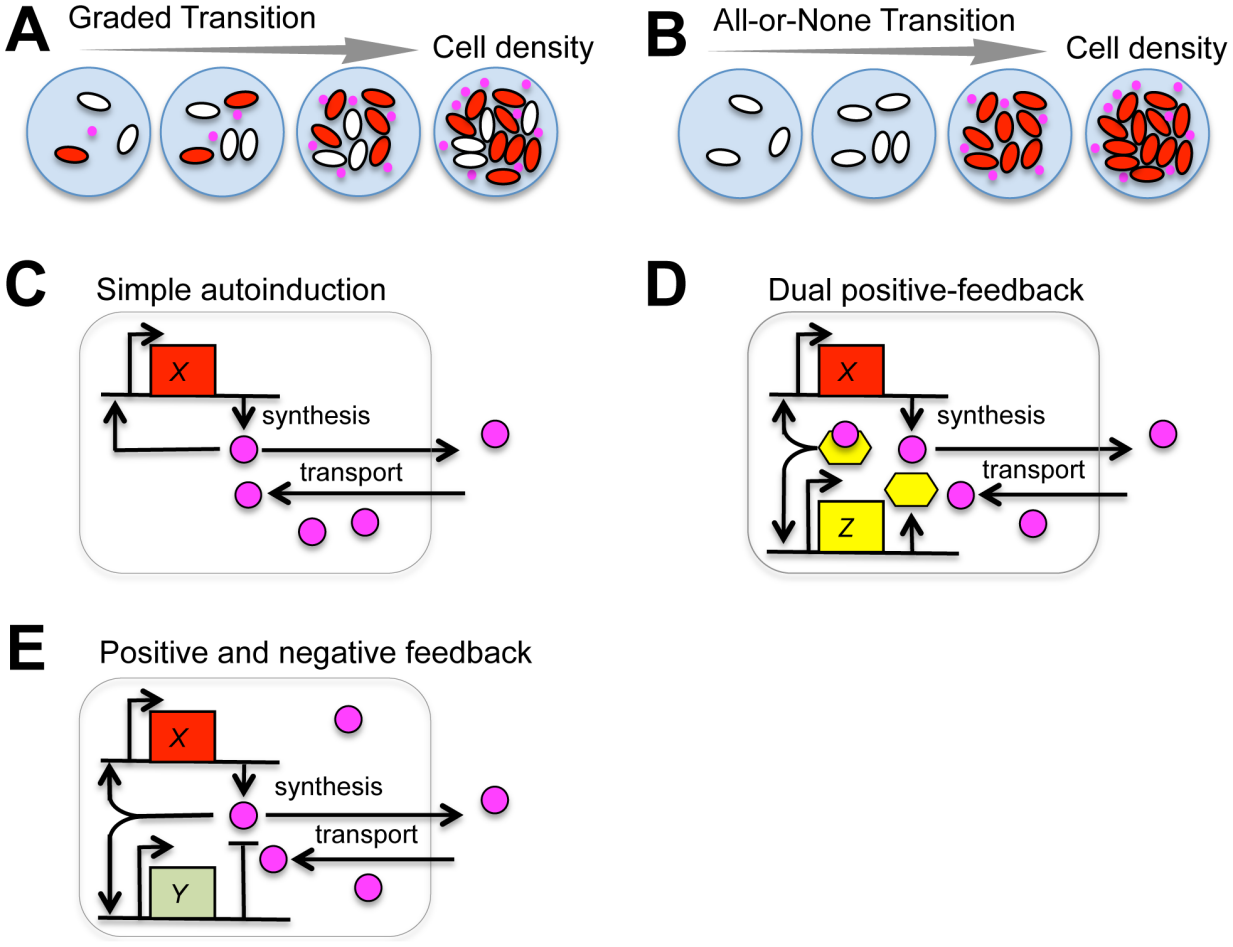
Gene circuit design for group-level transitions. (**A–B**) Schematics of two types of group transition in response to changes in cell density. (**A**) A graded transition is seen when the fraction of cells in the ON state (red) gradually increases with cell density. (**B**) An all-or-none transition appears when the state change occurs simultaneously across the population. (**C–E**) Schematics of an autoinducing gene circuit model (**C**; Eq. 1), dual-positive feedback regulations (**D**; Eq. 3), and positive-and-negative feedback regulations (**E**; Eq. 4) in operation.

Cells often switch their state autonomously, either individually or as a group [1]–[3]. The cell-autonomous switch is exemplified by the classical molecular switch in Bacteriophage Lambda: the *cI* and *cro* genes mutually repress one another and thus operate as a genetic toggle switch between the lytic and lysogenic cycles [4]. A common network topology [5], [6] that realizes either the positive autoregulation of inducing signals [7] or the mutual repression of inhibitory signals [8] is generally responsible for the all-or-none responses of individual cells. Bistable behavior at the single-cell level does not, however, necessarily translate into an all-or-none response at the group-level. Because of stochasticity in gene expression [9] and variability among cells in their sensitivity to environmental change [10], [11], the switch is graded at the population level [12], [13]; i.e., cells in the ON state coexist with cells in the OFF state [1]–[3], [7], [8] (Fig. 1A). There are many cases; e.g., bacterial quorum sensing (QS) [14], [15]; however, where the transition is abrupt and occurs in an all-or-none fashion even at the group level (Fig. 1B). In QS, cells secrete inducing molecules that signal neighboring cells to synthesize and secrete more of the same inducing molecules; thus, global positive feedback is realized (Fig. 1C). The autoinducer Acyl-homoserine lactone (AHL) is an inducing molecule [16]–[19] in populations of the luminescent symbiotic bacterium *Vibrio fischeri* and of other bacteria species [14], [15]. In animal development, a collective state change within a differentiating tissue is referred to as ‘community effect’ [20], [21]. Generally, a group-level transition between cellular states manifests itself via a combination of cell-autonomous and group-level mechanisms; these two modes of transition, however, have not been clearly distinguished from one another thus far.

In QS, both the graded and the all-or-none types of transitions are observed at the group level [22]–[27]. In a graded transition, cells in the ON and OFF states coexist within a population; thus, the state of the cells follows a bimodal distribution. Such a behavior is observed in populations of the free-living bacterium *V. harveyi*, the virulent pathogen *Salmonella typhimurium*, and *Listeria monocytogenes*; in these populations, the percentage of cells in the ON state increases gradually as cell density increases or other environmental factors change [25], [26], [28]. Similar behavior occurs in engineered *E. coli* that harbors synthetic *luxI* and *luxR* genes encoding AHL synthetase and a transcriptional activator [22], [23]. When the regulation of the *lux* genes is synthetically rewired, however, the entire population synchronously switches its pattern of gene expression when cell density reaches a certain threshold [24]. Such sharp population-level transitions underlie important biological phenomena such as bioluminescence and virulence in a wide range of species from *V. fischeri* to the opportunistic pathogen *Pseudomonas aeruginosa* (see Fig. 1 in [29]; Fig. 2 in [30]; Fig. 2 in [31]). Interestingly, when *V. fischeri* cells are isolated in a chamber while continuously being cleared of AHL by dilution, their response to exogenously applied AHL is heterogeneous [10]. Thus when making the all-or-none switch as a population, cell-cell variability must be somehow suppressed by cell-cell communication. Because most existing mathematical models of QS are formulated either entirely at the single-cell [10], [32], [33] or the population level [16]–[19], the relationship between the graded and the all-or-none transitions and the underlying bistability of cellular states have not received a full theoretical treatment.

**Figure 2:**
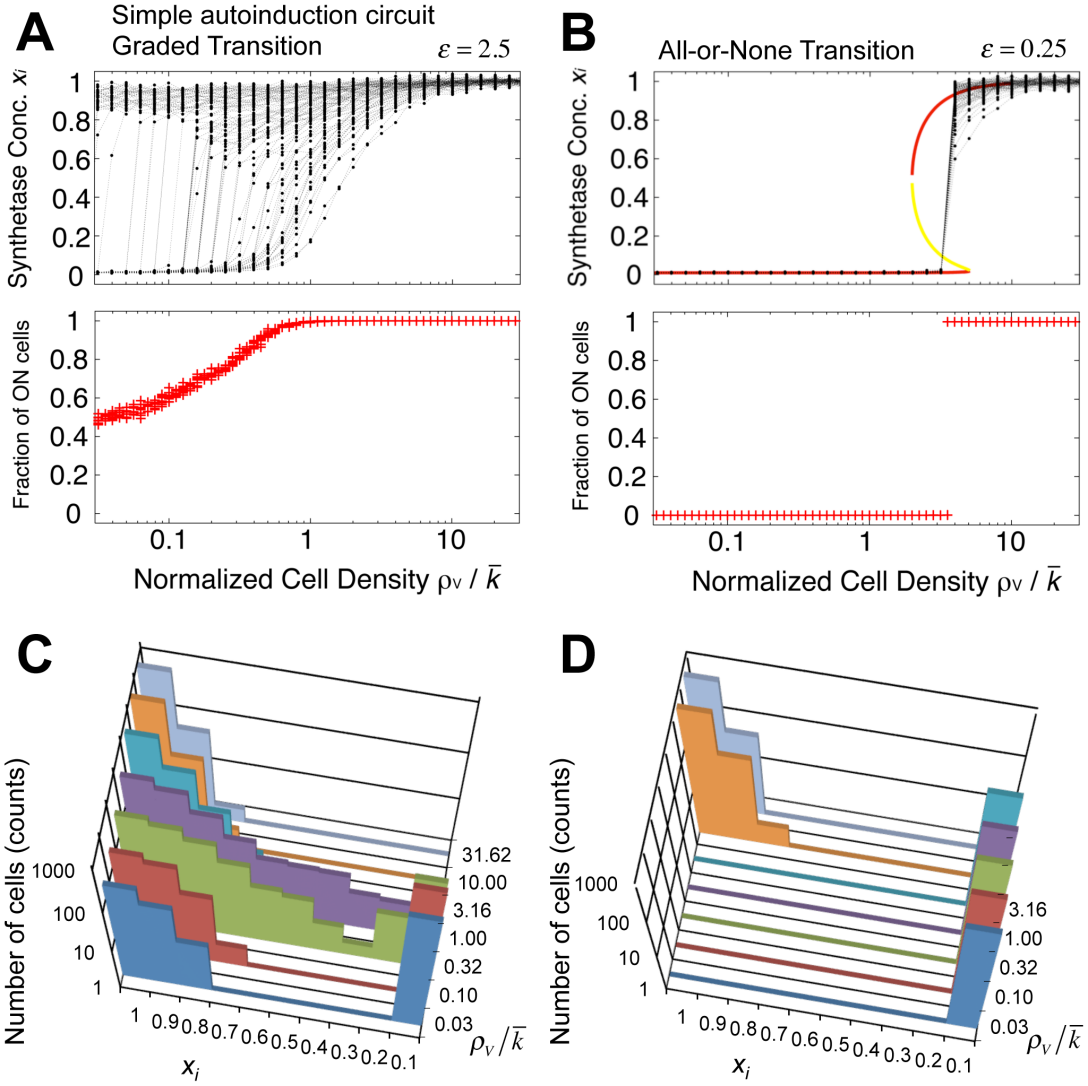
Graded and all-or-none transitions in a simple autoinduction circuit. (**A–B**) In the population of simple autoinduction circuits (Fig. 1C; Eq. 1), the time average of the synthetase concentration *x_i_* within individual cells at the steady state (upper panel) and the fraction of ON cells (*x_i_*>0.5; lower panel) are plotted as a function of normalized cell density *ρ_V_*; *ε* = 2.5 (**A**) and 0.25 (**B**); *λ* = 100 (**A–B**). Data points in the lower panel were calculated from multiple randomized sets of threshold *k_i_* with identical standard deviations. An analytically derived steady state for the population mean 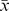 (dashed line; Eq. S2-1 in Text S1) indicates group-level bistability for 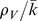 = 2∼5 (**B**). (**C–D**) Change in the distribution of *x_i_* for various cell densities: a bimodal distribution at high *ε* (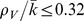; **C**) and a unimodal distribution at low *ε* (**D**). *ε* = 2.5 (**C**) and 0.25 (**D**). *λ* = 100 (**C–D**).

To clarify the mechanisms of group-level transitions, we numerically and analytically studied general classes of mathematical models that describe QS across two levels of organization; i.e. single-cell and cellular-ensemble. We show that graded transitions occur when the intracellular positive feedback, mediated by the synthesis and accumulation of autoinducer molecules within the cells, alone can support bistability. Conversely, we show that all-or-none transitions occur when the secreted signal within the population serves predominantly to realize bistability at the group-level. We identify a unique dimensionless parameter, representing the respective relative contributions to the regulatory feedback of the intracellular and extracellular autoinducer molecules, that determine the type of transition and the underlying bistability. We find that in many bacterial species, this parameter is near the optimal value for allowing the bacteria to select between the two transition types depending on environmental conditions. We explored this common design principle in a basic circuit with negative feedback. The types of cells harboring such circuits range from particle-based chemical reactions [34] to engineered *E. coli* [35], [36], yeasts [37], and the social amoeba *Dictyostelium discoideum* [38]. These systems are known to exhibit density-dependent transitions from quiescence to an oscillatory state [39]. We show that the same unique parameter determines whether the transition from quiescence to oscillation occurs gradually or synchronously.

## Models

### A simple autoinduction circuit

To analyze group-level transitions at both the single-cell level and the group level, we studied three basic circuit topologies (Fig. 1C–E). For simple autoinduction (Fig. 1C) and a dual positive-feedback circuit (Fig. 1D), we employed a previously described quantitative model [27]. First, for the simple autoinduction circuit (Fig. 1C), when the extracellular and intracellular synthesis and degradation of the autoinducer are rapid compared with changes in the synthase concentration, the autoinducer concentration can be approximated by the steady-state. Accordingly, the equations can be simplified to 
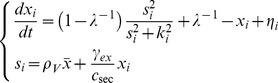
(1)(See Supporting Information Text S1 1.1 for a detailed derivation), where *x_i_*, 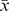, *s_i_*, and *k_i_* are the normalized intracellular concentration of the synthetase, the population mean 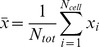 concentration of the synthetase, the normalized intracellular concentration of the autoinducer and the normalized the threshold concentration for the induction (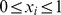; See Table 1 for representative examples in bacterial QS). *ρ_V_* represents the volume fraction *ρ_V_* = *N_cell_V_cell_*/*V_tot_*, where *N_cell_*, *V_cell_*, and *V_tot_* denote the number of cells, volume of a single cell and the total volume including both intracellular and extracellular space, respectively. Based on experimental data (Table S1), Hill coefficient is set to 2 which supports bistability. When the Hill coefficient is equal to 1, bistability does not exist (Text S1 2.1). The amplification factor *λ* determines the ratio of basal to maximal rate of QS molecule synthesis (Eq. S1-4 in Text S1) when *s_i_* is above a certain concentration *k_i_* (Eq. S1-3 in Text S1) [14], [15], [18]. The dimensionless parameter *ε* is given by
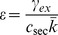
(2)(See Eq. S1-13 in Text S1 for derivation), where 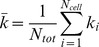, and *γ_ex_* and *c_sec_* denote the degradation and secretion rates, respectively. *ε* essentially compares synthesized autoinducer concentration with the threshold (Eq. S1-14 in Text S1)_._ In the present study, intracellular degradation of the autoinducer was not taken into account. This approximation holds as long as the intracellular degradation rate is much smaller than *c_sec_*. The overall results are not affected by this assumption, because *ε* is independent of the ratio between *γ_in_* and *γ_ex_* (see Text S1 1.5 for a detailed calculation). The advantage of this simplification is that, besides the volume fraction of cell density 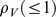, the model is left with only two parameters, *ε* and *λ*, both of which can be experimentally measured (Tables S1 and S6) and manipulated [27], [40] (Table S2).

**Table 1:** Examples of the simple autoinduction (Eq. 1) and the dual positive-feedback (Eq. 3) circuits in quorum sensing systems.

System	*s* #1	*x* #2	*z* #3	Ref.
Bioluminescence in *V. fischeri*	3-oxo-C6-HSL*	LuxI	LuxR	[14], [89]
Biofilm formation in *P. aeruginosa*	C4-HSL	RhlI	RhlR	[90]
Virulence and biofilm formation in *P. aeruginosa*	3-oxo-C12-HSL	LasI	LasR	[91]
Virulence in *E. carotovora*	3-oxo-C6-HSL	CarI	CarR	[92]

#1 Autoinducer concentration.

#2 Autoinducer synthetase concentration.

#3 Transcriptional activator concentration.

* HomoSerine Lactone. 3-oxo-C6-HSL is a major AHL produced by *V. fischeri*.

In this model and those described below, the value of *k_i_* is randomly distributed [11] around the mean 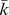 to account for cell-cell variability in the response to exogenously applied autoinducer [10] (Fig. S1A–B). In addition, to reflect heterogeneous gene expression within the population [9], [25], the model assumes intrinsic stochasticity in the rate of synthetase production, which follows Gaussian white noise *η_i_* [41], [42]. The molecules are passively transported into and out of the cells at the rate *c_sec_*, and degraded extracellularly at the rate *γ_ex_* (Eqs. S1-1 and S1-2 in Text S1) [43]. Here, we assumed that the autoinducer molecules diffuse rapidly so that they are well mixed in the extracellular space. The extracellular concentration of the autoinducer is proportional to the cell density *ρ_V_* (Eq. S1-1 in Text S1) [17] and can be considered almost uniform in space for systems smaller than 1 mm (Text S1 1.6).

### A dual positive-feedback circuit

The second model that we shall study here describes a circuit with an additional intracellular positive feedback (Fig. 1D): 
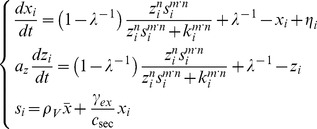
(3)where *z_i_* is the normalized intracellular concentration of a transcriptional activator (Fig. 1D; Table 1). In addition to the three parameters already described; i.e. *ρ_V_*, *λ*, and *ε* (Eq. 2; Eq. S1-18 in Text S1); *m* and *n* denote the Hill coefficients of binding between the signal molecule and the transcriptional activator and between the transcriptional activator and its target promoter, respectively (see Text S1 1.2 for a derivation). Such dual positive-feedback loops are common in bacterial QS [15], [27] (Table 1). In the *lux* operon of *V. fischeri*, the autoinducer AHL binds to the transcriptional regulator LuxR, forming a complex that binds to a promoter of both the *luxR* and the *luxI* genes, which encode the LuxR and the synthetase, respectively [14], [15], [18], [27]. Because LuxR cannot be exported outside the cell, the LuxR feedback mechanism only works intracellularly; the autoinduction mediated by the LuxI feedback, however, works both intracellularly and extracellularly.

### A positive-and-negative-feedback circuit

Our third model describes a basic circuit with positive and negative feedback loops (Fig. 1E). The model is given by
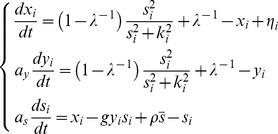
(4)where *y_i_* denotes the normalized concentration of an inhibitor, and *ρ* = *ρ_V_*/(*ρ_V_*+*γ_ex_*/*c_sec_*) (see Text S1 1.3 for a derivation and Table 2 for representative examples). Here, the mean threshold 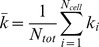 corresponds to the inverse of the order parameter *ε* (Eq. 2; Eq. S1-24 in Text S1). This type of circuit has been previously modeled and implemented in a synthetic circuit where AHL activates the production of its own synthetase, LuxI (X), and of lactonase AiiA (Y); and AiiA degrades AHL [35].

**Table 2:** Examples of the positive-and-negative-feedback circuit (Eq. 4) in dynamical quorum sensing systems.

System	*S*	*X*	*y* $	Ref.
Engineered *E. coli*	3-oxo-C6-HSL	LuxI	AiiA	[35]
cAMP signaling in *D. discoideum*	cAMP	Adenylyl cyclase	cAMP receptor	[62], [93]
Glycolysis in *S. cerevisiae*	Acetaldehyde	N/A	ATP	[75], [94]

$ Negative regulator of autoinducer synthetase *x*.

### Numerical experiments

**Figure 3:**
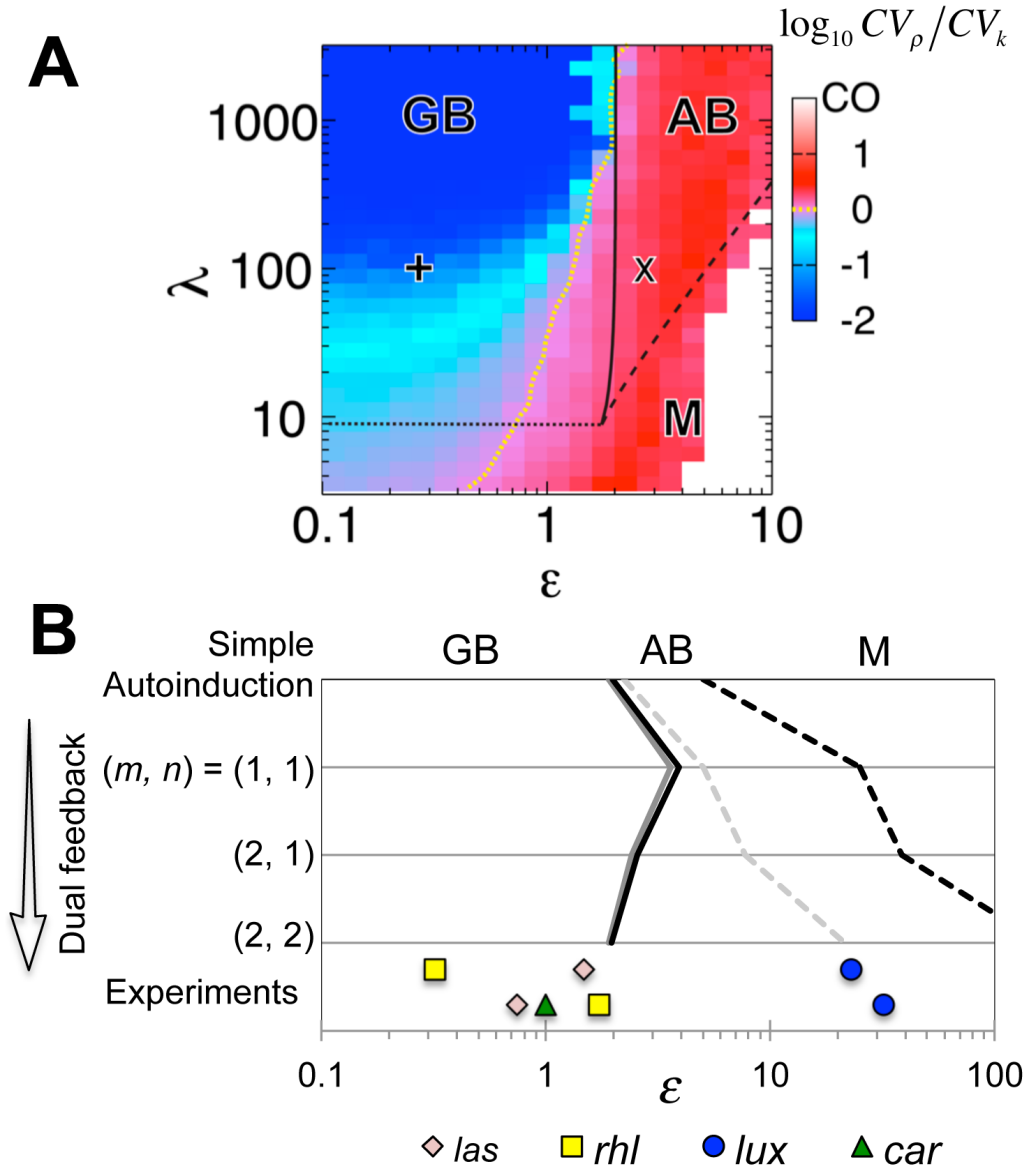
Conditions for group-level transitions in quorum sensing. (**A**) A phase diagram showing the bistable regions in parameter space (*ε*, *λ*) of the simple autoinduction circuit (Eq, 1). The black lines indicate phase boundaries between GB (Group-level Bistability), AB (Autonomous-Bistability), and M (Mono-stability) (Eqs. S2-14 and S2-6 in Text S1). CO (Constitutively ON) means the cells are always in the ON state; i.e., *x_i_*∼1, regardless of cell density in the case of M (white region). Log_10_ *CV_ρ_*/*CV_k_*<0 (blue and cyan region) and log_10_ *CV_ρ_*/*CV_k_*>0 (red and pink region) indicate regions where cell-cell heterogeneity in the threshold value is reduced or not reduced, respectively. The yellow line indicates log_10_ *CV_ρ_*/*CV_k_* = 0, determined numerically with spline interpolation. The × and + correspond to Figs. 2A and 2B, respectively. (**B**) A phase diagram for the simple autoinduction circuit (Eq. 1; Fig. 1C) and the dual positive-feedback circuit (Eq. 3; Fig. 1D). The *m* and *n* are cooperativity coefficients (Eq. 3). As in (**A**), solid and dashed lines denote the phase boundaries between PB/CB and CB/M, respectively (Eqs. S2-15 and S2-23 in Text S1). *λ* = 20 (grey) and 100 (black). The bottom row shows the parameter *ε* for the bacterial QS operons, *las*, *rhl*, *lux*, and *car* estimated from the literature (Table S6). For the *las*, *rhl*, and *lux* systems, estimates for two independent data sets are plotted.

**Figure 4:**
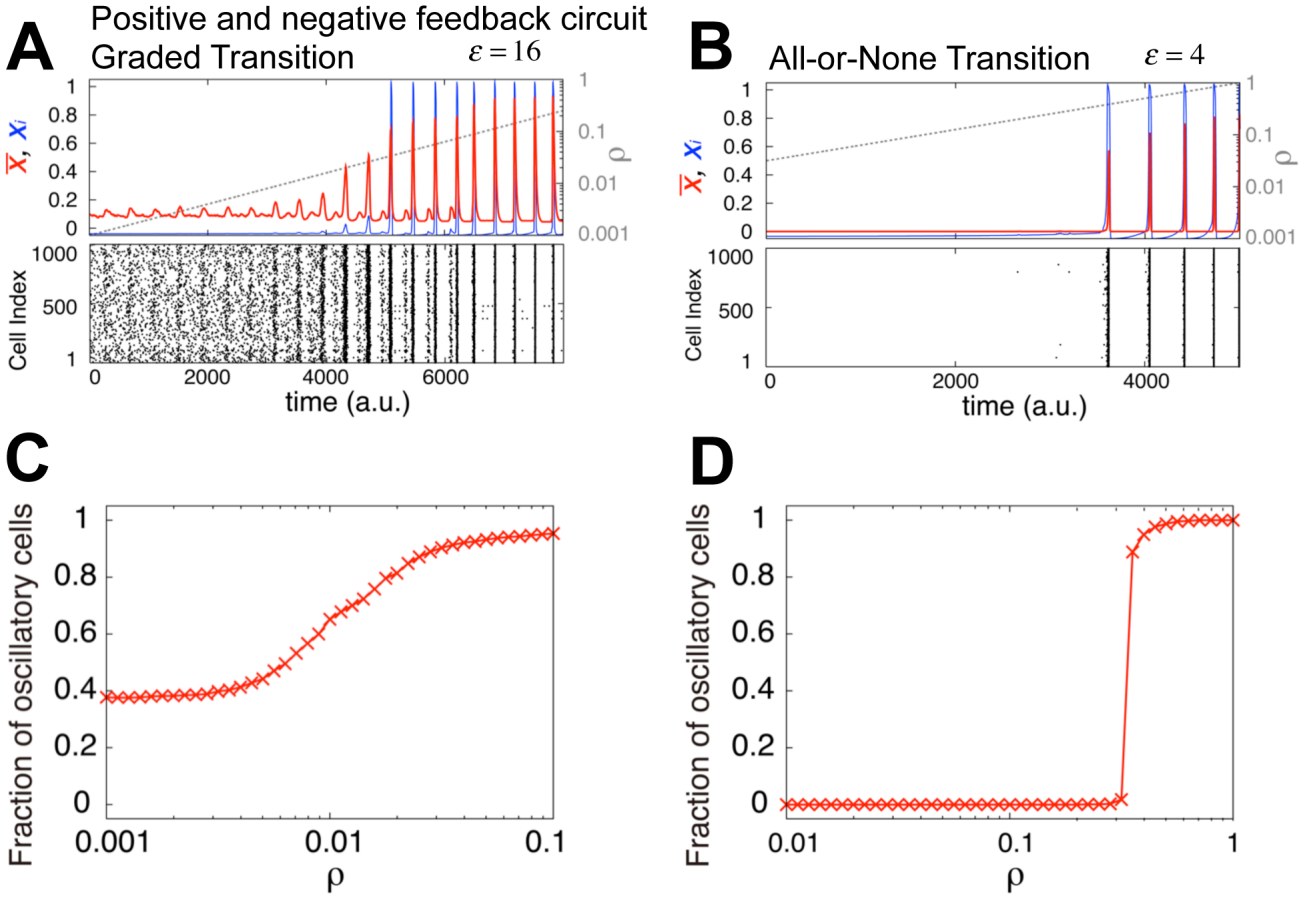
Graded and all-or-none transitions to collective oscillations. (A–B) Representative time courses of oscillatory transitions within populations of positive-and-negative feedback circuits (Fig. 1E; Eq. 4) for high *ε* (*ε* = 16; A,C) and low *ε* (*ε* = 4; B,D). *λ* = 10^3^, *g* = 30, and |*η_i_*| = 0. Upper panels: time course of the population mean (red line) and the synthetase concentration of a representative cell (blue line) during an exponential increase in cell density *ρ* (grey line). Lower panels: pulsatile responses of individual cells. Y-axis indicates the cell index. (C–D) Percentage of oscillatory cells plotted as a function of cell density.

Numerical integration of Eqs. 1, 3, and 4 was performed using the fourth-order Runge-Kutta algorithm. All programs were written using the C programming language. The cell-density dependence was examined by decreasing the volume of extracellular space exponentially while keeping the number of cells at 1,000 (Fig. 2). Accordingly, cell density *ρ_V_* and extracellular autoinducer concentration increase exponentially thereby effectively implementing the growth phase of a population. The rate of volume decrease is set to 1/40 of the degradation rate of the synthetase (Eqs. 1 and S1-1 in Text S1) for the phase diagrams (Figs. 3A and S3.). As long as this ratio is small, the present results do not depend on the exact rate of volume reduction, as will be described in the Results section. Except where we study the phase diagrams and the time course of the negative-and-positive-feedback circuit (Fig. 4A–B), plots were obtained at the steady state. The initial concentrations of *x_i_*, *y_i_*, *z_i_*, and *s_i_* were set randomly between 0 and 0.01.

To examine cell density dependence, we first defined the threshold cell density for each cell. For the autoinduction and dual positive-feedback circuit (Eqs. 1 and 3), the threshold density for the *i*-th cell *ρ_i_* was determined by the volume fraction of density *ρ_V_* at which the normalized concentration *x_i_* (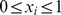) took the half maximum *x_i_* = 0.5. In case of the positive-and-negative-feedback circuit (Eq. 4), the threshold density *ρ_i_* was defined by the density at which the temporal evolution of *x_i_* switched from quiescence to oscillations. As a measure of cell-cell variability at the onset of the transition, the standard deviation of *ρ_i_* normalized by its population mean 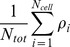 was denoted *CV_ρ_* (coefficient of variation of *ρ_i_*). Likewise, *CV_k_* was defined by the standard deviation of *k_i_* normalized by the mean 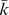. *k_i_* follows lognormal distribution with *CV_k_* = 0.5.

Following the formulation of a chemical Langevin equation [44], the variance in noise |*η_i_*|^2^ at the steady states of Eqs. 1, 3, and 4 is given by |*η_i_*|^2^ = 2 *x_i_*/*γ_X_ N_ON_* (Eq. S1-11 in Text S1), where *N_ON_* and *γ_X_* are the number of synthetase molecules within a cell that is in the ON state and the degradation rate of the synthetase, respectively (Eqs. S1-12 and S1-3 in Text S1). We set *γ_X_N_on_* to 140. *γ_X_* = 1 corresponds to *N_on_* = 140 molecules, or a 60 nM synthetase concentration with cell volume of approximately 3.6×10^−15^ L [45].

## Results/Discussion

### Graded and all-or-none transitions in cell populations and the underlying bistability

First, we will numerically study the cell-state transitions that depend on the cell density *ρ_V_* in the simple autoinduction circuit (Eq. 1). The key parameter that distinguishes the transitions at the group level is the concentration of intracellular autoinducing signal (*s_i_* = *γ_ex_ x_i_/c_sec_*+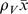 in Eqs. 1 and S1-6 in Text S1): *γ_ex_ x_i_/c_sec_* represents the intracellular feedback on signal synthesis caused by the cell itself, and 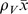 measures the strength of the feedback mediated by the secreted signal. When the threshold concentration *k*_i_ and the secretion rate of autoinducer molecules *c_sec_* are low, the secretion-mediated feedback is relatively weak, so the induction depends mainly on the feedback from intracellular synthesis. In this case, the cells turn themselves on individually, provided that the concentration of the autoinducer accumulated inside the cell (*s_i_*∼*γ_ex_ x_i_/c_sec_*) is higher than the threshold *k*_i_. The switch gives rise to two stable states that are each self-enforcing. The OFF state at *x_i_*∼*λ* ^−1^ keeps cells in a state of low autoinducer synthesis (upper panel of Fig. 2A). Likewise, once the cells are in the ON state (*x_i_*∼1), the high rate of autoinducer synthesis will keep them in the ON state (upper panel of Fig. 2A). Because the two stable states do not require secreted signal from other cells (see coexistence of ON and OFF cells in Fig. S1A), we shall refer to this as “cell-autonomous bistability”. The word “group-level” is a relative term; and more accurately, it is the volume fraction of cell density, rather than the absolute number of cells, that essentially controls the level of extracellular autoinducer molecules. Indeed, a single cell confined to a small chamber has been shown to turn on its QS genes [46], [47].

The signature of group-level transitions that are driven by the single-cell-level switch is the coexistence of cells in the ON state and cells in the OFF state within the population (low density in the upper panel of Fig. 2A). Here, the percentage of cells in the ON state gradually increases as a function of the cell density (lower panel of Fig. 2A). This is clearly demonstrated by the bimodal distribution of cellular states *x_i_* when the cell density *ρ_V_* is in the intermediate range (Fig. 2C). Individual cells switch in an all-or-none manner, however, at different densities (upper panel of Fig. 2A; see also Fig. S1C for the coexistence in the nullclines). Thus, at the population level the transition becomes graded. Recent experimental observations of bimodal distributions of cell states and graded group-level transitions [22], [23], [25], [26] suggest that, in many bacterial QS systems, the contribution of cell-cell communication is rather weak and the switch is caused by cell-autonomous bistability.

In contrast, at high *k*_i_ and *c_sec_* the amount of inducing molecules secreted extracellularly (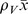 in Eq. 1) becomes profound. When the secretion-mediated feedback 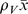 becomes negligible in the isolated condition, because of the continuous clearance of extracellular signals via degradation or dilution [10], [38], the cells cannot exhibit bistability (Fig. S1B). Fig. 2B shows that above a cell-density threshold, all cells change their state simultaneously. Although the ON state (*x_i_*∼1) and the OFF state (*x_i_*∼*λ* ^−1^) are identical to the states that appear in the case of cell-autonomous bistability, the entire population must now either be ON or OFF (Fig. 2B and 2D). The two states cannot coexist within the population (see also Fig. S1D for a nullcline analysis). This group-level all-or-none transition is mediated by the feedback from the secreted autoinducer molecules in the extracellular space (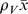 in Eq. 1). Because the concentration of the synthesized signal within the ON-state cells (*γ_ex_/c_sec_*) is below the threshold *k*_i_, the ON state cannot be self-sustaining unless a sufficient amount of signaling molecules are synthesized and secreted by other cells. To distinguish this form of bistability from the cell-autonomous bistability described above, we shall hereafter refer to it as “group-level bistability”. Frequent experimental observations of such all-or-none transitions in many bacterial systems [14], [15], [30], [31] suggest that the occurrences of group-level bistability are widespread.

Graded transitions and all-or-none transitions both involve a combined action by the cells. Although cell-autonomous bistability underlies the graded transition, the switch is nonetheless density dependent, and there is a cooperative effect within the group of cells. Whether a cell can switch its state depends on its position in the state space relative to the basin of attraction (Fig. S1C, low density). By plotting the synthetase production rate *dx_i_/dt* as a function of the synthetase concentration *x_i_*, we see that the range of initial concentrations that converge to the ON state expands as the density is increased (Fig. S1E). Because the concentration of the autoinducer in the OFF state (*s_i_*∼*γ_ex_λ^−1^/c_sec_*+*ρ_V_ λ^−1^*; *x_i_∼λ^−1^*) is close to the threshold *k_i_*, the probability that a cell switches from the OFF state to the ON state increases with the density *ρ_V_*. On the other hand, cell-cell communication is absolutely essential for group-level bistability. At intermediate cell densities, the concentration of secreted autoinducer (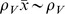) exceeds the average threshold 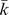 (i.e., 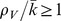, Fig. 2B), so cells that have not yet switched are forced to do so (Fig. S1F). Likewise, when the synthesized concentration is insufficient to sustain the cells in the ON state, the whole population converges to the OFF state at the steady state. Thus, although there is a difference of degree, both types of bistability depend on the interactions among the cells within the population.

### Design principle of autonomous/group-level bistability

To help identify the design principle underlying graded and all-or-none transitions, we analytically derived a unique dimensionless parameter *ε* (Eq. 2) that determines the nature of the bistability (Text S1 2.1 and 2.2). Essentially, *ε* compares the magnitude of the inducing signal that is synthesized intracellularly (*γ_ex_/c_sec_*) with the response threshold 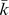 (Eq. S1-13 in Text S1). A solid line in Fig. 3A indicates the analytically obtained boundary (*ε*∼2) in the parameter space (*ε*, *λ*) that separates autonomous bistability from group-level bistability (Text S1 2.2). The border matches well with the results of numerical simulations (Fig. S2A). For *ε*>2, even isolated cells can take two stable fixed points (Eq. S2-15 in Text S1), which indicates cell-autonomous bistability (closed circles in Fig. S1C). For *ε*<2, the autonomous bistability disappears; instead, the whole population can only be at one of the two stable fixed points (red lines in Fig. 2B upper panel and closed circles in Fig. S1D). When the group average of the synthetase concentration 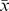 is greater than the value of the unstable fixed point (yellow line in Fig. 2B and open circle Fig. S1D), the entire population immediately jumps to the ON state. Otherwise, all of the cells converge to the OFF state. Thus, the value of *ε* determines the origin of the bistability and the form of the resulting group-level transition.

When *ε* is increased above 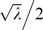 (Eq. S2-15 in Text S1), the intracellular signal concentration always exceeds the threshold regardless of the extracellular autoinducer concentration, so the cells are constitutively in the ON state at all cell densities (right of dashed line in Fig. 3A). Thus, autonomous bistability appears when 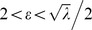 is satisfied (Eq. S2-15 in Text S1). The condition indicates that the region of the parameter *ε* that supports autonomous bistability (between the solid and dashed lines in Fig. 3A) broadens as *λ* is elevated, meaning that the bistability becomes less sensitive to variation in *ε*. In addition, when *λ* is decreased below *λ* = 9, the two stable states disappear and the system undergoes a pitchfork bifurcation. The cells thus become monostable at all cell densities (dotted line in Fig. 3A; Text S1 2.1). In summary, the analytical calculations indicate that autonomous bistability requires 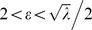, whereas group-level bistability requires both *λ*>9 and *ε*<2.

To clarify whether the above conditions for the two types of bistability directly translate into the conditions for group-level transitions, we examined whether group-level bistability always results in an all-or-none response and, similarly, whether autonomous bistability always gives rise to a graded response. This can be verified by checking whether or not the variability of the response is reduced by the secreted signal. To this end, we numerically measured the ratio between the coefficient of variation (CV) of the threshold cell density *ρ* (*CV_ρ_*; Fig. 2A–B; see Models) and the CV of the intrinsic heterogeneity of *k_i_* (*CV_k_*; Fig. S1A–B). Consistent with the above analysis, we see that *CV_ρ_*/*CV_k_*>1 for cell-autonomous bistability, indicating graded transitions (red and pink region in Fig. 3A). In contrast, for group-level bistability, *CV_ρ_*/*CV_k_* is almost always lower than unity, indicating a reduction in the variation (blue and cyan region in Fig. 3A). The condition *CV_ρ_*/*CV_k_* = 1 marks the borderline between the cell-autonomous and group-level switch for a wide range of growth rate (Model; Fig. S4). In addition, *CV_ρ_*/*CV_k_* decreases further as *ε* decreases and *λ* increases (Figs. 3A and S2B–C). At high *λ*, a state change within a small fraction of the population can elicit a sufficient increase in the extracellular signal concentration to override cell-cell variability in response sensitivity. Thus, the simulations show that while the effect of cell-cell variability is deleterious to simultaneous switch at low *λ* (Fig. S2D for *λ* = 10), the switch becomes more abrupt when *λ* is elevated (Fig. 1B for *λ* = 100). In group-level bistability, a large *λ* promotes all-or-none transitions by reducing the intrinsic heterogeneity (*CV_ρ_*/*CV_k_*<1). Thus, the conditions for autonomous (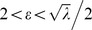) bistability and group-level (*λ*>9 and *ε*>2) bistability directly translate into the necessary conditions for the all-or-none and graded transitions, respectively.

The parameter region of autonomous bistability (between the solid and dashed lines in Fig. 3A) indicates robustness to variation in *ε*, while *CV_ρ_*/*CV_k_*<1 indicates robustness of group-level bistability to intrinsic variation of threshold *k_i_* (Figs. 3A, 2B and S2C–D). The robustness is further enhanced when we include an additional positive feedback in the model circuit (Eq. 3). First, in experimental observations of the synthetic *lux* gene circuits [23], the region of *ε* that supports autonomous bistability for the dual positive-feedback circuit (Eq. 3) is wider than that for the autoinduction circuit (Eq. 1). The bistable region further expands when the Hill coefficients, *m* and *n*, of AHL-LuxR and LuxR-promoter binding are increased (Figs. 3B and S3). We derived analytically that the boundary between autonomous bistability and the constitutively monostable state (dashed lines in Fig. 3B) is given by 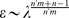
_,_ which monotonically increases with *m* and *n* (Eq. S2-23 in Text S1 2.3). In contrast, the boundary between autonomous bistability and group-level bistability is almost independent of *m* and *n* (*ε* = 2∼3; solid line in Fig. 3B; Eq. S2-23 in Text S1). Second, the value of *CV_ρ_*/*CV_k_* for the group-level bistability decreases further (Fig. S3) than that for the simple autoinduction circuit (Fig. 3A; e.g., at *λ* = 10∼100). Thus, the dual positive-feedback is highly effective in reducing the intrinsic variation. Such strengthening of group-level bistability explains the observation that a group-level switch of the rewired *lux* operon occurs much more abruptly in a dual positive-feedback circuit than in a simple autoinduction circuit [24]. In summary, in both the simple autoinduction and the dual positive-feedback circuits, a large amplification factor *λ* increases the robustness of both the graded and the all-or-none transitions.

### Optimality for using both transitions

Although microbial populations exhibit either graded [22], [23] or all-or-none [24] transitions, little is known about their benefit. Depending on the nature of environmental fluctuations, the coherence of cell-state transitions could significantly affect the chance of survival. When the environment varies more rapidly than the cellular response, the autonomous switch of individual cells could be more beneficial, because survival strategies can be diversified due to the heterogeneous response [48]: e.g., bistability in the expression of the *lac* gene in *E. coli* under certain growth conditions [49] and in the lysis/lysogeny decision of Lambda phage. In other words, the autonomous bistability is a bet-hedging or risk-spreading strategy in the population [50]. On the other hand, when the cells are able to respond as quickly as the environment changes, an all-or-none switch of the whole population allows more cells to survive and therefore could be a better strategy. Thus, depending on the time-scale of environmental fluctuations, being able to choose between autonomous and group-level switches provides an added advantage over a fixed survival strategy. The selection is more feasible when the order parameter *ε* of the population is close to the borderline; i.e. *ε* = 2. There, cells can choose between the two types of bistability by only slightly adjusting either the signal threshold, the maximum signal synthesis rate, or the transport rate (Eqs. 2 and S1-18 in Text S1).

To examine the survival strategy of bacterial species, we estimated the values of the parameter *ε* for four gene circuits in three bacterial species: the *rhl* and *las* operons in *P. aeruginosa*, the *car* operon in the plant pathogen *Erwinia carotovora*, and the *lux* operon in *V. fischeri* (Text S1 3). Each system has a dual positive-feedback network topology with cooperative gene regulation (Eq. 3; Table 1) [15]. We estimated the *c_sec_* and *γ_ex_* in Eq. 2 from the export and hydrolysis rates of AHL, respectively. We estimated the normalized threshold 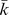 from the threshold signal concentration for gene expression within the operon with the extracellular signal concentration above a threshold density (Eq. S3-3 and Table S6). We found that not only do all QS systems analyzed fall within the appropriate range of *ε* that supports group-level or autonomous bistability (Fig. 3B), they also appear to converge on the boundary between the two types of bistability; i.e. *ε*∼2. The results suggest that bacteria could be adjusting the coherence of their state transitions in response to environmental conditions.

Several lines of evidence suggest that the parameters that determine *ε* are in fact being exploited in microbial populations. According to our estimate of the *lux* system (*ε* = 20∼30), the system should have a preference for a graded transition (Fig. 3B). Although this is true in *E. coli* harboring the synthetic *lux* system [22], [23], [27], all-or-none transition is observed in *V. fischeri* [29], [30]. This discrepancy could be caused by the fact that our estimate of the threshold concentration of an AHL 3-oxo-C6-HSL (corresponding to *k_i_* in Eq. 1) was based on a synthetic *lux* system in *E. coli*. In the real *lux* system of *V. fischeri*, an antagonist C8-HSL (HomoSerine Lactone) is endogenously synthesized and competitively binds to LuxR [51]. A microfluidic study of single *V. fischeri* cells showed that the presence of 100 nM C8-HSL increases the threshold concentration for 3-oxo-C6-HSL by as much as 10-fold [52]. Based on this evidence, we predict that, the addition of C8-HSL to synthetic *lux* systems should decrease *ε* by at least 10-fold and, as a consequence, would result in an all-or-none type transition. Likewise, the real *V. fischeri lux* system should exhibit a graded transition by eliminating C8-HSL or suppressing its synthesis.

Similarly, in the *las* system of *P. aeruginosa*, addition of an antagonist furanone, which eukaryotic cells produces to interfere with the bacterial QS [53], [54], suppresses the *las* gene expression [55] so that the concentration of the autoinducer 3-oxo-C12-HSL decreases. Conversely, the concentration of 3-oxo-C12-HSL is increased four-fold by the addition of a nutrient amino acid [31] which leads to inhibition of RNA synthesis [56] – bacterial survival strategy to avoid exhausting nutrients. Between *P. aeruginosa* stains that were clinically isolated from patients with severe polytrauma or congestive heart failure, there were large variations in the synthesized concentrations of 3-oxo-C12-HSL [57]. In addition, there was nine-fold decrease in threshold concentration of the *rhl* system in the absence of an antiactivator QslA [58]. The increase in autoinducer synthesis and the decrease in the threshold act to increase *ε* (Eqs. 2, S1-14 and S1-18 in Text S1) so that the graded transition is likely to emerge in the *las* and *rhl* systems. The heterogeneous response is in line with the fact that, in *P. aeruginosa* biofilms, the *las* and *rhl* systems are utilized for cell differentiation [59], [60]. Unlike laboratory conditions, nutrient conditions in natural habitats such as those surrounding biofilms inside animal hosts tend to fluctuate at various time scales [61]. Thus, by maintaining *ε*∼2, many bacterial populations may have the option of choosing between the two modes of transition by slightly changing their kinetic parameters.

### Relation to the onset of collective rhythmic behaviors

To further explore the applicability of the design principle (Fig. 3A) of group-level decision making, we introduced a negative feedback loop into the simple autoinducing circuit (Fig. 1E; Eq. 4). When the negative feedback takes place at a much slower time scale than the positive feedback does, qualitatively different dynamics may appear; the cells become oscillatory or excitable – ability to respond transiently to changes in the signal concentrations [62], [63]. Excitatory responses appear during the differentiation of *Bacillus subtilis* into the state of competence [40], the stress response of bacterial and mammalian cells [64], [65], the relay response of chemoattractant cyclic-AMP (cAMP) of *Dictyostelium discoideum* [38], the Ca^2+^ concentration response of pancreatic β cells [66], and the decision of the fate of embryonic stem cells [67]. When the cells are confined to a small chamber, the secreted signal becomes large that cells switch from a quiescent state to a rhythmic state as a group (Fig. 4).

The oscillatory transition is referred to as dynamical quorum sensing (DQS) [34]–[39]. The presence of quiescent cells at low density in DQS is a marked contrast to the Kuramoto-type transition [68]–[71], where all cells are independently oscillatory and the transition to a collective state is realized by phase synchronization. While such a transition is believed to take place in populations of fireflies [72] and in the neurons of the mammalian suprachiasmatic nucleus [73], other examples have shown a state of quiescence at low cell density [38], [74], [75]. Individual *Dictyostelium* cells do not exhibit cAMP oscillations at low density, and they only become oscillatory above a certain density [38]. A slightly different case is found in the NADH oscillations of *Saccharomyces cerevisiae*, where the fraction of oscillatory cells gradually increases when the dilution rate of secreted factors is decreased [75].

The parameter *ε* (Models; Eqs. 2 and S1-24 in Text S1) in DQS also determines whether the transition is graded or all-or-none. As shown by the numerical simulations, when *ε* is high the transition is graded (Fig. 4A); a fraction of cells oscillate individually, whereas the others remain quiescent. As in the bistable circuits, cells become autonomously oscillatory when the intracellular autoinducer concentration (*γ_ex_ x_i_/c_sec_*) exceeds the threshold *k_i_* (Eq. 4). Because of intrinsic cell-cell heterogeneity in the sensitivity threshold *k_i_* (Fig. S5A), a small fraction of the population is already oscillatory even at low cell densities (Figs. 4A and S5C). As we have seen in the bistable system (Fig. 2A), the proportion of oscillatory cells gradually increases with increasing cell density (Fig. 4A). Accordingly, while the amplitude of a single cell is kept constant (local maximum of the blue line in Fig. 4A), the amplitude of the cellular ensemble gradually increases (red line in Fig. 4C). Such gradual increases in the mean amplitude have been observed in engineered *E. coli* [35] and in the glycolytic oscillations of yeasts [37].

The oscillatory transition is all-or-none when *ε* is low: all cells simultaneously switch to the oscillatory state above a threshold cell density (Fig. 4B; see also Fig. S5D for the density dependence of the nullclines). Moreover, at the onset of oscillations, the pulse is highly synchronized among the cells (black dots in Fig. 4B). Note that this occurs despite the presence of cell-cell heterogeneity in the response threshold (Fig. S5B). An all-or-none transition is observed as both an abrupt increase in the oscillation amplitude averaged over the population (red line in Fig. 4B) as well as an increase in the fraction of oscillatory cells (Fig. 4D). A group-level excitatory response to a common level of signaling molecule is responsible for the all-or-none transition in DQS. There are almost no cells that oscillate below the threshold density, because the synthesized concentration *γ_ex_ x_i_/c_sec_* is below the threshold *k_i_* regardless of *x_i_* (Eq. 4). When a certain fraction of the population is excited because of cell-cell variability in *k_i_*, a subsequent increase in the secreted signal 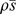 invokes the excitation of the remaining population. Thus, the positive feedback supports a chain reaction of excitatory responses, because the secreted signals mutually enhance the excitation of other cells (time ∼3600 in Fig. 4B). Such group-level excitation captures the essence of what has been observed in the abrupt transition from quiescence to highly synchronized oscillations in particle-based Belouzov-Zhabotinsky reactions [76] and in the cAMP signaling of *Dictyostelium* [38].

**Figure 5:**
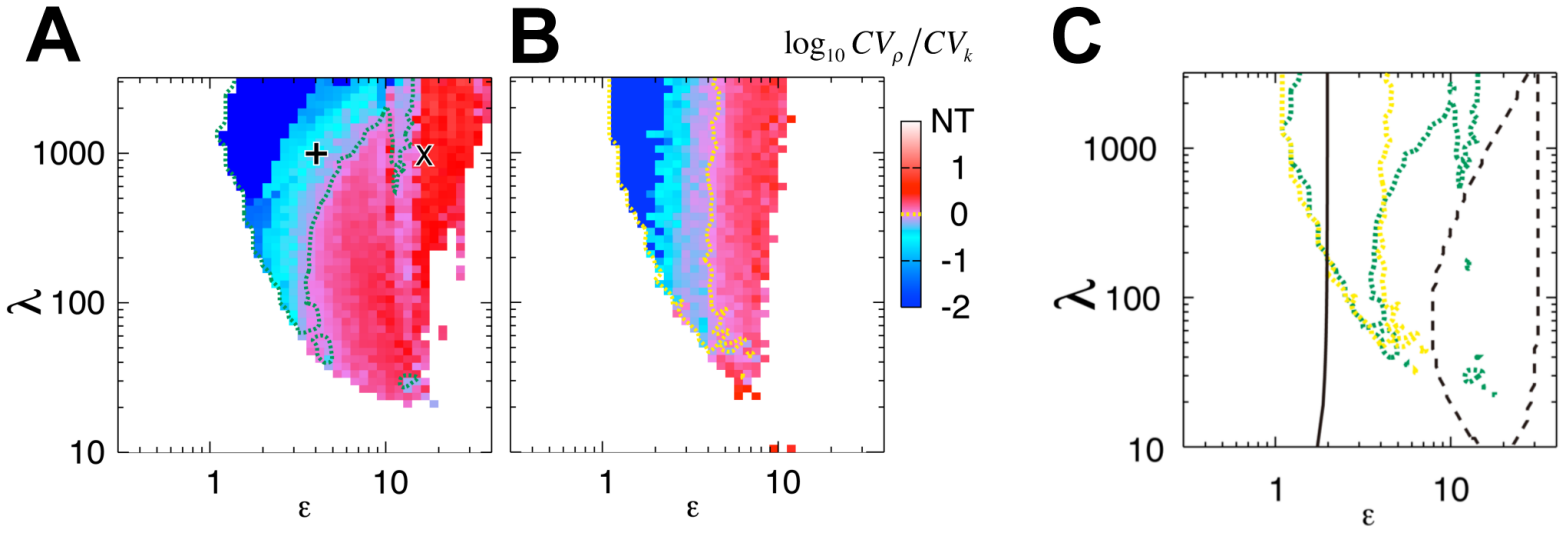
Conditions for collective decision making in dynamical quorum sensing. (**A–B**) *CV_ρ_*/*CV_k_* (see Models) in the absence (**A**, |*η_i_*| = 0) or the presence (**B**, |*η_i_*| = 0.1) of noise in a positive-and-negative feedback circuit (Eq. 4). NT: No Transition, i.e., constitutive quiescence or constitutive oscillations irrespective of cell density (white region). The × and + correspond to Figs. 4A and 4B, respectively. (**C**) Log_10_ *CV_ρ_*/*CV_k_* = 0 in the absence (green curve) and the presence (yellow curve) of noise. The black solid line indicates the boundary *ε*∼2 (Eq. S2-27 in Text S1). The region surrounded by the black dotted curves marks the parameter region where the cells are autonomously oscillatory in an isolated condition (*ρ* = 0). The values of the parameters are the same as those in Fig. 4.

Following the argument for the coupled bistable circuits described above (Fig. 3A), the nature of oscillatory transitions in the coupled excitable circuits could also be numerically classified by *CV_ρ_*/*CV_k_* (Fig. 5A); i.e., whether or not the intrinsic variability of threshold *k_i_* is reduced: all-or-none when *CV_ρ_*/*CV_k_*<1 (blue and cyan in Fig. 5A) and graded when *CV_ρ_*/*CV_k_*>1 (red and pink in Fig. 5A). The boundary between the transition types (*CV_ρ_*/*CV_k_* = 1; green line in Fig. 5A) is located between *ε* = 2 and *ε* = 10. *ε*>10 roughly corresponds to the necessary condition for cell-autonomous oscillations (black dashed line in Fig. 5C), while *ε*>2 is shown analytically to be the necessary condition for cell-autonomous excitation in isolated cells (black solid line in Fig. 5C; Fig. S6A; see Text S1 2.4 for a derivation). To examine the role of autonomous excitability, intrinsic noise is introduced into the kinetics of the synthetase (*η_i_* in Eq. 4), as was done for the bistable circuit. At *ε*>2, the cells are repetitively excited by the intrinsic signal noise rather than by the secreted signal, so there are cell-autonomous stochastic pulses frequently observed in excitable systems [38], [77]. Thus the transition becomes graded (right of the yellow line indicating *CV_ρ_*/*CV_k_* = 1 in Fig. 5B–C). The convergence of the boundary to *ε* = 2 in the presence of noise occurs irrespective of the remaining free parameter *g* (yellow line in Fig. S6B–E). Thus, the autonomous excitation and oscillation mediated by the intracellular feedback lead to graded transitions; whereas the group-level excitation mediated by the secreted autoinducer invokes all-or-none transitions to highly synchronized oscillations. Moreover, the position of the boundary (*ε*∼2, Figs. 5B–C and S6B–E; Eq. S2-27 in Text S1) agrees well with that obtained for the bistable circuits (Fig. 3A; Eq. S2-15 in Text S1), indicating that the relative contributions to the feedback from the autoinducer that is synthesized and accumulated within the cell and that which is secreted and shared with other cells are the key determinants of the group-level transition. In the engineered *E. coli* with a positive-and-negative-feedback (Fig. 1E; Eq. 4) mediated by the *lux* system [35], it is reasonable to expect *ε*>2 (SI Text 3.2.4), since the expression of *ε* is identical with that of the autoinduction circuit (Eq. 2; Eq. S1-24). As a result, the mean amplitude increases gradually with cell density (Fig. 4A). This suggests that the oscillatory transition in the engineered *E. coli*. [35] is graded.

### Limitation of the models

Future works should clarify the limit and applicability of the common design principle elucidated in this study by exploring more complex circuit topologies in a wide variety of biological contexts. Our models did consider spatial heterogeneity of the extracellular autoinducer concentration that could potentially form a spatial gradients [78] or propagating waves [35], [79] (Models). The spatial heterogeneity becomes important, for example when we consider spatial structure of microbial colonies, aggregates or biofilms with a diameter of more than 1 mm (Text S1 1.6). The autonomous bistability presented here faithfully reproduces microbial group-level dynamics such as the bimodal distribution (Fig. 2C; [22], [23]) and the continuous increase in the fraction of ON cells as cell density increase (Fig. 2A upper panel; [25], [28]). We should note, however, that there may also be other types of bistability. In *V. harveyi*, the maximum fraction of the ON-state cells never reaches 100% even at high densities [25]. It also appears that not all *V. fischeri* cells can exhibit state transition when isolated in a chamber and perfused with high dosages of autoinducer [10]. Such a property could be due to either a large variability in the threshold value *k_i_*, presence of an antagonist [52] that suppresses autoinducer synthesis, or another negative feedback that adds a repressive cell-cell interaction [80]–[82] so as to render coexistence of ON and OFF cells (Fig. 1E) more likely in a wide range of model parameters. Delineating these possibilities will be an important avenue for future studies.

### Application to signal transduction via a transmembrane receptor

To further test applicability of the common design principle, we expanded the simple transport system for the autoinducer (Fig. 1C–E) to describe transmembrane signal recognition and transduction [15], [32], [83]. For transmembrane recognition systems, in addition to the extracellular feedback of the autocrine signaling, an intracellular positive feedback is required for a graded transition (Text S1 1.4), as in the simple autoinduction (Eq. 1) and the dual positive-feedback circuits (Eq. 3). Consistently, the parameter *ε* tunes the graded and all-or-none transitions in QS (Fig. S7 and Eq. S1-33 in Text S1) as well as in DQS (Fig. S8). Hence, the design principle should be widely applicable to cell density-dependent fate decisions [84] in a broad spectrum of cell populations; e.g., in animal embryogenesis [20], [21], stem-cell differentiation in tissue engineering [85], [86], influenza virus infection [87], and cancer metastasis [88].

### Conclusion

We have seen that when individual cells alone can harbor dynamic stabilities, the transition at the group level becomes graded (Figs. 2A and 4A). These dynamic stabilities are cell-autonomous bistability, in the case of autoinducing circuits, and cell-autonomous excitability, in case of negative-feedback circuits (Figs. 2B and 4B). In contrast, group-level all-or-none transitions between cellular states are supported when these stabilities require a sufficient number of cells. In both bistable circuits and excitable circuits (Fig. 1C–E), the two parameters *ε* and *λ* determine the transition type (Figs. 3 and 5). For the cells to switch their states, inducing molecules need to accumulate to a certain level within the group. *ε* compares the contribution of intracellular local feedback with that of secretion-mediated global feedback. For *ε*>2, bistability or excitability can be reduced to a single-cell property. For *ε*<2, the switch requires group-level cooperation mediated by secreted signaling molecules. The necessary conditions for autonomous and group-level stabilities are directly translated into those for graded and all-or-none transitions, respectively (Figs. 3A and 5C). The greater the amplification factor *λ* is, the more robust the transitions are to cell-cell variability (Figs. 3A and S2C) and parameter variations (Fig. 3B). Future studies should be able to experimentally verify this design principle by tuning *λ* and *ε* with inducible promoters [27], [40] or by applying agonists and antagonists to the system [52], [54], [55].

## Supporting Information

Figure S1
**Supporting figure for** **Figure 2** **.** (**A**)–(**B**) Heterogeneous response of isolated cells (N = 100) to exogenously applied autoinducer due to cell-cell heterogeneity in *k_i_*,. 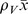 in Eq. 1 is replaced by exogenous signal concentration *s_e_*. (**C**)–(**D**) Nullclines of Eq. 1 in case of two cells, where the values of 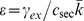 and *λ* are identical with those used in Figs. 2A and 2B, respectively. Closed and open circles indicate stable and unstable fixed points, respectively. At the both stable fixed points, *x_1_* and *x_2_* are identical indicating group-level bistability. (*x_1_*, *x_2_*)∼(1, 0.01) and (0.01, 1) are also allowed in (**B**) indicating coexistence of ON and OFF states. (**E**)–(**F**) Activity of synthetase *dx/dt* plotted as a function of *x*; autonomous (**E**; Eq. S2-16) and group-level (**F**; Eq. S2-7) bistability. The values of *ε* in (**E**) and (**F**) are identical with Figs. 2A and 2B, respectively.(TIF)

Figure S2
**Supporting figure for** **Figure 3A** **. (A)** Phase boundaries AB/GB (solid line), AB/M (dashed line), and GB/M (dotted line) determined analytically (black, Eqs. S2-6 and S2-14) and numerically (grey). In numerical simulations of cell population (Eq. 1), the AB phase is assigned when the ON- and OFF-state cells coexisted at the steady state. The GB phase is assigned when the entire population uniformly takes either the ON- or the OFF state at all density. **(B)–(C)** *CV_ρ_*/*CV_k_* plotted as a function of *ε* for *λ* = 100 and 1000 **(B)** and *λ* for *ε* = 0.25 **(C)**, respectively, for the simple autoinduction circuit (Eq. 1). In case of group-level bistabiltiy (*ε*<2 in **(B)**; red points in **(C)**), *CV_ρ_*/*CV_k_* decreases with decreasing *ε* and increasing *λ*. On the other hand, *CV_ρ_* is approximately equal to the intrinsic variation *CV_k_*, when the group-level bistability disappears (*ε*>2 in **(B)** for autonomous bistability; grey points in **(C)** represent simple autoinduction without cooperativity 
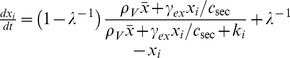
). **(D)** The response of synthetase concentration *x_i_* to cell density *ρ* is highly variable between the cells at *λ* = 10 compared to *λ* = 100 (Fig. 2B). The value of *ε* and standard deviation of *k_i_* are the same as Fig. 2B. Each point in indicates time average at the steady state.(TIF)

Figure S3 **Supporting figure for** **Figure 3B** **.** (**A**)–(**C**) Phase diagram of *CV_ρ_*/*CV_k_* in dual positive-feedback circuit (Eq. 3). (*m, n*) = (1, 1) (**A**), (2, 1) (**B**), and (2, 2) (**C**), respectively. Solid, dashed and dotted black lines indicate analytically determined boundary AB/GB (Eq. S2-23), AB/M (Eq. S2-23), and GB/M, respectively. Yellow line is log_10_ *CV_ρ_*/*CV_k_* = 0 determined numerically.
(TIF)

Figure S4 **Growth rate dependence of the phase diagram.** The boundary line between all-or-none and graded transition, i.e., *CV_ρ_*/*CV_k_* = 1 is plotted for three different increasing rate of cell density, i.e., growth rate (Models). The ratio of the increasing rate to the degradation rate of synthetase (*γ_X_* in Eq. S1-3) is set to 1/2 (red), 1/10 (blue) and 1/40 (yellow), respectively, for the simple autoinduction circuit (**A**; Fig. 1C) and the dual positive-feedback circuit (**B–C**; Fig. 1D). The yellow line was imported from Figs. 3A. S3A, and S3B to (**A**), (**B**) and (**C**), respectively. The boundary lines are almost independent of the growth rate.
(TIF)

Figure S5
**Supporting figure for** **Figure 4** **.** (**A**)–(**B**) Heterogeneous response of synthetase concentration *x_i_* in isolated cells (blue line: time course of a representative cell; light blue point: pulsatile response of each cell indexed in Y-axis). 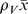 in Eq. 4 is replaced by the extracellular autoinducer concentration *s_e_* that is applied exogenously as an exponentially increasing function (violet line). *ε* = 16 in (**A**) and 4 (**B**) as in Figs. 4A and 4B, respectively. *λ* = 10^3^ and *g* = 30 in (**A**) and (**B**). At *s_e_* = 0, a fraction of cells are already oscillatory in (**A**), whereas all cells are quiescent in (**B**). (**C**)–(**D**) Nulcllines of isolated condition (Eq. S1-26) for *ρ* = 0 and population mean (Eq. S1-28) for *ρ* = 1. Value of the parameters in (**C**) and (**D**) are same with (**A**) and (**B**) respectively. At *ρ* = 0, the i-th cell is either excitatory or oscillatory depending on *k_i_* (**C**). In (**D**), cells are always excitatory regardless of *k_i_*. At *ρ* = 1, all cells are oscillatory in both (**C**) and (**D**). Red and blue lines indicate *dx/dt* = 0 and *dy/dt* = 0, respectively. Solid and dotted lines at *ρ* = 0 indicate *k_i_* is 50% larger and smaller than 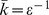 (Eq. S1-24).(TIF)

Figure S6 **Supporting figure for** **Figure 5** **. (A)** The necessary condition for cell-autonomous excitability. *dx_i_/dt* = 0 in isolated condition (Eq. S1-26) for *ε* = 1, 2 and 4 (1/*k_i_* = *ε* in Eq. S1-24). For ease of view, *dy_i_/dt* = 0 is plotted only for *ε* = 4 (light blue line). The local maximum (*x_i_*∼0.5) is positive at *ε* = 4, zero at *ε* = 2 and negative at *ε* = 1 indicating no excitability at *ε* = 1 and 2. Thus cell-autonomous excitability requires *ε*>2, consistent with analytical derivation (Text S1 2.4). **(B)–(C)** Phase diagram of *CV_ρ_*/*CV_k_* for *g* = 3 **(B)** and 10 **(C)** in presence of intrinsic noise (*η_i_* in Eq. 4; |*η_i_*| = 0.1). The other parameters are identical with Fig. 5. **(D)–(E)** log_10_ *CV_ρ_*/*CV_k_* = 0 plotted for cases with noise (yellow dotted line) or without (green dotted line). Black solid line indicates the excitable/oscillatory boundary *ε*∼2 derived analytically (Eq. S2-27). The region surrounded by black dashed curves supports autonomous oscillations in isolated condition in numerical simulations. *g* = 3 **(D)** and 10 **(E)**.
(TIF)

Figure S7 **Graded and all-or-none quorum sensing transitions in a trans-membrane receptor model.** **(A)** Schematics of an autoinduction circuit with transmembrane receptor model (Eq. S1-32) in operation. **(B)–(C)** Heterogeneous response in isolated cells to exogeneously applied autoinducer signal (Eq. S1-32; *k_i_* has intrinsic variability as in Eq. 1; |*η_i_*| = 0.1). *ε* = 2.5 **(B)** and 0.25 **(C)**. *λ* = 100. Bistability appears cell-autonomously in **(B)** but not in **(C)**. **(D)** Autonomous bistability and **(E)** group-level bistability (red line, analytical solution for the population mean) underlie graded and all-or-none transitions, respectively. Parameters in **(D)** and **(E)** are identical with **(B)** and **(C)**, respectively. **(F)** Phase diagram of *CV_ρ_*/*CV_k_*. Solid, dashed and dotted black lines indicate analytically determined boundary AB/GB, AB/M, and GB/M, respectively. Yellow line is log_10_ *CV_ρ_*/*CV_k_* = 0 determined numerically. × and + correspond to **(D)** and **(E)**, respectively.
(TIF)

Figure S8
**Dynamical quorum sensing transitions in a negative-feedback mediated trans-membrane receptor model.** **(A)** Schematics of positive-and-negative feedback circuit with transmembrane receptor model (Eq. S1-34). **(B)–(C)** Nullclines for the isolated cell (Eq. S1-36; left panel) and the population (Eq. S1-37; right panel) predict transitions from quiescence to oscillations depending on cell density *ρ*. Similar to the direct import model (Eq. 4; Fig. S5C–D), at *ρ* = 0, cells are either excitatory or oscillatory depending on *k_i_* in case of *ε*  = 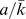 = 5.6 **(B)**. Cells are always excitatory irrespective of *k_i_* in *ε* = 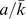 = 2.8 **(C)**. Solid and dotted lines at *ρ* = 0 indicate *k_i_* is 25% larger and smaller than 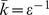 (Eq. S1-24). *α* = 2.4, *β* = 10, and *λ* = 100 in (**B)** and **(C)**. **(D)–(E)** Simulations of communicating cell populations demonstrate graded **(D)** and all-or-none **(E)** transitions during exponential increase in cell density *ρ* (grey line). The randomized parameter *k_i_* has a lognormal distribution with *CV_k_* = 0.25. *ε* = 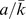 in **(D)** and **(E)** are the same with (**B)** and **(C)**, respectively. *λ*, *α*, and *β* in **(D)** and **(E)** are also identical with **(B)** and **(C)**. *α_y_* = 100. |*η_i_*| = 0.0.(TIF)

Table S1 **Representative examples of autoinduction.**
(TIFF)

Table S2 **List of variables and parameters of autoinduction kinetics Eq. S1-1 to Eq. S1-3.**
(TIFF)

Table S3 **Parameter values chosen in dual positive-feedback circuit** **Eq. 3** **.**
(TIFF)

Table S4 **Parameter values chosen in positive-and-negative feedback circuit** **Eq. 4** **.**
(TIFF)

Table S5 **Dimensional analysis of spatial scale of extracellular environment.**
(TIFF)

Table S6 **Parameters estimated from literatures.**
(TIFF)

Text S1 **Supporting methods and results.**
(PDF)
