# Supplementary figures and images for: A Design Principle of Group-level Decision Making in Cell Populations
Source: PLoS Comput Biol. 2013 Jun 27;9(6):e1003110. doi: 10.1371/journal.pcbi.1003110 (PMC3694814; doi:10.1371/journal.pcbi.1003110)

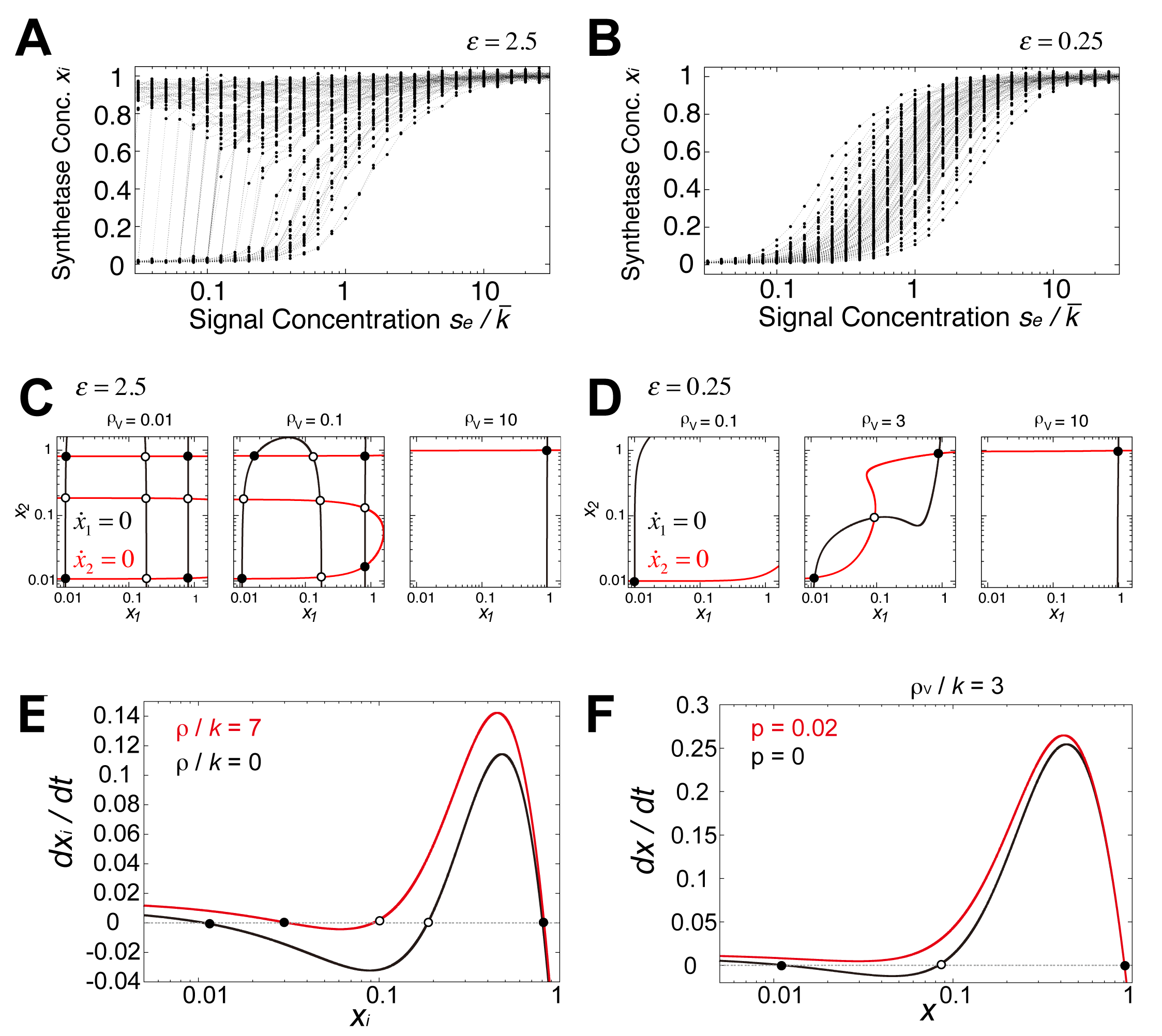

Supplement: Figure S1 — Supporting figure for Figure 2 . (A)–(B) Heterogeneous response of isolated cells (N = 100) to exogenously applied autoinducer due to cell-cell heterogeneity in ki,. in Eq. 1 is replaced by exogenous signal concentration se. (C)–(D) Nullclines of Eq. 1 in case of two cells, where the values of and λ are identical with those used in Figs. 2A and 2B, respectively. Closed and open circles indicate stable and unstable fixed points, respectively. At the both stable fixed points, x1 and x2 are identical indicating group-level bistability. (x1, x2)∼(1, 0.01) and (0.01, 1) are also allowed in (B) indicating coexistence of ON and OFF states. (E)–(F) Activity of synthetase dx/dt plotted as a function of x; autonomous (E; Eq. S2-16) and group-level (F; Eq. S2-7) bistability. The values of ε in (E) and (F) are identical with Figs. 2A and 2B, respectively. (TIF) [file pcbi.1003110.s001.tif]

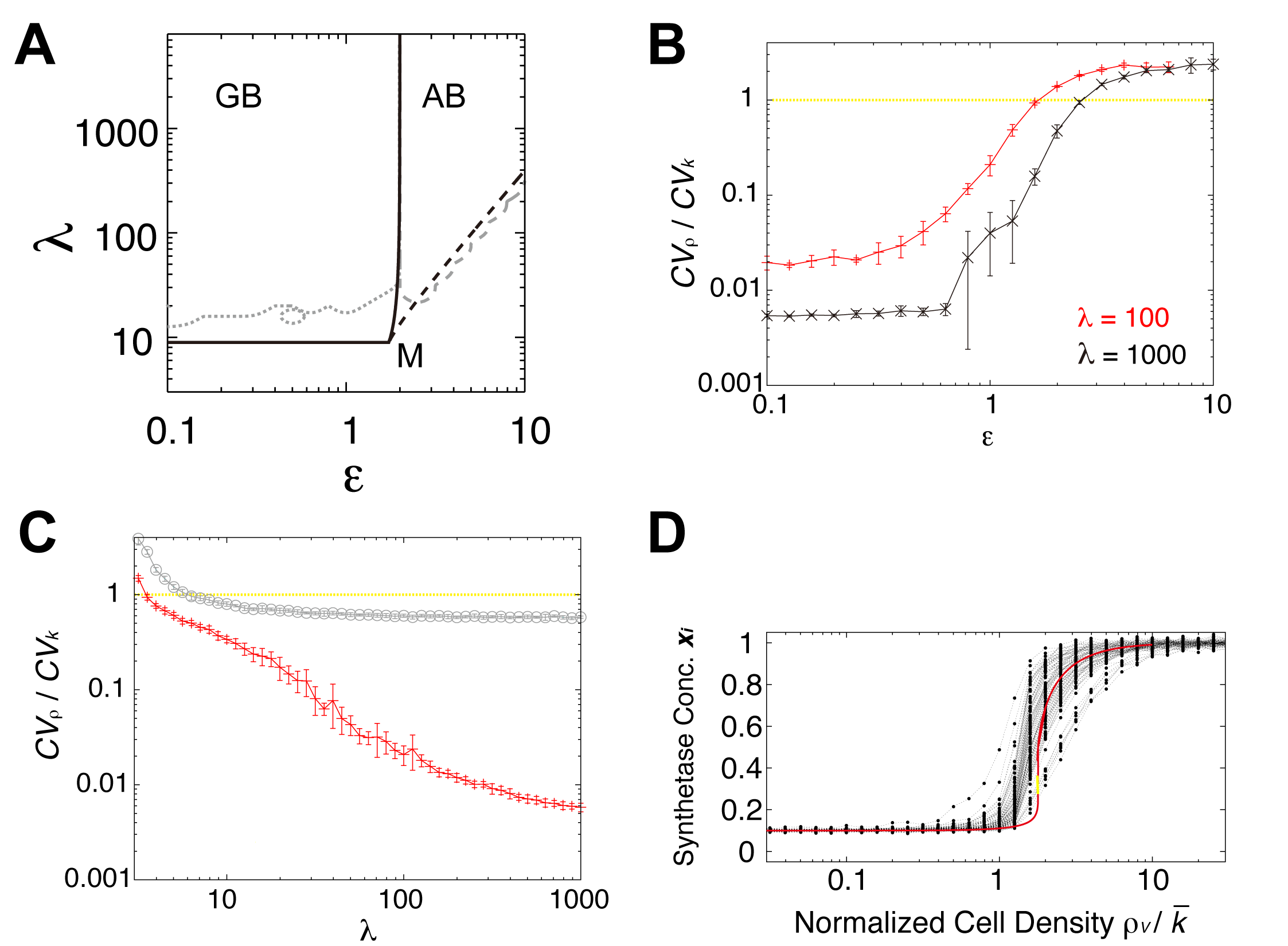

Supplement: Figure S2 — Supporting figure for Figure 3A . (A) Phase boundaries AB/GB (solid line), AB/M (dashed line), and GB/M (dotted line) determined analytically (black, Eqs. S2-6 and S2-14) and numerically (grey). In numerical simulations of cell population (Eq. 1), the AB phase is assigned when the ON- and OFF-state cells coexisted at the steady state. The GB phase is assigned when the entire population uniformly takes either the ON- or the OFF state at all density. (B)–(C) CVρ/CVk plotted as a function of ε for λ = 100 and 1000 (B) and λ for ε = 0.25 (C), respectively, for the simple autoinduction circuit (Eq. 1). In case of group-level bistabiltiy (ε<2 in (B); red points in (C)), CVρ/CVk decreases with decreasing ε and increasing λ. On the other hand, CVρ is approximately equal to the intrinsic variation CVk, when the group-level bistability disappears (ε>2 in (B) for autonomous bistability; grey points in (C) represent simple autoinduction without cooperativity ). (D) The response of synthetase concentration xi to cell density ρ is highly variable between the cells at λ = 10 compared to λ = 100 (Fig. 2B). The value of ε and standard deviation of ki are the same as Fig. 2B. Each point in indicates time average at the steady state. (TIF) [file pcbi.1003110.s002.tif]

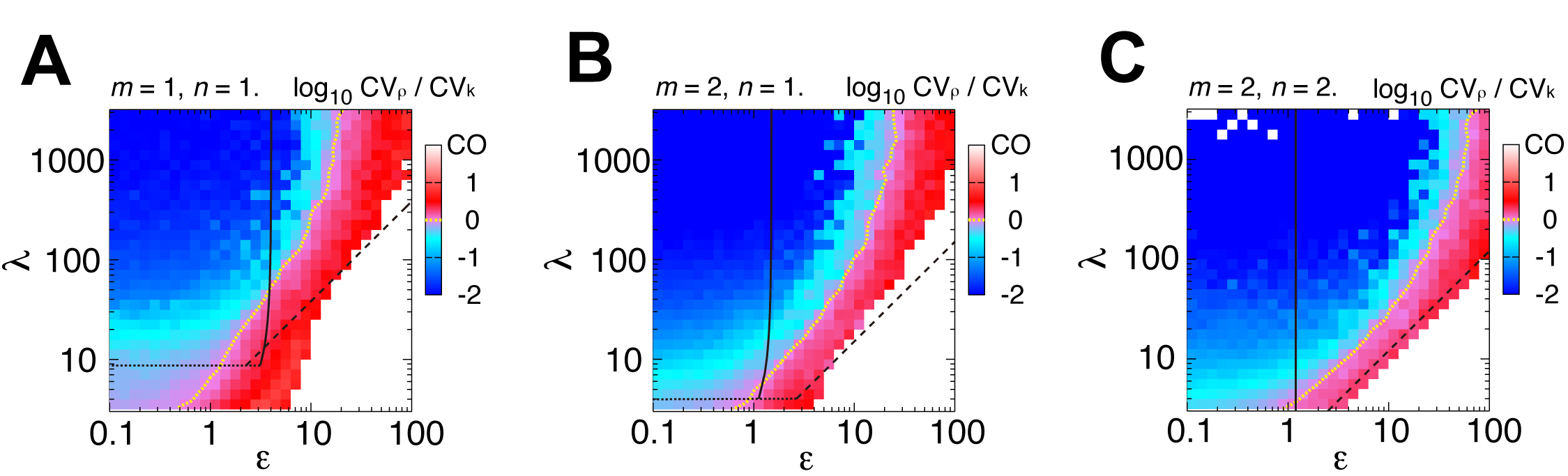

Supplement: Figure S3 — Supporting figure for Figure 3B . (A)–(C) Phase diagram of CVρ/CVk in dual positive-feedback circuit (Eq. 3). (m, n) = (1, 1) (A), (2, 1) (B), and (2, 2) (C), respectively. Solid, dashed and dotted black lines indicate analytically determined boundary AB/GB (Eq. S2-23), AB/M (Eq. S2-23), and GB/M, respectively. Yellow line is log10 CVρ/CVk = 0 determined numerically. (TIF) [file pcbi.1003110.s003.tif]

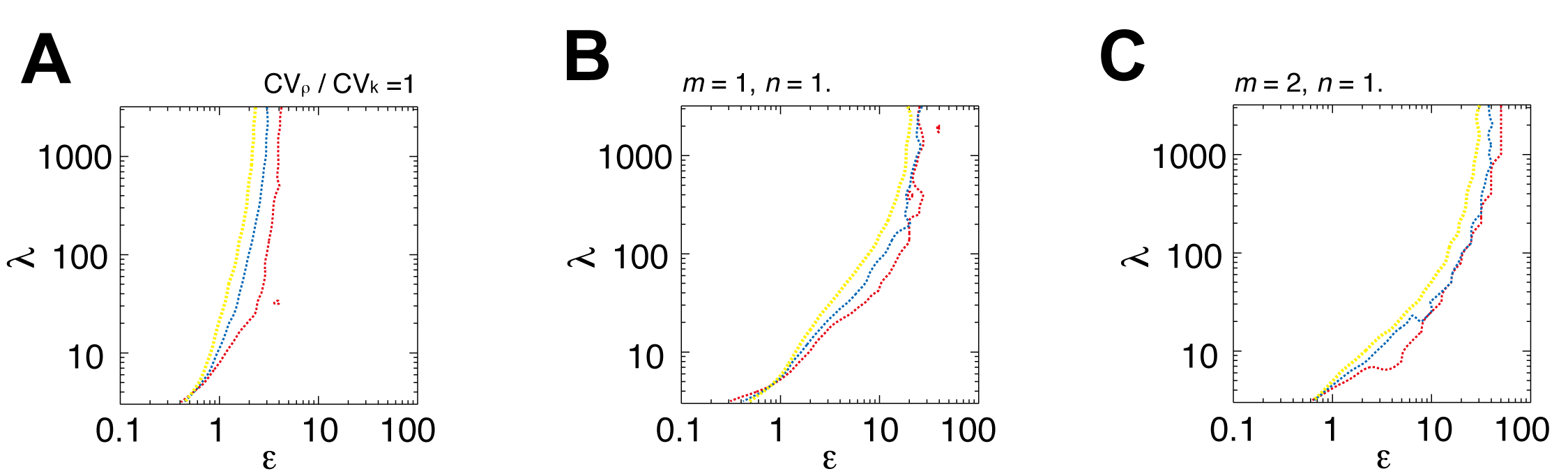

Supplement: Figure S4 — Growth rate dependence of the phase diagram. The boundary line between all-or-none and graded transition, i.e., CVρ/CVk = 1 is plotted for three different increasing rate of cell density, i.e., growth rate (Models). The ratio of the increasing rate to the degradation rate of synthetase (γX in Eq. S1-3) is set to 1/2 (red), 1/10 (blue) and 1/40 (yellow), respectively, for the simple autoinduction circuit (A; Fig. 1C) and the dual positive-feedback circuit (B–C; Fig. 1D). The yellow line was imported from Figs. 3A. S3A, and S3B to (A), (B) and (C), respectively. The boundary lines are almost independent of the growth rate. (TIF) [file pcbi.1003110.s004.tif]

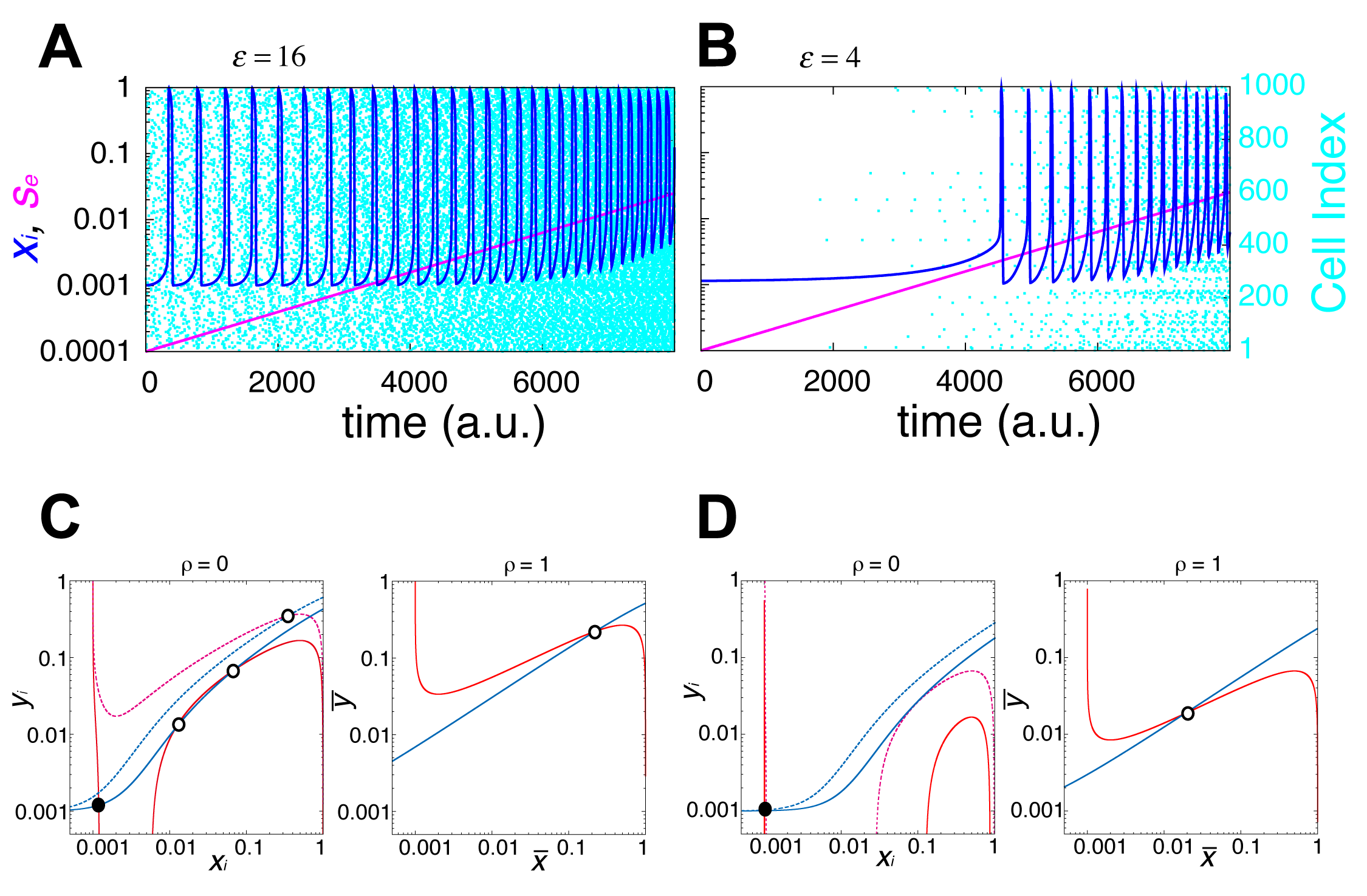

Supplement: Figure S5 — Supporting figure for Figure 4 . (A)–(B) Heterogeneous response of synthetase concentration xi in isolated cells (blue line: time course of a representative cell; light blue point: pulsatile response of each cell indexed in Y-axis). in Eq. 4 is replaced by the extracellular autoinducer concentration se that is applied exogenously as an exponentially increasing function (violet line). ε = 16 in (A) and 4 (B) as in Figs. 4A and 4B, respectively. λ = 103 and g = 30 in (A) and (B). At se = 0, a fraction of cells are already oscillatory in (A), whereas all cells are quiescent in (B). (C)–(D) Nulcllines of isolated condition (Eq. S1-26) for ρ = 0 and population mean (Eq. S1-28) for ρ = 1. Value of the parameters in (C) and (D) are same with (A) and (B) respectively. At ρ = 0, the i-th cell is either excitatory or oscillatory depending on ki (C). In (D), cells are always excitatory regardless of ki. At ρ = 1, all cells are oscillatory in both (C) and (D). Red and blue lines indicate dx/dt = 0 and dy/dt = 0, respectively. Solid and dotted lines at ρ = 0 indicate ki is 50% larger and smaller than (Eq. S1-24). (TIF) [file pcbi.1003110.s005.tif]

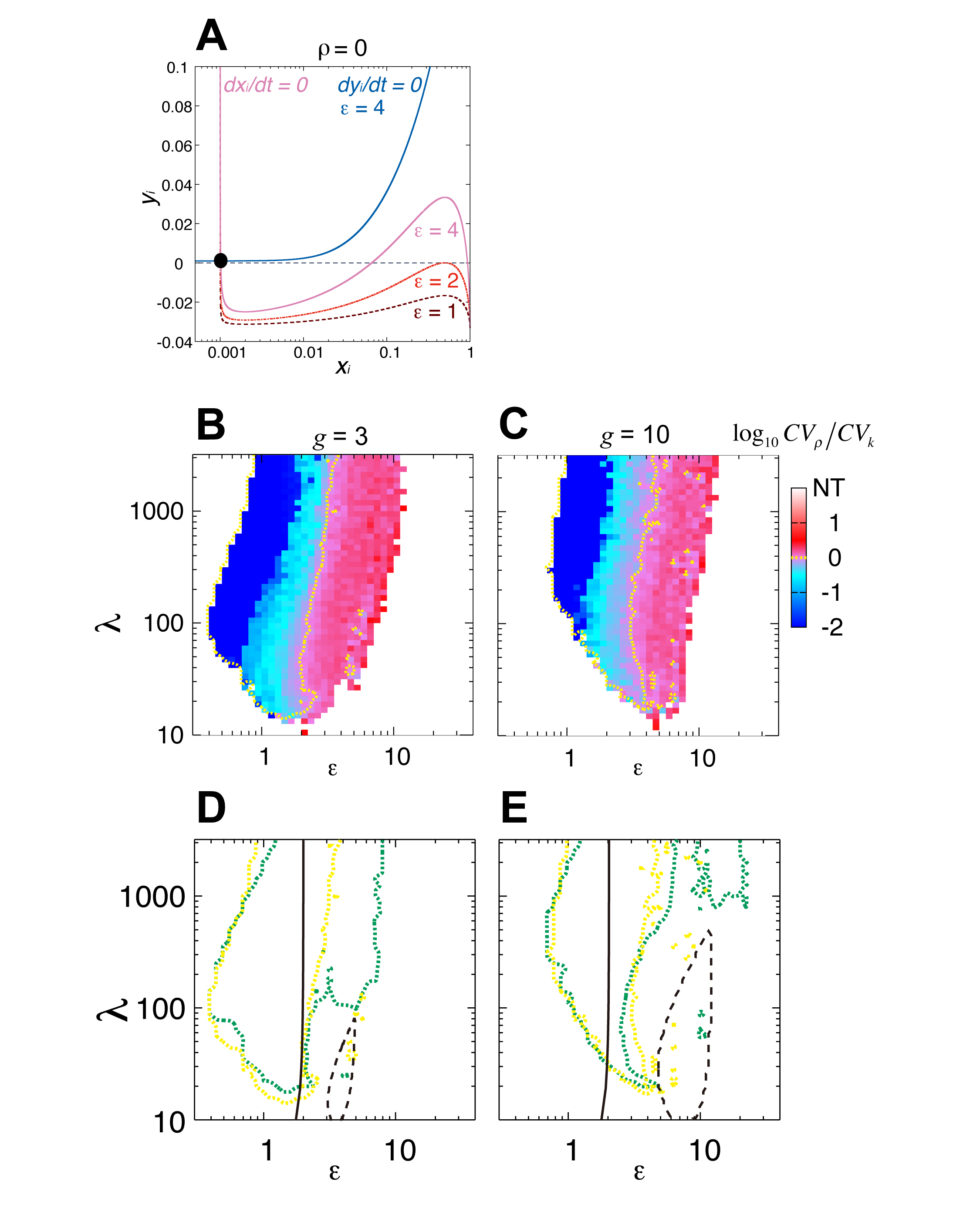

Supplement: Figure S6 — Supporting figure for Figure 5 . (A) The necessary condition for cell-autonomous excitability. dxi/dt = 0 in isolated condition (Eq. S1-26) for ε = 1, 2 and 4 (1/ki = ε in Eq. S1-24). For ease of view, dyi/dt = 0 is plotted only for ε = 4 (light blue line). The local maximum (xi∼0.5) is positive at ε = 4, zero at ε = 2 and negative at ε = 1 indicating no excitability at ε = 1 and 2. Thus cell-autonomous excitability requires ε>2, consistent with analytical derivation (Text S1 2.4). (B)–(C) Phase diagram of CVρ/CVk for g = 3 (B) and 10 (C) in presence of intrinsic noise (ηi in Eq. 4; |ηi| = 0.1). The other parameters are identical with Fig. 5. (D)–(E) log10 CVρ/CVk = 0 plotted for cases with noise (yellow dotted line) or without (green dotted line). Black solid line indicates the excitable/oscillatory boundary ε∼2 derived analytically (Eq. S2-27). The region surrounded by black dashed curves supports autonomous oscillations in isolated condition in numerical simulations. g = 3 (D) and 10 (E). (TIF) [file pcbi.1003110.s006.tif]

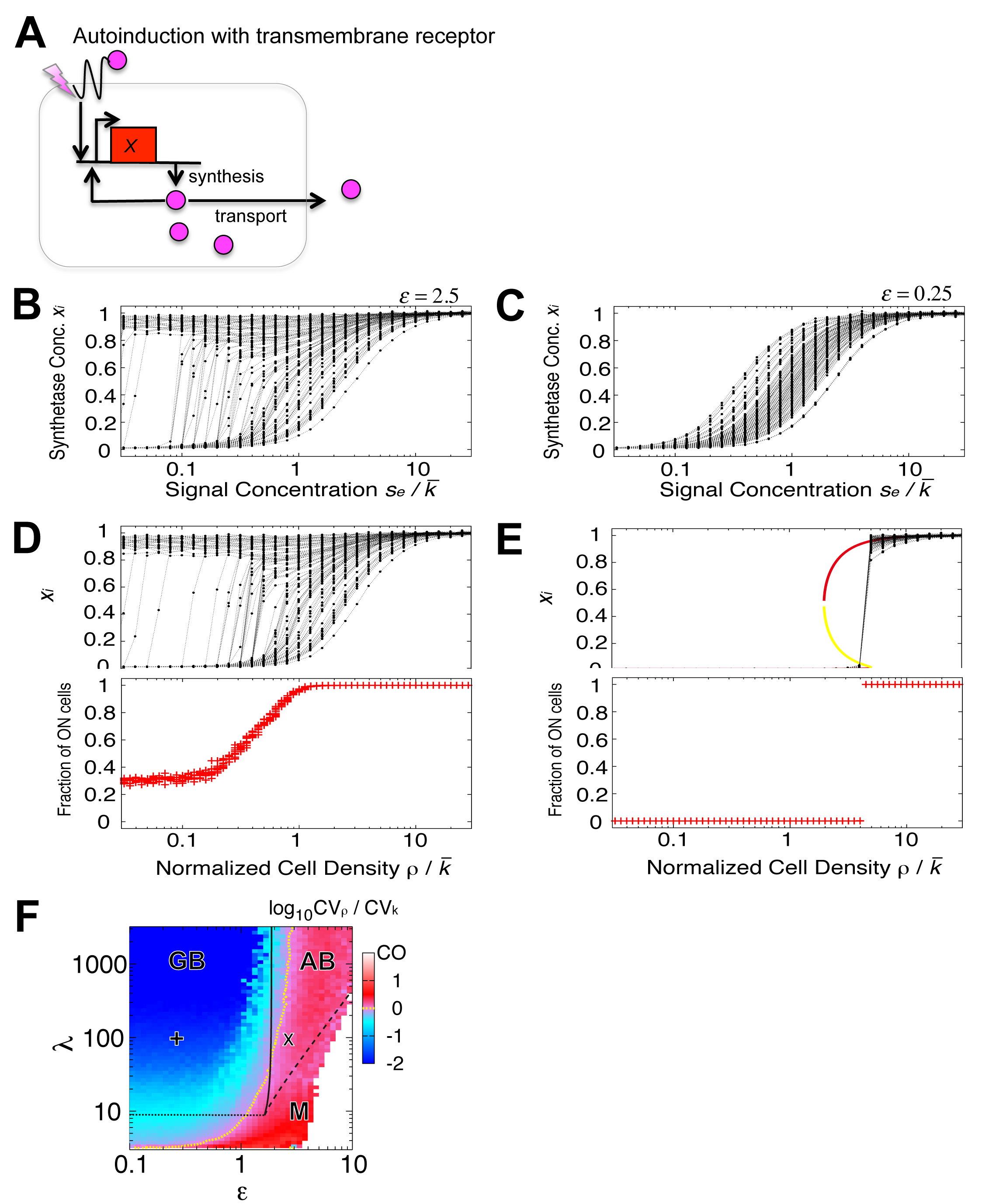

Supplement: Figure S7 — Graded and all-or-none quorum sensing transitions in a trans-membrane receptor model. (A) Schematics of an autoinduction circuit with transmembrane receptor model (Eq. S1-32) in operation. (B)–(C) Heterogeneous response in isolated cells to exogeneously applied autoinducer signal (Eq. S1-32; ki has intrinsic variability as in Eq. 1; |ηi| = 0.1). ε = 2.5 (B) and 0.25 (C). λ = 100. Bistability appears cell-autonomously in (B) but not in (C). (D) Autonomous bistability and (E) group-level bistability (red line, analytical solution for the population mean) underlie graded and all-or-none transitions, respectively. Parameters in (D) and (E) are identical with (B) and (C), respectively. (F) Phase diagram of CVρ/CVk. Solid, dashed and dotted black lines indicate analytically determined boundary AB/GB, AB/M, and GB/M, respectively. Yellow line is log10 CVρ/CVk = 0 determined numerically. × and + correspond to (D) and (E), respectively. (TIF) [file pcbi.1003110.s007.tif]

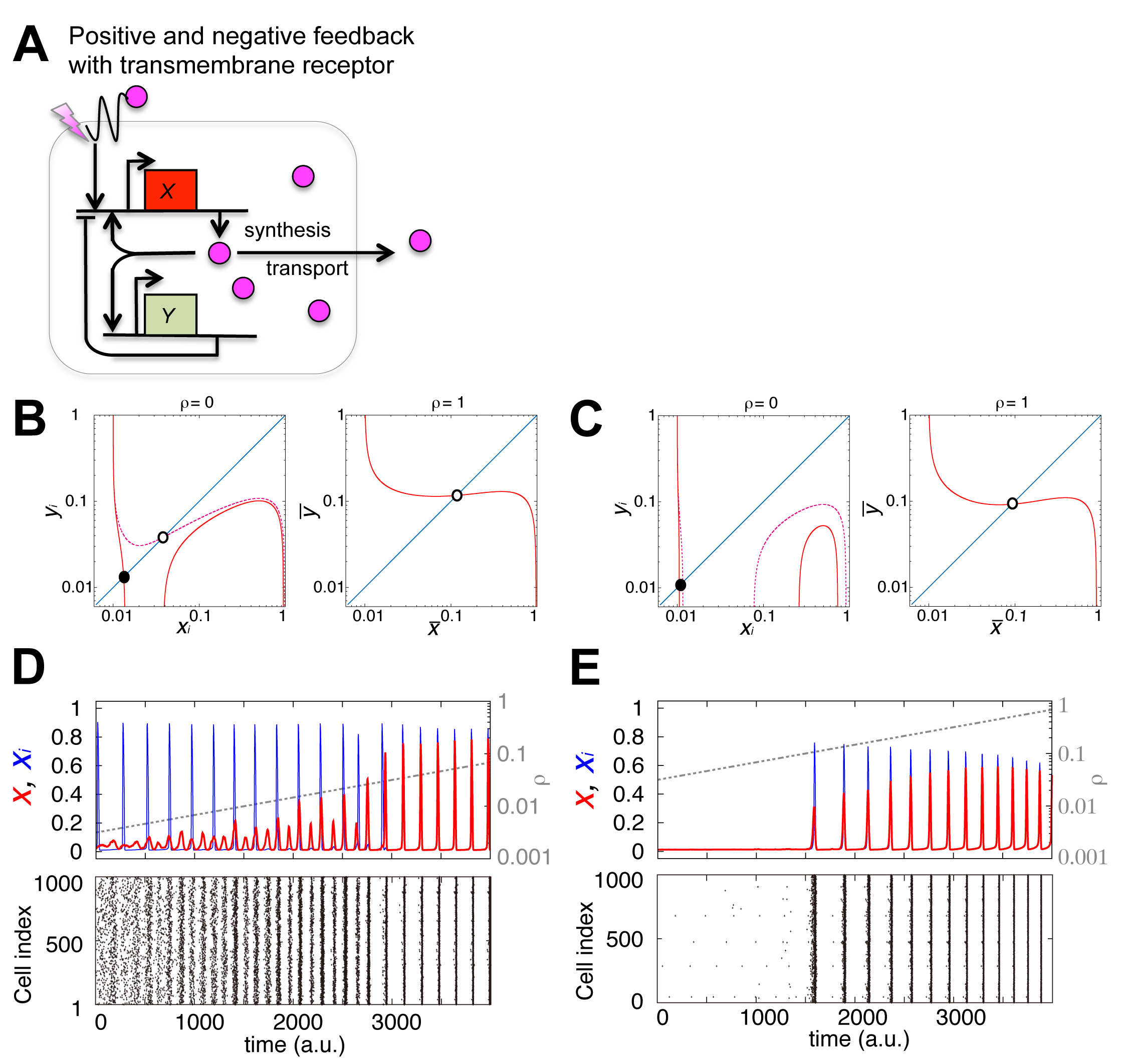

Supplement: Figure S8 — Dynamical quorum sensing transitions in a negative-feedback mediated trans-membrane receptor model. (A) Schematics of positive-and-negative feedback circuit with transmembrane receptor model (Eq. S1-34). (B)–(C) Nullclines for the isolated cell (Eq. S1-36; left panel) and the population (Eq. S1-37; right panel) predict transitions from quiescence to oscillations depending on cell density ρ. Similar to the direct import model (Eq. 4; Fig. S5C–D), at ρ = 0, cells are either excitatory or oscillatory depending on ki in case of ε = = 5.6 (B). Cells are always excitatory irrespective of ki in ε = = 2.8 (C). Solid and dotted lines at ρ = 0 indicate ki is 25% larger and smaller than (Eq. S1-24). α = 2.4, β = 10, and λ = 100 in (B) and (C). (D)–(E) Simulations of communicating cell populations demonstrate graded (D) and all-or-none (E) transitions during exponential increase in cell density ρ (grey line). The randomized parameter ki has a lognormal distribution with CVk = 0.25. ε = in (D) and (E) are the same with (B) and (C), respectively. λ, α, and β in (D) and (E) are also identical with (B) and (C). αy = 100. |ηi| = 0.0. (TIF) [file pcbi.1003110.s008.tif]

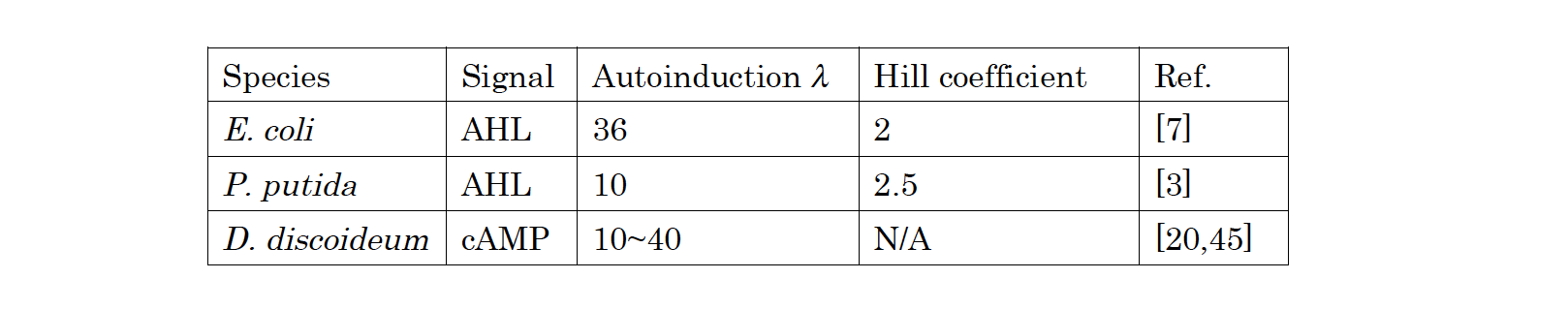

Supplement: Table S1 — Representative examples of autoinduction. (TIFF) [file pcbi.1003110.s009.tiff]

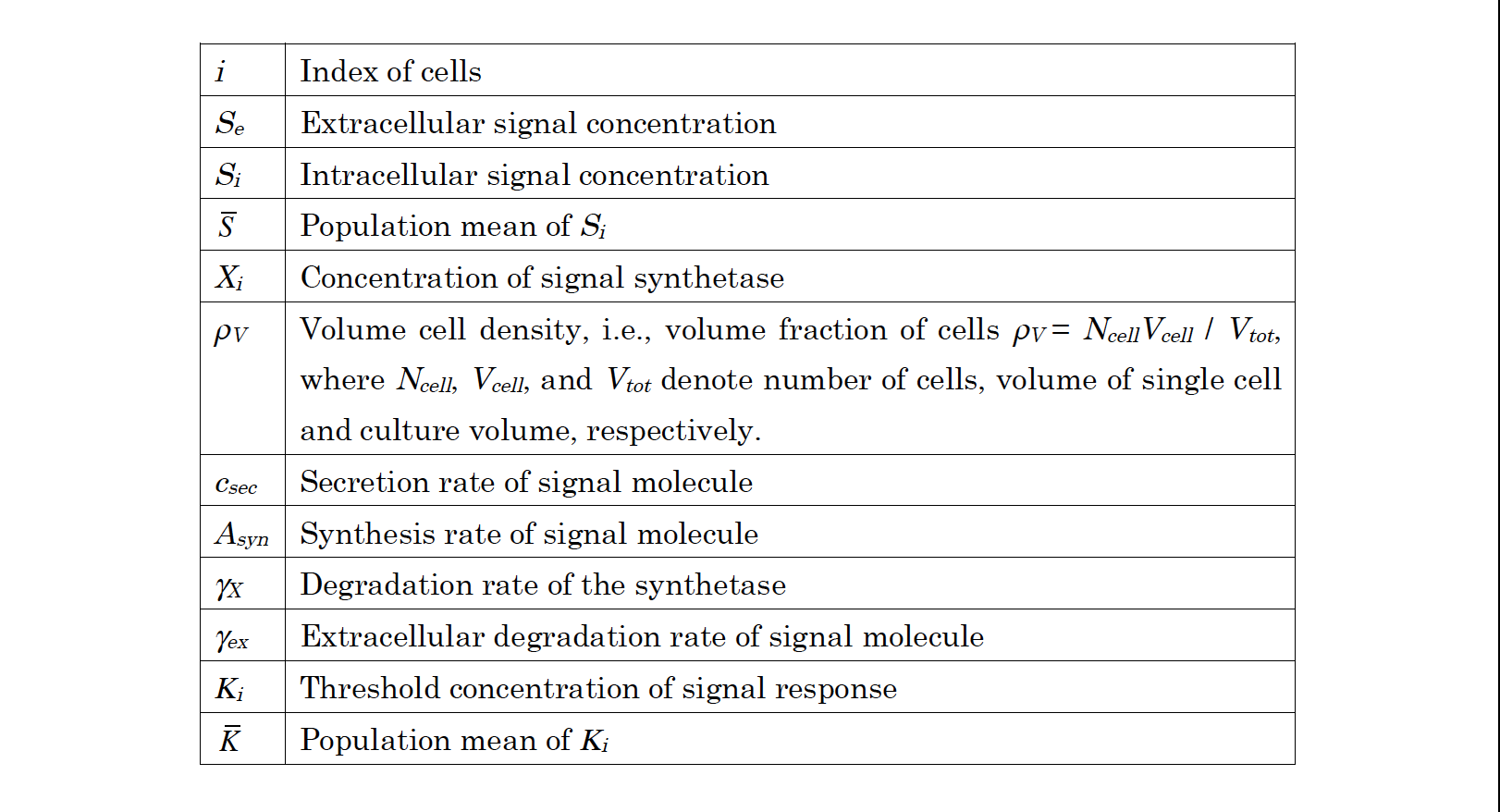

Supplement: Table S2 — List of variables and parameters of autoinduction kinetics Eq. S1-1 to Eq. S1-3. (TIFF) [file pcbi.1003110.s010.tiff]

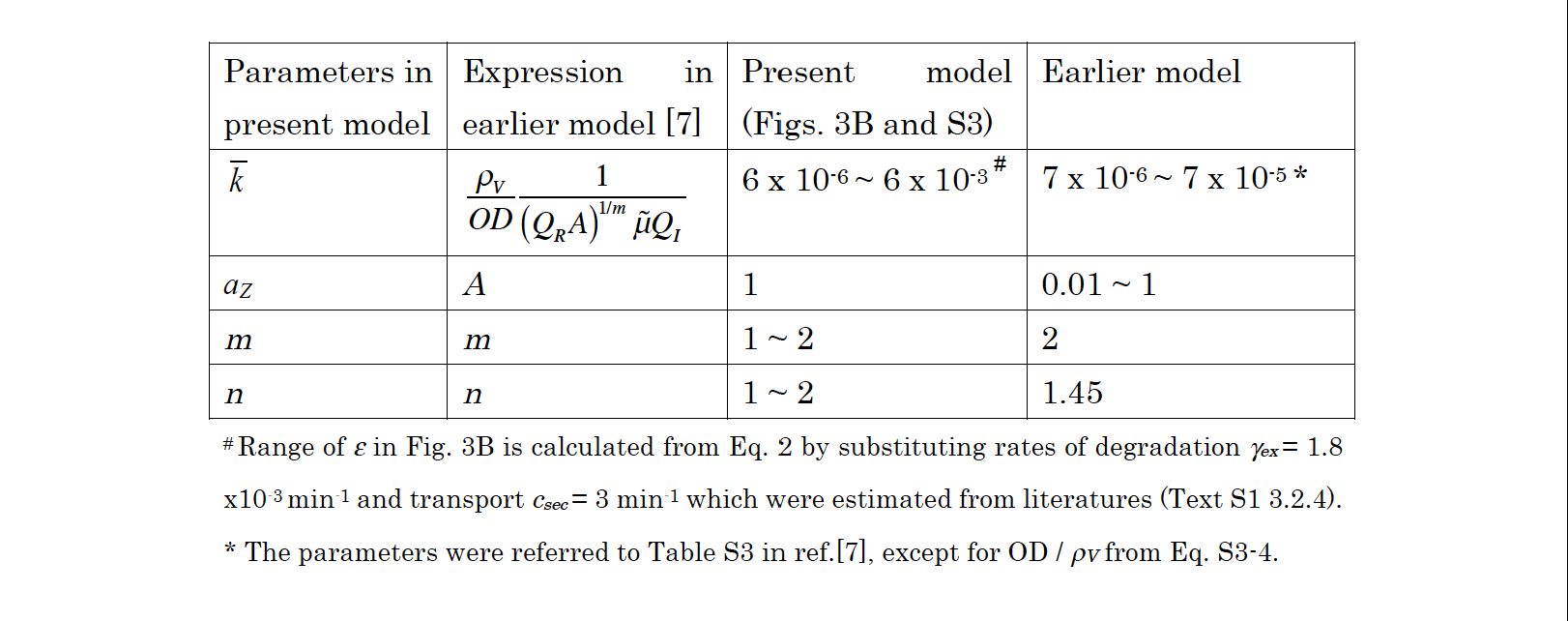

Supplement: Table S3 — Parameter values chosen in dual positive-feedback circuit Eq. 3 . (TIFF) [file pcbi.1003110.s011.tiff]

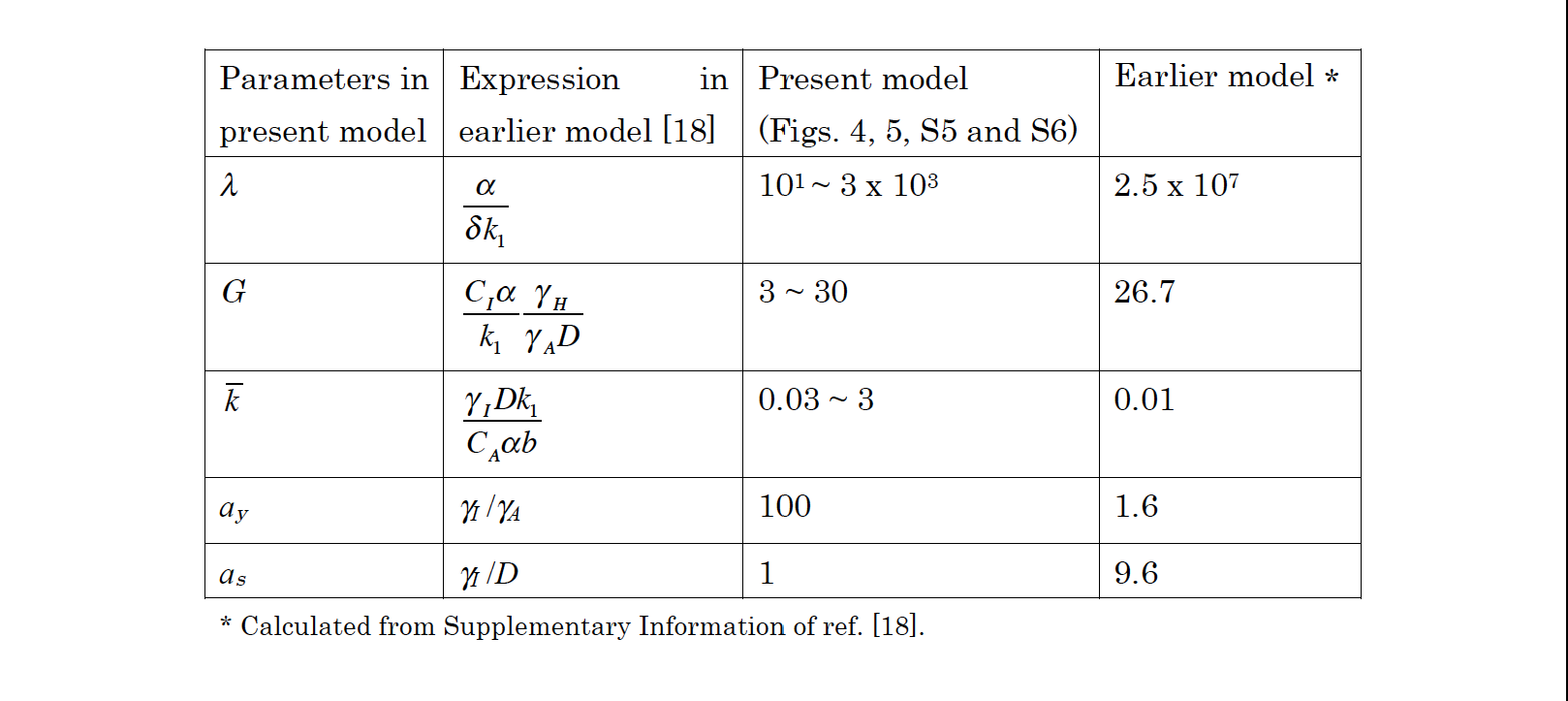

Supplement: Table S4 — Parameter values chosen in positive-and-negative feedback circuit Eq. 4 . (TIFF) [file pcbi.1003110.s012.tiff]

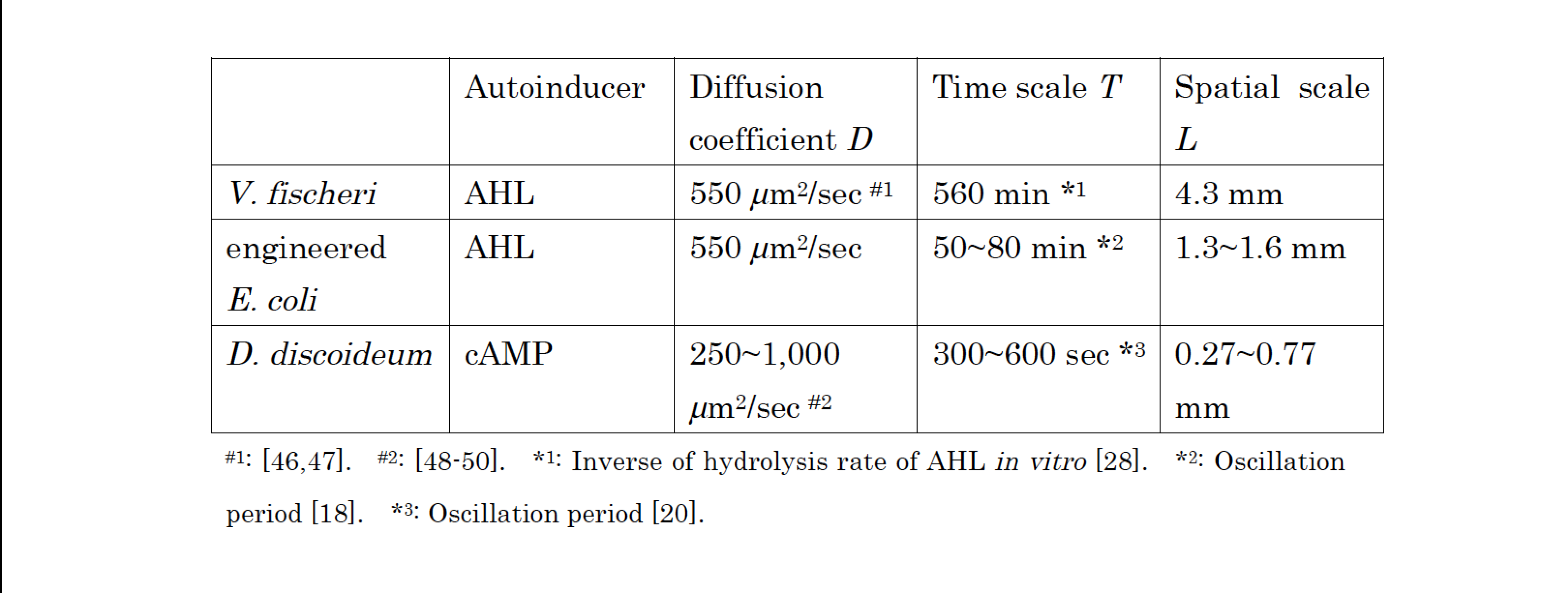

Supplement: Table S5 — Dimensional analysis of spatial scale of extracellular environment. (TIFF) [file pcbi.1003110.s013.tiff]

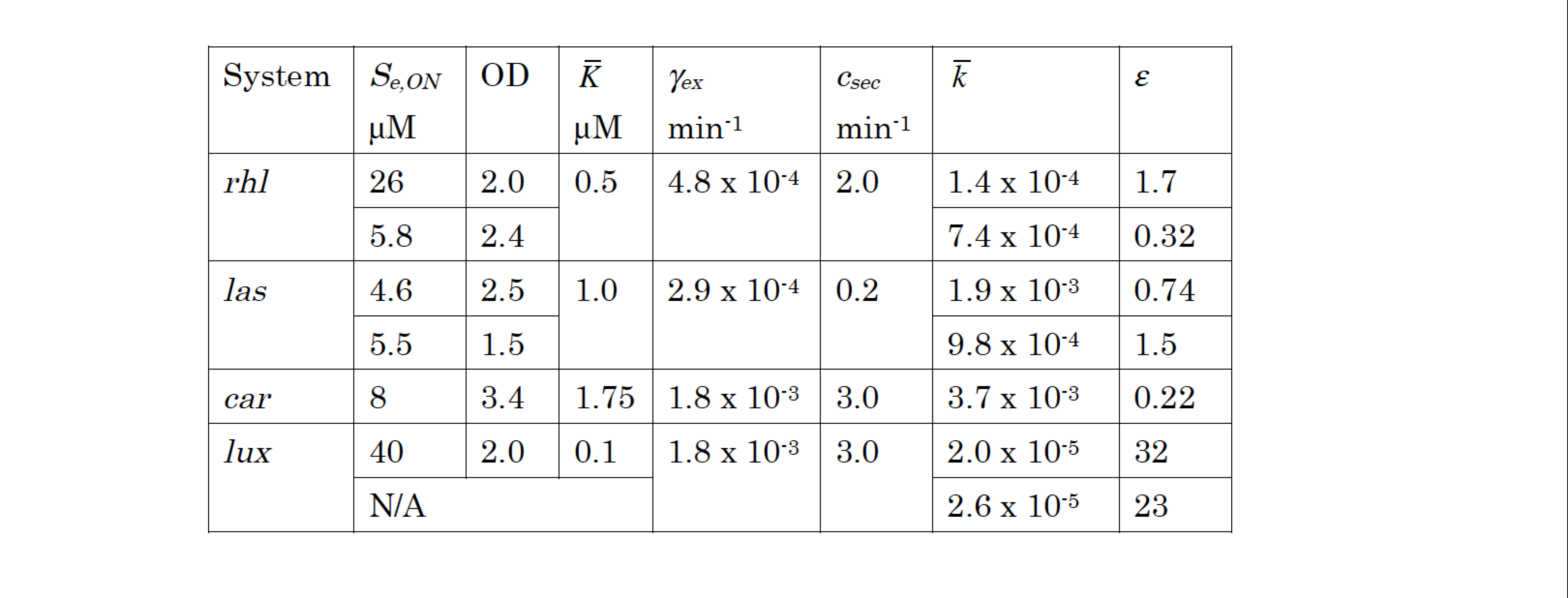

Supplement: Table S6 — Parameters estimated from literatures. (TIFF) [file pcbi.1003110.s014.tiff]
