# Supplementary material for: A Design Principle of Group-level Decision Making in Cell Populations
Source: PLoS Comput Biol. 2013 Jun 27;9(6):e1003110. doi: 10.1371/journal.pcbi.1003110 (PMC3694814; doi:10.1371/journal.pcbi.1003110)
Supplement: Text S1 — Supporting methods and results. (PDF) [file pcbi.1003110.s015.pdf]

Text S1. Supporting Methods and Results.

"A Design Principle of Group-level Decision Making in Cell Populations"

Koichi Fujimoto and Satoshi Sawai

1. **Derivation of mathematical models**
   - 1.1 **Autoinduction circuit with direct import**
   - 1.2 **Dual positive-feedback circuit**
   - 1.3 **Positive-and-negative-feedback circuit**
   - 1.4 **Autoinduction circuit with trans-membrane receptor**
   - 1.5 **Effect of intracellular degradation**
   - 1.6 **Effect of diffusion**
2. **Conditions for group-level / autonomous bistability**
   - 2.1 **Group-level bistability in the autoinduction circuit**
   - 2.2 **Autonomous bistability in the autoinduction circuit**
   - 2.3 **Phase boundary in the dual positive-feedback circuit**
   - 2.4 **Cell-autonomous excitability in the positive-and-negative-feedback circuit**
3. **Parameter estimation for bacterial quorum sensing**
   - 3.1 **Normalized threshold** $\bar{k}$
     - 3.1.1 **Estimation of** $\bar{k}$ **from extracellular signal concentration**
     - 3.1.2 **Estimation of** $\bar{k}$ **from gene expression level**
   - 3.2 **The order parameter** ε
     - 3.2.1 ***rhl* system**
     - 3.2.2 ***las* system**
     - 3.2.3 ***car* system**
     - 3.2.4 ***lux* system**

Supporting Table S1 – S6

Supporting Figure S1 – S8

References

1. Derivation of mathematical models

1.1 Autoinduction circuit with direct import

Let us first describe a model that we have employed to study cell-state transition mediated by autoinducing signal synthesis in a population consisted of N number of cells. The model assumes that each cell synthesizes autoinducer molecules that are passively transported in and out of cells and degraded in the extracellular space. The molecule is assumed to diffuse fast so that spatial heterogeneity such as concentration gradient is not taken into account (See Text S1 1.6 for the spatio-temporal scale where such "well-mixed" approximation is valid). The synthesis rate of autoinducer in the i-th cell (i = 1, 2, …, N) rapidly increases above a certain autoinducer concentration K_i. Kinetics of autoinducer synthesis, transport and degradataion in extra-cellular space (See Text S1 1.5 for effect of intra-cellular degradation) [1-3] are given by

$$\frac{dS_e}{d\tau} = \rho_V c_{\sec}\left(\overline{S} - S_e\right) - \gamma_{ex} S_e \quad \text{(S1-1)}$$

$$\frac{dS_i}{d\tau} = c_{\sec}\left(S_e - S_i\right) + A_{syn} X_i \quad \text{(S1-2)}$$

$$\frac{dX_i}{d\tau} = \left(V_{\max} - V_{\min}\right)\frac{S_i^2}{S_i^2 + K_i^2} + V_{\min} - \gamma_X X_i + \eta_{X,i} \quad \text{(S1-3)}$$

where S_e, S_i, and X_i are concentrations of extracellular and intracellular autoinducer and its synthetase in the ith cell. $\overline{S} = \frac{1}{N_{cell}}\sum_{j=1}^{N_{cell}} S_j$. V_{max} and V_{min} ($V_{min} < V_{max}$) denote the rate of enzyme synthesis at high and low concentration of intracellular autoinducer, respectively. According to change from V_{max} to V_{min}, the amount of autoinducer synthesized increases λ times, where

$$\lambda \equiv V_{\max} / V_{\min} \quad \text{(S1-4)}$$

represents the strength of autoinduction. Experimentally, the magnitude of λ has been measured (Table S1) and manipulated by an inducible promoter [4,5].

We incorporated cell-cell variation in the value of K_i and temporal fluctuation in the synthetase concentration X_i ($\eta_{X,i}$ in Eq. S1-3), to account for the heterogeneous response to exogenously applied autoinducer as observed for isolated *V. fischeri* cells [6]. Full definition of variables and parameters are listed in Table S2. The model is almost identical to those studied earlier [7] and accounts for the kinetics of bacterial autoinducers such as AHLs (*N*-Acyl homoserine lactones) that is passively transported into the cells (Fig. 1C). A slightly modified version of the model for a case where signal molecules bind to trans-membrane receptors will be derived in Text S1 1.4.

The sensitivity or the threshold concentration K_i for the switch response in Eq. S1-3 has been experimentally measured in isolated cells [6]. The Hill coefficient in Eq. S1-3 is higher than unity due to cooperative binding of signaling molecule to a transcriptional activator and DNA [7,8] as described in Test S1 1.2. To account for both group-level and autonomous bistability, the coefficient should be higher than unity [7]. Here we assume the Hill coefficient of 2. When the Hill coefficient is one, group-level bistability does not exist (Text S1 2.1) therefore the all-or-none transition cannot occur (Fig. S2C, grey points). On the other hand, when we take larger values of Hill coefficient, the order parameter ε at the boundary between autonomous bistability and monostability (Eq. S2-15) becomes larger, while the boundary between the group-level and autonomous bistability (Eq. S2-15) remains almost unchanged (data not shown). The increase of ε at the boundary between autonomous bistability and monostability with the Hill coefficient are also observed in the dual positive-feedback circuit (Eq. S2-23; Figs. 1D and 3B).

To clarify the relationship between the three variables S_e, S_i and X_i, we transformed Eqs. S1-1, S1-2 and S1-3 into an single equality. When we assume that intra-cellular and extra-cellular kinetics (Eqs. S1-1 and S1-2) equilibrate to a steady state rapidly compared to enzyme synthesis and

degradation (Eq. S1-3). Eqs. S1-1 and S1-2 become

$$S_e = \frac{\rho_V}{\rho_V + \frac{\gamma_{ex}}{c_{\sec}}} \bar{S} \qquad \text{(S1-5)},$$

$$S_i = S_e + \frac{A_{syn}}{c_{\sec}} X_i \qquad \text{(S1-6)}.$$

The first and second terms in the right-hand side of Eq. S1-6 represents autoinducer molecules imported from extracellular environment plus those that are synthesized and accumulated within the cell. By taking the ensemble average, Eq. S1-6 becomes

$$\frac{\frac{\gamma_{ex}}{c_{\sec}}}{\rho_V + \frac{\gamma_{ex}}{c_{\sec}}} \bar{S} = \frac{A_{syn}}{c_{\sec}} \bar{X} \qquad \text{(S1-7)}.$$

By substituting Eqs. S1-5 and S1-7 into Eq. S1-6, we obtain

$$S_i = \frac{A_{syn}}{\gamma_{ex}} \left(\rho_V \bar{X} + \frac{\gamma_{ex}}{c_{\sec}} X_i \right) \qquad \text{(S1-8)}.$$

where the first term is equal to S_e. The earlier model [7] studied a case $S_e = S_i$, i.e., $\gamma_{ex}/c_{sec} = 0$ which is oversimplification for the present purpose. For example, in *P. aeruginosa,* S_i is more than three times larger than S_e [9]. Thus in this study, the intracellular autoinducer concentration is represented by Eq. S1-8 for a more general case $S_e \leq S_i$. †

---

† The large difference between S_e and S_i can be confirmed by the coefficients in the second and the first term of Eq. S1-8, i.e., $(S_i - S_e)/S_e = \gamma_{ex}/c_{sec}/\rho_V$, when $X_i = \bar{X}$. ρ_V near the threshold can be estimated from OD in bulk experiments (Eq. S3-4; Table S6). As will be shown in Text S1 2.2, our model predicts that the autonomous bistability occurs when $\gamma_{ex}/c_{sec}/\rho_V$ is smaller than 1 but non-zero. For the operons in Table S6, $\gamma_{ex}/c_{sec}/\rho_V$ is 0.02 to 0.26 which is indeed small but not zero meaning that the autonomous bistability can occur.

Finally, based on the expression of S_i (Eq. S1-8), we derive a single variable differential equation Eq. 1 and a unique parameter ε that determines the nature of the switch. By substituting Eq. S1-8 into Eq. S1-3, the model (Eqs. S1-1 to S1-3) is reduced to a single variable

$$\frac{dX_i}{d\tau} = \left(V_{max} - V_{\min}\right) \frac{\left(\frac{A_{syn}}{\gamma_{ex}}\left(\rho_V \bar{X} + \frac{\gamma_{ex}}{c_{\sec}} X_i\right)\right)^2}{\left(\frac{A_{syn}}{\gamma_{ex}}\left(\rho_V \bar{X} + \frac{\gamma_{ex}}{c_{\sec}} X_i\right)\right)^2 + K_i^2} + V_{\min} - \gamma_X X_i + \eta_{X,i}.$$

By rescaling variables X_i , S_i, $\eta_{X,i}$ and τ by

$$\begin{cases} x_i = \dfrac{\gamma_X}{V_{\max}} X_i, \\ s_i = \dfrac{\gamma_{ex}}{A_{syn}} \dfrac{\gamma_X}{V_{\max}} S_i, \\ \eta_i = \dfrac{\eta_{X,i}}{V_{\max}}, \\ t = \gamma_X \tau \end{cases} \qquad \text{(S1-9)},$$

and by using normalized parameter

$$k_i = \frac{\gamma_{ex}\gamma_X}{A_{syn} V_{\max}} K_i \qquad \text{(S1-10)},$$

we obtain Eq. 1 in the main text

$$\begin{cases} \dfrac{dx_i}{dt} = \left(1 - \lambda^{-1}\right) \dfrac{s_i^2}{s_i^2 + k_i^2} + \lambda^{-1} - x_i + \eta_i \\ s_i = \rho_V \bar{x} + \dfrac{\gamma_{ex}}{c_{\sec}} x_i \end{cases}.$$

where η_i is white Gaussian noise in the chemical Langevin equation [10]. At the steady state, the variance of the noise is given by [11,12]

$$|\eta_i|^2 = \left(\left(1-\lambda^{-1}\right) \frac{s_i^2}{s_i^2 + k_i^2} + \lambda^{-1} + x_i \right) \frac{1}{\gamma_X N_{ON}} = \frac{2x_i}{\gamma_X N_{ON}} \quad \text{(S1-11)},$$

where N_{on} denotes the number of synthetase molecules in the ON-state cell (Eq. S1-3) given by

$$N_{ON} = X_{ON} V_{cell},\ X_{ON} = \frac{V_{\max}}{\gamma_X} \quad \text{(S1-12)}.$$

Using the original parameters, the order parameter ε (Eq. 2; Fig. 3) is given by

$$\varepsilon \equiv \frac{\gamma_{ex}}{c_{\sec}\bar{k}} = \frac{A_{syn}V_{\max}}{\gamma_X} \frac{1}{c_{\sec}\bar{K}} \quad \text{(S1-13)}.$$

where A_{syn} and γ_X, are synthesis rate of autoinducer S_i and degradation rate of synthetase X_i respectively, and $\bar{k} \equiv \frac{1}{N_{cell}} \sum_i^{N_{cell}} k_i$ and $\bar{K} \equiv \frac{1}{N_{cell}} \sum_i^{N_{cell}} K_i$. By substituting X_{ON} (Eq. S1-12) into Eq. S1-13, we obtain Eq. 2 by

$$\varepsilon = \frac{A_{syn}X_{ON}}{c_{\sec}\bar{K}} \quad \text{(S1-14)}$$

where $A_{syn}X_{on}/c_{sec}$ denotes the autoinducer concentration at the maximum synthesis activity (Eqs. S1-6 and S1-8). The concentration is equal to the threshold $\bar{K}$ when $\varepsilon = 1$, which roughly corresponds to the boundary between the graded and all-or-none transitions (Text S1 2.2).

1.2 Dual positive-feedback circuit

Recent experiments have shown that when an additional positive feedback loop is introduced into the autoinduction circuit (Fig. 1C and Eq. 1; Fig. 1D), autonomous bistability becomes more robust to parameter variation [13].

To analyze such a circuit, we extend the simple autoinduction circuit (Eqs. S1-1 to S1-3), and derive the order parameter ε and show that this is identical with the that obtained for the simple autoinduction circuit (Eq. S1-13).

An additional feedback loop is typically found in bacterial quorum sensing systems such as complex formation of a transcriptional activator molecule LuxR with autoinducer molecules in *V. fischeri* [14] (Table 1). The promoter-complex formation induces expression of genes that encode the activator itself and the autoinducer synthase. To describe this, Eq. S1-3 is extended as follows:

$$\begin{cases} \dfrac{dX_i}{d\tau} = \left(V_{\max} - V_{\min}\right)\dfrac{\left(Z_i S_i^m\right)^n}{\left(Z_i S_i^m\right)^n + \left(K_i^m\right)^n} + V_{\min} - \gamma_X X_i + \eta_{X,i} \\ \dfrac{dZ_i}{d\tau} = v_z\left(\left(V_{\max} - V_{\min}\right)\dfrac{\left(Z_i S_i^m\right)^n}{\left(Z_i S_i^m\right)^n + \left(K_i^m\right)^n} + V_{\min}\right) - \gamma_Z Z_i \end{cases} \quad \text{(S1-15)},$$

where Z, m and n denote concentration of the activator, cooperativity of S-Z and Z-promoter binding, respectively. The model closely follows that proposed recently by Thattai and his colleagues [7], with exception of a minor change in the expression of S_i (Eq. S1-8). The parameter values were determined from earlier experimental studies (m = 2 and n = 1.45 in the *luxI* and *luxR* circuit [7,15]) (Table S3). v_Z represents relative difference of synthesis rate between Z and X. In addition to normalization of X, S, τ as in the simple autoinduction circuit (Eq. S1-9), the variable Z and parameter K_i are normalized as

$$
\begin{cases}
z_i = \dfrac{\gamma_z}{v_z V_{\max}} Z_i \\
k_i = \dfrac{\gamma_{ex}\gamma_X}{A_{syn} V_{\max} a_z^{\frac{1}{m}}} K_i \\
a_z = \dfrac{v_Z \gamma_X}{\gamma_Z}
\end{cases}
\qquad \text{(S1-16).}
$$

We obtain expression for Eq. 3,

$$
\begin{cases}
\dfrac{dx_i}{dt} = \left(1-\lambda^{-1}\right)\dfrac{z_i^n \cdot s_i^{m\cdot n}}{z_i^n \cdot s_i^{m\cdot n} + k_i^{m\cdot n}} + \lambda^{-1} - x_i + \eta_i \\
a_z \dfrac{dz_i}{dt} = \left(1-\lambda^{-1}\right)\dfrac{z_i^n \cdot s_i^{m\cdot n}}{z_i^n \cdot s_i^{m\cdot n} + k_i^{m\cdot n}} + \lambda^{-1} - z_i \\
s_i = \rho_V \bar{x} + \dfrac{\gamma_{ex}}{c_{\sec}} x_i
\end{cases}
$$

At a steady state, we see that

$$
\begin{cases}
z_i = x_i, \\
Z_i = a_z X_i
\end{cases}
\qquad \text{(S1-17).}
$$

The order parameter ε (Eq. 2; Fig. 3B) is given by

$$
\varepsilon \equiv \frac{\gamma_{ex}}{c_{\sec}\bar{k}} = \frac{A_{syn}V_{\max}}{\gamma_X}\frac{a_z^{\frac{1}{m}}}{c_{\sec}\bar{K}} \qquad \text{(S1-18),}
$$

where bars indicate population mean. By assuming $a_z = 1$, i.e., $Z_i = X_i$ at the steady state (Eq. S1-17), one can see that Eq. S1-18 reduced to Eq. S1-13. Notations for the model parameters are summarized and listed in Table S3.

1.3 Positive-and-negative-feedback circuit

As a natural extension of quorum sensing, we introduced a negative feedback loop into the simple autoinducing gene circuit (Fig. 1C and Eq. 1; Fig. 1E; Table 2) to study the so-called dynamical quorum sensing transition [16-21]. We adopt a model recently proposed by Danino and colleague to describe the synthetic oscillatory circuit [18]. Here, autoinducer AHL directly activates expression of *aiiA* as well as its own synthetase *luxI*. Gene *aiiA* encodes lactonase AiiA that degrades AHL. Following the notation in ref.[18], the kinetics are given by

$$\begin{cases} \dfrac{dX_i}{d\tau} = C_I \dfrac{\delta + \alpha S_i^2}{1 + k_1 S_i^2} - \gamma_I X_i + \eta_{X,i} \\ \dfrac{dY_i}{d\tau} = C_A \dfrac{\delta + \alpha S_i^2}{1 + k_1 S_i^2} - \gamma_A Y_i \\ \dfrac{dS_i}{d\tau} = bX_i - \gamma_H Y_i S_i + D\left(S_e - S_i\right) \\ \dfrac{dS_e}{d\tau} = D\rho_V\left(\bar{S} - S_e\right) - \mu S_e \end{cases} \qquad \text{(S1-19)},$$

where X_i and Y_i denote LuxI and AiiA concentration, respectively. For simplicity, the time delay from gene regulation to *luxI* and *aiiA* were assumed to be negligible, and Michaelis-Menten kinetics of degradation and synthesis of autoinducer was approximated by first-order kinetics.

To clarify the relationship between Eq. S1-19 and the simple autoinduction circuit (Eq. 1), we normalize the variable X_i, Y_i, and S_i as in Eq. S1-9, and derive the parameter ε and show that this again yields identical formula to that we obtained for the simple autoinduction circuit (Eq. S1-13). First, by assuming quasi-steady state of extracellular AHL kinetics and by replacing the parameter notation in line with the present autoinduction model (Eqs. S1-1 to S1-3), Eq. S1-19 become

$$\begin{cases} \dfrac{dX_i}{d\tau} = \left(V_{\max} - V_{\min}\right)\dfrac{S_i^2}{S_i^2 + K_i^2} + V_{\min} - \gamma_I X_i + \eta_{X,i} \\ \dfrac{dY_i}{d\tau} = \dfrac{C_A}{C_I}\left(\left(V_{\max} - V_{\min}\right)\dfrac{S_i^2}{S_i^2 + K_i^2} + V_{\min}\right) - \gamma_A Y_i \\ \dfrac{dS_i}{d\tau} = A_{syn} X_i - \gamma_H Y_i S_i + c_{\sec}\left(\rho \overline{S} - S_i\right) \end{cases} \qquad \text{(S1-20),}$$

where

$$\begin{cases} V_{\min} = C_A \delta \\ V_{\max} = \dfrac{C_A \alpha}{k_1} \\ K_i = \dfrac{1}{\sqrt{k_1}} \\ \rho = \dfrac{\rho_V}{\rho_V + \dfrac{\gamma_{ex}}{c_{\sec}}} = \dfrac{\rho_V}{\rho_V + \dfrac{\mu}{D}} \end{cases} \qquad \text{(S1-21),}$$

As in the simple autoinduction circuit (Eq. S1-9), by normalizing the variables as

$$\begin{cases} x_i = \dfrac{\gamma_I}{V_{\max}} X_i \\ y_i = \dfrac{C_I}{C_A}\dfrac{\gamma_A}{V_{\max}} Y_i \\ s_i = \dfrac{c_{\sec}\gamma_I}{A_{syn} V_{\max}} S_i \\ t = \gamma_I \tau \end{cases} \qquad \text{(S1-22),}$$

we arrive at Eq. 4 in the main text,

$$\begin{cases} \dfrac{dx_i}{dt} = \left(1-\lambda^{-1}\right)\dfrac{s_i^2}{s_i^2+k_i^2} + \lambda^{-1} - x_i + \eta_i \\ a_y \dfrac{dy_i}{dt} = \left(1-\lambda^{-1}\right)\dfrac{s_i^2}{s_i^2+k_i^2} + \lambda^{-1} - y_i \\ a_s \dfrac{ds_i}{dt} = x_i - g y_i s_i + \rho \overline{s} - s_i \end{cases}$$

where

$$\begin{cases} k_i = \dfrac{c_{\sec}\gamma_I}{A_{syn}V_{\max}} K_i = \dfrac{\gamma_I D k_1}{C_A \alpha b} \\ g = \dfrac{C_I}{C_A}\dfrac{V_{\max}}{c_{\sec}\gamma_A}\gamma_H = \dfrac{C_I\alpha}{k_1}\dfrac{\gamma_H}{\gamma_A D} \\ a_y = \dfrac{\gamma_I}{\gamma_A} \\ a_s = \dfrac{\gamma_I}{c_{\sec}} \end{cases} \qquad \text{(S1-23).}$$

Comparison of Eq. S1-23 and Eq. S1-13 indicates that the mean threshold $\overline{k}$ corresponds to inverse of the order parameter ε in the simple autoinduction circuit

$$\overline{k} = \frac{1}{N_{cell}}\sum_i^{N_{cell}} k_i = \frac{c_{\sec}\gamma_I}{A_{syn}V_{\max}}\overline{K} = \varepsilon^{-1} \qquad \text{(S1-24),}$$

since γ_I corresponds to γ_X in Eq. S1-3. Table S4 shows comparison of these parameters to those in the earlier model (Eq. S1-19) [18].

Finally, to analytically study the group-level transition, we analyze the nullclines for an isolated cell and the population. First, we consider the isolated condition, $\rho = 0$. At the steady state, Eq. 4 becomes

$$\begin{cases} \dfrac{dx_i}{dt} = \left(1-\lambda^{-1}\right)\dfrac{s_i^2}{s_i^2+k_i^2} + \lambda^{-1} - x_i = 0 \\ a_y \dfrac{dy_i}{dt} = \left(1-\lambda^{-1}\right)\dfrac{s_i^2}{s_i^2+k_i^2} + \lambda^{-1} - y_i = 0 \\ a_s \dfrac{ds_i}{dt} = x_i - gy_i \cdot s_i - s_i = 0 \end{cases} \quad \text{(S1-25).}$$

By substituting the third equation into the first and second equations, we obtain nullclines for the isolated condition ($\rho = 0$ in Fig. S5C-D). They are

$$\begin{cases} y_i = \dfrac{1}{g}\left(-1 + \dfrac{x_i}{k_i}\sqrt{\dfrac{1-x_i}{x_i-\lambda^{-1}}}\right) \\ x_i = \left(gy_i + 1\right)k_i\sqrt{\dfrac{y_i-\lambda^{-1}}{1-y_i}} \end{cases} \quad \text{(S1-26).}$$

Next, assuming that there is a group-level all-or-none response, we can take $x_i = \overline{x},\ y_i = \overline{y},\ s_i = \overline{s}$ for all i at the steady state. Eq. 4 is rewritten as

$$\begin{cases} \dfrac{d\overline{x}}{dt} = \left(1-V_{\max/\min}^{-1}\right)\dfrac{\overline{s}^2}{\overline{s}^2+\overline{k}^2} + \lambda^{-1} - \overline{x} = 0 \\ a_y \dfrac{d\overline{y}}{dt} = \left(1-V_{\max/\min}^{-1}\right)\dfrac{\overline{s}^2}{\overline{s}^2+\overline{k}^2} + \lambda^{-1} - \overline{y} = 0 \\ a_s \dfrac{d\overline{s}}{dt} = \overline{x} - g\overline{y} \cdot \overline{s} + \rho\overline{s} - \overline{s} = 0 \end{cases} \quad \text{(S1-27),}$$

where bars indicate the population mean. By substituting the third equation and Eq S1-24 into the first and second equations, we obtain nullclines for the homogeneous population (e.g., $\rho = 1$ in Fig. S5C-D). They are

$$\begin{cases} \bar{y} = \frac{1}{g}\left(\rho - 1 + \bar{x}\varepsilon\sqrt{\frac{1-\bar{x}}{\bar{x}-\lambda^{-1}}} \right) \\ \bar{x} = \left(g\bar{y} - \rho + 1 \right)\varepsilon^{-1}\sqrt{\frac{\bar{y}-\lambda^{-1}}{1-\bar{y}}} \end{cases} \quad \text{(S1-28).}$$

Autonomous oscillations (Figs. 4A and S5A) can be distinguished from the autonomous excitation [22,23] by looking at two properties of the nullclines in the isolated condition (Eq. S1-26). One is the slope of the nullclines at their intersection. The other is whether or not x at the intersection is larger than x at the local minimum and maximum of the nullcline $dx_i/dt = 0$. When the value of ε is high, cells with $k_i < \bar{k} = \varepsilon^{-1}$ (Eq. S1-24) exhibit an oscillatory instability (Fig. S5C, $\rho = 0$, dotted lines). In the oscillatory cells, the instability is indicated by a fixed point, i.e., an intersection of the nullclines, where the gradient of $dy_i/dt = 0$ is steeper than that of $dx_i/dt = 0$ (Fig. S5C, $\rho = 0$, open circle at intersection of the dotted lines). Irrespective of the initial conditions and the magnitude of η_i (Eq. 4), the trajectory in the state space (x_i, y_i) converges to a limit cycle, whose trajectory is roughly bounded by the local minimum and maximum of the nullcline $dx_i/dt = 0$. At the same time, the other cells having $k_i > \bar{k}$ are excitable (Fig. S5C, $\rho = 0$, solid lines). In the excitable cells, a fixed point (closed circle in Fig. S5C, $\rho = 0$) which is located on the left of local minimum of the nullcline $dx_i/dt = 0$ (at $x_i \sim 0.001$ in Fig. S5C, $\rho =$ 0) indicates stability. When intrinsic noise or extrinsic perturbation dislocates the state (x_i, y_i) on right of the local minimum ($x_i > 0.01$ in Fig. S5C, $\rho = 0$), the state transiently escapes from the stable fixed point and reaches a local maximum of the nullcline $dx_i/dt = 0$ (Fig. S5C, at $x_i \sim 0.5$), before returning to the stable fixed point. In contrast, at low ε, the system exhibits excitability irrespective of the magnitude of k_i, since the stable fixed point is lower than the local minimum $dx_i/dt = 0$ (Fig. S5D, $\rho = 0$, solid and dashed lines), as seen for high ε.

Similarly, the nullclines obtained at the level of population description (Eq. S1-28) allow us to identify the type of bifurcation that takes place when the group of cells switches from excitation to oscillations above a certain cell density. A pair of stable and unstable fixed points (closed and left open circles in $\rho = 0$ in Fig. S5C) disappears so that a limit cycle around the other unstable fixed point (right open circle at $\rho = 0$ in Fig. S5C; $\rho = 1$ in Fig. S5C-D) emerges. The nullclines in the isolated cell and the cell population together capture the essence of the graded transition at high ε. At low density, there is a sub-population of cells that are autonomously oscillatory. As density is increased, the remaining population undergoes a saddle-node bifurcation so that resulting transition at the population-level appears to occur gradually.

1.4 Autoinduction circuit with transmembrane receptor

In many cases, especially in eukaryotic cells, the signaling molecules do not pass freely thoroughly the membrane nor act directly in the autoregulatory loop. The autoinducing molecule binds to a transmembrane receptor to invoke a series of second-messenger responses that lead to further synthesis and secretion of autoinducing molecules. After adiabatic elimination of the signal transduction kinetics which usually takes place at a much faster time scale compared to autoinducer synthesis, kinetics of autoinducer can be simplified so that they appear, at the level of mathematical description, to directly obey the extracellular signal concentration S_e. Instead of Eq. S1-3, the kinetics of synthetase concentration is represented by

$$\frac{dX_i}{d\tau} = \left(V_{\max} - V_{\min}\right)\frac{S_e^2}{S_e^2 + K_i^2} + V_{\min} - \gamma_X X_i \qquad \text{(S1-29)}.$$

From steady state approximation of Eqs. S1-1 and S1-2 and by rescaling Eq. S1-10, Eq. S1-29 becomes

$$\frac{dx_i}{dt} = \left(1-\lambda^{-1}\right)\frac{\left(\rho_V \bar{x}\right)^2}{\left(\rho_V \bar{x}\right)^2 + k_i^2} + \lambda^{-1} - x_i \qquad \text{(S1-30)},$$

where k_i is given by Eq. S1-10. Eq. S1-30 becomes Eq. 1 when $\gamma_{ex} / c_{sec} \sim 0$, i.e., export rate c_{sec} is much larger than extracellular degradation rate γ_{ex}. To account for autonomous bistablity, an additional intracellular positive feedback such as one described in Eq. 3 is introduced into Eq. S1-30 so that

$$\frac{dx_i}{dt} = \left(1-\lambda^{-1}\right)\frac{x_i^2\left(\rho_V \bar{x}\right)^2}{x_i^2\left(\rho_V \bar{x}\right)^2 + k_i^2} + \lambda^{-1} - x_i \qquad \text{(S1-31)}.$$

However, under the isolated condition ($\rho_V = 0$) we obtain $\frac{dx_i}{dt} = 0 + \lambda^{-1} - x_i$, so that the autonomous bistability cannot occur. In other words, cooperative regulation between extracellular and intracellular feedback, quite naturally, must form an OR gate instead of an AND gate [24]. We thus obtain

$$\frac{dx_i}{dt} = \left(1-\lambda^{-1}\right)\frac{\left(\rho_V \bar{x}\right)^2 + \alpha^2 x_i^2}{\left(\rho_V \bar{x}\right)^2 + \alpha^2 x_i^2 + k_i^2} + \lambda^{-1} - x_i + \eta_i \qquad \text{(S1-32)},$$

where α represents strength of the intracellular feedback (Fig. S7A). Eq. S1-32 exhibits both cell and population bistability depending on ε which is

$$\varepsilon \equiv \frac{\alpha}{\bar{k}} = \frac{\alpha A_{syn} V_{\max}}{\gamma_{ex}\gamma_X \bar{K}} \qquad \text{(S1-33)},$$

where the second equality is derived from Eq. S1-13. The phase boundary of graded and all-or-none transitions underlying autonomous and group-level bistability, respectively, is identical with the simple autoinduction circuit Eq. S2-15 (Fig. S7B-F).

As with the direct import model, we extend our study on the role of parameter ε to oscillatory transitions. By introducing a negative feedback into Eq. S1-32 (Fig. S8A), we obtain

$$\begin{cases} \dfrac{dx_i}{dt} = \left(1-\lambda^{-1}\right)\dfrac{\rho_V \bar{x} + \alpha^2 x_i^2}{\rho_V \bar{x} + \alpha^2 x_i^2 + \beta^2 y_i^2 + k_i^2} + \lambda^{-1} - x_i \\ a_y \dfrac{dy_i}{dt} = x_i - y_i \end{cases} \quad \text{(S1-34).}$$

where a_y and β denote time scale of y and strength of the negative feedback, respectively. For simplicity, we assume synthesis of y_i to follow first order kinetics. Following the same approach taken for the direct import model (Eq. 4; Eq. S1-25 to S1-28), we derive the nullclines for the isolated cells and for the population. When cells are isolated ($\rho_V = 0$) and at the steady state, Eq. S1-34 becomes

$$\begin{cases} \dfrac{dx_i}{dt} = \left(1-\lambda^{-1}\right)\dfrac{\alpha^2 x_i^2}{\alpha^2 x_i^2 + \beta^2 y_i^2 + k_i^2} + \lambda^{-1} - x_i = 0 \\ a_y \dfrac{dy_i}{dt} = x_i - y_i = 0 \end{cases} \quad \text{(S1-35).}$$

By rewriting Eq. S1-35, we obtain nullclines for the isolated condition ($\rho_V = 0$ in Fig. S8B-C)

$$\begin{cases} y_i = \dfrac{1}{\beta}\sqrt{\dfrac{1-x_i}{x_i-\lambda^{-1}}\alpha^2 x_i^2 - k_i^2} \\ y_i = x_i \end{cases} \quad \text{(S1-36).}$$

At the population-level description, when the concentrations of all cells are identical, i.e., $x_i = \bar{x}$ and $y_i = \bar{y}$, we obtain nullclines

$$\begin{cases} \bar{y} = \dfrac{1}{\beta}\sqrt{\dfrac{1-\bar{x}}{\bar{x}-\lambda^{-1}}\left(\rho_V \bar{x} + \alpha^2 \bar{x}^2\right) - \bar{k}^2} \\ \bar{y} = \bar{x} \end{cases} \quad \text{(S1-37).}$$

The nullclines for the isolated condition (Eq. S1-36) and for the homogeneous population (Eq. S1-37) are shown in Fig. S8B-C. We see that, the slope and the shape of the nullclines that account for excitation and oscillations exhibit the same dependence on the order parameter ε and the cell density ρ_V as described above for the direct import model (Eq. 4; Fig. S5C-D). In the isolated condition ($\rho_V = 0$), at high ε, a fraction of the cells are oscillatory having an unstable fixed point but no stable fixed point (dotted line in Fig. S8B, $\rho_V = 0$). The remainder of the cells in a stable fixed point are however excitable; i.e. suprathreshold external perturbation or intrinsic noise would transiently evoke a trajectory of large excursion towards the local maximum of the nullcline $dx_i/dt = 0$ (at $x_i \sim 0.5$, $y_i \sim 0.1$ in Fig. S8B, $\rho_V = 0$) before returning to the stable fixed point (closed circle in Fig. S8B, $\rho_V = 0$). In high density populations, the stable fixed point and an unstable fixed point coalesce and disappear via saddle node bifurcation so that oscillatory state emerges around the other unstable fixed point (Fig. S8B, $\rho_V = 1$). Because this transition happens gradually as cell density increases, we have a graded transition at the population level. In contrast, at low ε, the individual cells are all excitable at first (dotted and solid lines in Fig. S8C, $\rho_V = 0$), before they bifurcate to the oscillatory state simultaneously at high density (Fig. S8C, $\rho_V = 1$). These properties of nullclines at high and low ε are consistent with behaviors observed in numerical simulations (Fig. S8D-E).

1.5 Effect of intracellular degradation

When intracellular degradation of the autoinducer is incorporated into the basic model (Eq. S1-1 ~ S1-3; Fig. 1C), Eq. S1-2 is replaced by

$$\frac{dS_i}{d\tau} = c_{\sec}\left(S_e - S_i\right) + A_{syn}X_i - \gamma_{in}S_i \qquad \text{(S1-38)},$$

where γ_{in} denotes rate of the intracellular degradation. Accordingly, the

intracellular concentration of autoinducer s_i (Eq. 1) and the order parameter ε (Eq. 2) are rewritten as

$$\begin{cases} s_i = \dfrac{1}{\left(1+\dfrac{\gamma_{in}}{c_{\sec}}\right)} \left(\dfrac{1}{1+\dfrac{\gamma_{in}}{\gamma_{ex}}\left(\rho_V + \dfrac{\gamma_{ex}}{c_{\sec}}\right)} \rho_V \bar{x} + \dfrac{\gamma_{ex}}{c_{\sec}} x_i \right), \\ \varepsilon = \dfrac{1}{\left(1+\dfrac{\gamma_{in}}{c_{\sec}}\right)} \dfrac{\gamma_{ex}}{c_{\sec}\bar{k}} \end{cases} \quad \text{(S1-39).}$$

By comparing Eq. S1-39 with Eqs. 1 and 2, one sees that as long as γ_{in} is much lower than c_{sec} and γ_{ex}, the rate of intracellular degradation has little influence on the value of ε nor does it change the steady state level of s_i. This is supported by estimating values of γ_{in}, c_{sec} and γ_{ex}. γ_{in} can be estimated from the effective dilution rate of intracellular molecules due to cell growth, e.g., order of 10^{-2} min^{-1}, which is much lower than c_{sec} = 0.2 ~ 3.0 min^{-1} (estimated in Table S6). On the other hand, γ_{in} is considerably higher than γ_{ex} = 4.8 x 10^{-4} ~ 1.8 x 10^{-3} min^{-1} (estimated in Table S6). Even so, one sees that the values of ε and s_i are not severely affected considering the following arguments. ε is independent of the ratio by definition (Eq. S1-39), whereas ρ_V in the autoinducer concentration s_i (Eq. 1) is multiplied by a factor of $(1+(\rho_V+\gamma_{ex}/c_{sec})\gamma_{in}/\gamma_{ex})^{-1}$ (Eq. S1-39). When we substitute the values for these parameters from biochemical studies ($\gamma_{in} = 10^{-2}$ min^{-1}; γ_{ex} and c_{sec} from Table S6; ρ_V from OD at the threshold in Table S6 and Eq. S3-4), we see that this factor ranges between 0.58 to 0.92. Accordingly, the threshold cell density increases by approximately inverse of the factor, i.e., 72% to 9%. Such deviation is within experiment-to-experiment variation (the standard deviation of OD at the threshold normalized by its mean is 25% and 9% for *las* and *rhl* operons respectively in Table S6). Thus, our conclusions hold even when intracellular degradation is taken into account (Fig. 3; Text S1 2).

1.6 Effect of diffusion

In the models (Text S1 1.1 ~ 1.4), the autoinducer molecule is assumed to diffuse fast so that spatial heterogeneity is negligible. Here, we estimate the spatial scale where this assumption is valid. Based on dimensional analysis, characteristic length L defined by diffusion coefficient D of the autoinducer is roughly given by

$$L \sim \sqrt{DT} \qquad \text{(S1-40)},$$

where T denotes the characteristic time scale which can be represented by the inverse of autoinducer degradation rate γ_{ex} (Eq. S1-1) in case of autoinduction and dual positive-feedback circuits (Fig. 1C-D). In case of the positive-and-negative feedback circuit (Fig. 1E) that exhibits oscillatory dynamics (Fig. 4), the time scale can be represented by the period of the oscillations. By substituting experimentally measured values of D and T into Eq. S1-40, we obtain L ~ 1 mm (Table S5). Therefore, the assumption of well-mixing holds for group-level dynamics that emerges at a few hundred micrometer scale or smaller.

2. Conditions for group-level / autonomous bistability

2.1 Group-level bistability in the autoinduction circuit

Group-level bistability means that when the synthetase concentration of all cells is uniform, i.e, $x_i = \bar{x} = \frac{1}{N_{cell}} \sum_j^{N_{cell}} x_j$ the population can take two stable steady states depending on the initial concentration. To derive sufficient condition for the group-level bistability, we analyzed steady state of Eq. 1. When $x_i = \bar{x}$ is substituted into Eq. 1, the steady state is given by

$$\left(1-\lambda^{-1}\right)\frac{\left(\left(\rho_V+\frac{\gamma_{ex}}{c_{\sec}}\right)\bar{x}\right)^2}{\left(\left(\rho_V+\frac{\gamma_{ex}}{c_{\sec}}\right)\bar{x}\right)^2+\bar{k}^2}+\lambda^{-1}-\bar{x}=0 \qquad \text{(S2-1),}$$

which can be rewritten as

$$f(\bar{x})=\bar{x}^3-\bar{x}^2+\left(\frac{\bar{k}}{\rho_V+\frac{\gamma_{ex}}{c_{\sec}}}\right)^2\bar{x}-\lambda^{-1}\left(\frac{\bar{k}}{\rho_V+\frac{\gamma_{ex}}{c_{\sec}}}\right)^2=0 \qquad \text{(S2-2).}$$

Bistability occurs when the equation has two stable and an unstable solutions. When the three states degenerate, bistability disappears via pitchfork bifurcation. At $\bar{x}=x_0$, Eq. S2-2 has a three-fold root [7] thus takes the form

$$f(\bar{x})=\left(\bar{x}-x_0\right)^3=0 \qquad \text{(S2-3).}$$

Eq. S2-2 satisfies

$$\begin{cases} f(x_0)=0, \\ \left.\frac{df}{d\bar{x}}\right|_{\bar{x}=x_0}=0, \\ \left.\frac{d^2f}{d\bar{x}^2}\right|_{\bar{x}=x_0}=0. \end{cases} \qquad \text{(S2-4).}$$

By substituting Eq. S2-2, we obtain

$$\begin{cases} \left(\dfrac{\bar{k}}{\rho_V + \dfrac{\gamma_{ex}}{c_{\sec}}} \right)^2 = \dfrac{x_0^2 \left(1 - x_0\right)}{x_0 - \lambda^{-1}}, \\ \left(\dfrac{\bar{k}}{\rho_V + \dfrac{\gamma_{ex}}{c_{\sec}}} \right)^2 = 2x_0 - 3x_0^2, \\ x_0 = \dfrac{1}{3}. \end{cases} \qquad \text{(S2-5)},$$

which yields

$$\begin{cases} x_0 = \dfrac{1}{3}, \\ \left(\dfrac{\bar{k}}{\rho_V + \dfrac{\gamma_{ex}}{c_{\sec}}} \right)^2 = \dfrac{1}{3}, \\ \lambda = 9. \end{cases} \qquad \text{(S2-6)}$$

When $\lambda > 9$, there are two stable states and one unstable steady state in intermediate cell density (red and yellow lines in Fig. 2B; closed and open circles in Fig. S1D), whereas the system is monostable irrespective of cell density when $\lambda < 9$.

The stability of each stable fixed point can be analyzed by plotting the activity of synthetase $d\bar{x}/dt$ as a function of $\bar{x}$ (Fig. S1F). When a sub-population switches to the ON-state, the activity (Eq. 1) of remaining population is approximatd by,

$$\frac{dx}{dt} \approx \left(1-\lambda^{-1}\right)\frac{\left(\rho_V\left((1-p)x+p\cdot 1\right)+\gamma_{ex}x/c_{\sec}\right)^2}{\left(\rho_V\left((1-p)x+p\cdot 1\right)+\gamma_{ex}x/c_{\sec}\right)^2+\bar{k}^2}+\lambda^{-1}-x \qquad \text{(S2-7)},$$

where p denotes fraction of cells in the ON-state (x_i ~ 1). Above a certain fraction of the on-state (e.g., $p = 0.02$, red line in Fig. S1F), the stable fixed point on the left (closed circle in Fig. S1F) corresponding to the OFF-state and the fixed point on the right (open circle in Fig. S1F) coalesce and disappears via saddle-node bifurcation so that the remaining population in the OFF-state inevitably switches to the ON-state.

2.2 Autonomous bistaiblity in the autoinduction circuit

The autonomous bistability requires that there are two stable states in an isolated condition ($\rho_V = 0$ for Eq. 1). To derive the phase boundary for the autonomous bistability, we analyze the steady state which is given by

$$\left(1-\lambda^{-1}\right)\frac{\left(\frac{\gamma_{ex}}{c_{\sec}}x_i\right)^2}{\left(\frac{\gamma_{ex}}{c_{\sec}}x_i\right)^2+k_i^2}+\lambda^{-1}-x_i=0 \qquad \text{(S2-8)}.$$

For simplicity, k_i can be replaced by the population mean $k_i = \bar{k}$. Thus we obtain

$$f\left(x_i\right)=x_i^3-x_i^2+\varepsilon^{-2}x_i-\lambda^{-1}\varepsilon^{-2}=0 \qquad \text{(S2-9)},$$

where $\varepsilon = \gamma_{ex}/c_{\sec}\bar{k}$ (Eq. S1-13). When one of the stable fixed points, and the unstable fixed point are degenerate, bistability disappears through saddle-node bifurcation

$$f\left(x_i\right)=\left(x_i-x_A\right)^2\left(x_i-x_B\right)=0 \qquad \text{(S2-10)},$$

where $x_i = x_A$ and x_B denote the fixed points. At $x_i = x_A$, Eq. S2-9 satisfies

$$\begin{cases} f(x_A) = 0, \\ \left.\dfrac{df}{dx_i}\right|_{x_i = x_A} = 0 \end{cases} \quad \text{(S2-11).}$$

By substituting Eq. S2-9, we obtain

$$\begin{cases} \varepsilon^{-2} = \dfrac{x_A^2(1-x_A)}{x_A - \lambda^{-1}} \\ \varepsilon^{-2} = 2x_A - 3x_A^2 \end{cases} \quad \text{(S2-12).}$$

By dividing the first equation by the second equation, a quadratic equation

$$2x_A^2 - \left(3\lambda^{-1} + 1\right)x_A + 2\lambda^{-1} = 0 \quad \text{(S2-13)}$$

is derived. We obtain x_A and ε as a function of λ

$$\begin{cases} x_A = \dfrac{1 + 3\lambda^{-1} \pm \sqrt{1 - 10\lambda^{-1} + 9\lambda^{-2}}}{4} \\ \varepsilon^{-2} = \dfrac{1 + 18\lambda^{-1} + 27\lambda^{-2} \pm \left(1 - 9\lambda^{-1}\right)\sqrt{1 - 10\lambda^{-1} + 9\lambda^{-2}}}{4} \end{cases} \quad \text{(S2-14).}$$

When $\lambda >> 1$, Eq. S2-14 is approximated to be

$$\begin{cases} x_A \approx \dfrac{1 + 3\lambda^{-1} \pm \left(1 - 5\lambda^{-1}\right)}{4} = \dfrac{1 - \lambda^{-1}}{2},\ 2\lambda^{-1} \\ \varepsilon \approx 2\left(1 - \lambda^{-1}\right),\ \dfrac{\sqrt{\lambda}}{2} \end{cases} \quad \text{(S2-15).}$$

The two solutions for ε define the phase boundaries between the group-level

bistability (GB) / autonomous bistaiblity (AB) and AB / monostability (M) shown in Fig. 3A, respectively.

The stability of each stable fixed point can be analyzed also for the population ($\rho_V > 0$) by plotting the production rate of synthetase dx_i / dt as a function of x_i (Fig. S1E). When all cells are in the OFF-state, i.e, $\bar{x} = V_{\max/\min}^{-1}$, the production rate is approximated by

$$\frac{dx}{dt} \approx \left(1-\lambda^{-1}\right)\frac{\left(\rho_V V_{\max/\min}^{-1} + \gamma_{ex} x / c_{\sec}\right)^2}{\left(\rho_V V_{\max/\min}^{-1} + \gamma_{ex} x / c_{\sec}\right)^2 + \bar{k}^2} + \lambda^{-1} - x \qquad \text{(S2-16).}$$

As cell density is increased, the basin of attraction for the OFF-state (region between left closed circle and open circle in Fig. S1E) decreases so that probability to switch from the OFF- to ON-state increases.

2.3 Phase boundary in the dual positive-feedback circuit

To derive the equations for the phase boundary in the dual positive-feedback circuit (Eq. 3), the derivation described above for the simple autoninduction circuit (Text S1 2.2) is applied to the dual positive-feedback circuit. The steady state in the isolated condition ($\rho_V = 0$ for Eq. 3 and Eq. S1-17) is given by

$$\left(1-\lambda^{-1}\right)\frac{x_i^n \left(\dfrac{\gamma_{ex}}{c_{\sec}} x_i\right)^{m\cdot n}}{x_i^n \left(\dfrac{\gamma_{ex}}{c_{\sec}} x_i\right)^{m\cdot n} + k_i^{m\cdot n}} + \lambda^{-1} - x_i = 0 \qquad \text{(S2-17),}$$

which rewrites to

$$f\left(x_i\right) = x_i^{m\cdot n+n+1} - x_i^{m\cdot n+n} + \varepsilon^{-m\cdot n} x_i - \lambda^{-1}\varepsilon^{-m\cdot n} = 0 \qquad \text{(S2-18),}$$

where $\varepsilon = \gamma_{ex} / c_{\sec}\bar{k}$ (Eq. S1-18). By applying Eq. S2-10 and S2-11 to Eq. S2-18,

we obtain

$$\begin{cases} \varepsilon^{-m\cdot n} = \dfrac{x_A^{m\cdot n+n}\left(1-x_A\right)}{x_A-\lambda^{-1}} \\ \varepsilon^{-m\cdot n} = \left(m\cdot n+n\right)x_A^{m\cdot n+n-1} - \left(m\cdot n+n+1\right)x_A^{m\cdot n+n} \end{cases} \quad \text{(S2-19).}$$

By dividing the upper by the lower equation, a quadratic equation

$$\left(m\cdot n+n\right)x^2 - \left(m\cdot n+n-1+\lambda^{-1}\left(m\cdot n+n+1\right)\right)x + \left(m\cdot n+n\right)\lambda^{-1} = 0 \quad \text{(S2-20)}$$

is derived. Thus we obtain

$$x_A = \frac{m\cdot n+n-1+\lambda^{-1}\left(m\cdot n+n+1\right) \pm \sqrt{\left(m\cdot n+n-1+\lambda^{-1}\left(m\cdot n+n+1\right)\right)^2 - 4\lambda^{-1}\left(m\cdot n+n\right)^2}}{2\left(m\cdot n+n\right)}$$

(S2-21).

When $\lambda >> 1$, these solutions can be approximated by

$$\begin{aligned} x_A &\approx \frac{m\cdot n+n-1+\lambda^{-1}\left(m\cdot n+n+1\right) \pm \left(m\cdot n+n-1\right)\left(1-\lambda^{-1}\dfrac{1+\left(m\cdot n+n\right)^2}{\left(m\cdot n+n-1\right)^2}\right)}{2\left(m\cdot n+n\right)} \\ &= \begin{cases} \dfrac{m\cdot n+n-1}{m\cdot n+n} - \lambda^{-1}\dfrac{1}{\left(m\cdot n+n\right)\left(m\cdot n+n-1\right)}, \\ \lambda^{-1}\dfrac{m\cdot n+n}{m\cdot n+n-1} \end{cases} \end{aligned} \quad \text{(S2-22).}$$

By substituting Eq. S2-22 into Eq. S2-19, the phase boundaries for the autonomous bistability are derived

$$\varepsilon \approx \begin{cases} \left(m\cdot n+n-1\right)^{-\frac{m\cdot n+n-1}{m\cdot n}}\left(m\cdot n+n\right)^{\frac{m\cdot n+n}{m\cdot n}}\left(1-\lambda^{-1}\dfrac{m+1}{m\cdot n+n-1}\right), \\ \lambda^{\frac{m\cdot n+n-1}{m\cdot n}}\left(m\cdot n+n-1\right)^{\frac{m\cdot n+n-1}{m\cdot n}}\left(m\cdot n+n\right)^{-\frac{m\cdot n+n}{m\cdot n}}. \end{cases} \quad \text{(S2-23).}$$

The upper and the lower solutions define the GB/AB boundary (solid line; Figs. 3B and S3) and AB/M boundary (dashed lines; Figs. 3B and S3), respectively. The value of parameter ε at the AB/M boundary increases steeply about 10 times when Hill coefficient is changed from $m = 1$ to $m = 4$ at $n = 1$ and $\lambda = 100$, whereas it increases twice to change from $n = 1$ to 4 at $m = 2$. ε at the GB/AB boundary decreases by half, according to change in $m = 1$ to 4 at $n = 1$, or $n = 1$ to 6 at $m = 2$. In summary, the parameter region of AB, i.e., ratio of AB/M and GB/AB boundaries expands according to increase in m and n (See also Figs. 3B and S3). The analysis shows that the auto-induciton circuit with dual positive-feedback loop is more robust to variation of parameter ε than the simple autoinduction (Eq. 1).

2.4 Cell-autonomous excitability in the positive-and-negative-feedback circuit

To derive the necessary condition for the cell-autonoumous excitability, we analyze the nullclines of isolated condition (Eq. S1-26). By "cell-autonomous" excitability, we mean that a transient response can be evoked by suprathreshold intrinsic noise in individual cells without relying on the secreted signal ($\rho\bar{s}$ in Eq. 4). In the state space $(x_i \geq 0, y_i \geq 0)$, the excitatory response appears as a large excursion from the stable fixed point (closed circle in Fig. S6A) towards a local maximum of the nullcline $dx_i/dt = 0$ (at $x_i \sim 0.5$ in Fig. S6A; Eq. S1-26) before returning back to the fixed point [22,25]. Thus the autonomous excitability requires the local maximum of y_i to be larger than 0 in the isolated condition ($\rho = 0$ for Eq. 4; $\varepsilon = 2$ and 4 in Fig. S6A). Otherwise there is no local maximum of the nullclines in the state space $(x_i \geq 0, y_i \geq 0)$ ($\varepsilon = 1$ in Fig. S6A) meaning no threshold response. When y_i is equal to 0 at the local maximum, the upper equation of Eq. S1-26 should satisfy

$$\begin{cases} y_i = 0, \\ \dfrac{dy_i}{dx_i} = 0 \end{cases} \quad \text{(S2-24).}$$

From the upper equation of Eq. S1-26, Eq. S2-24 is rewritten as

$$\begin{aligned} & x_i^3 - x_i^2 + \varepsilon^{-2} x_i - \lambda^{-1}\varepsilon^{-2} = 0 \\ & 2x_i^2 - \left(3\lambda^{-1} + 1\right) x_i + 2\lambda^{-1} = 0 \end{aligned} \quad \text{(S2-25),}$$

where k_i can be replaced by the population mean $k_i = \overline{k} = \varepsilon^{-1}$ (Eq. S1-24). Interestingly, the upper and lower equations of Eq. S2-25 are identical with Eqs. S2-9 and S2-13, respectively; the necessary conditions for autonomous bistability in the simple autoinduction circuit. Thus ε can similarly be defined by Eq. S2-15, i.e.,

$$\begin{cases} x_i \approx \dfrac{1-\lambda^{-1}}{2}, \;\; 2\lambda^{-1} \\ \varepsilon \approx 2\left(1-\lambda^{-1}\right), \;\; \dfrac{\sqrt{\lambda^{-1}}}{2} \end{cases} \quad \text{(S2-26).}$$

Since $x_i \approx \left(1-\lambda^{-1}\right)/2$ and $x_i \approx 2\lambda^{-1}$ correspond to the local maximum and minimum of the nullcline, respectively, we obtain

$$\varepsilon \approx 2\left(1-\lambda^{-1}\right) \quad \text{(S2-27).}$$

The solution defines the necessary condition for the autonomous excitability, i.e., $\varepsilon > 2\left(1-\lambda^{-1}\right)$ (on the right of solid black line in Figs. 5C and S6D-E).

3. Parameter estimation for bacterial quorum sensing

3.1 Normalized threshold $\bar{k}$

To calculate the order parameter ε (Eq. 2 and Fig. 3), we estimated the population mean of normalized threshold $\bar{k}$ (Eq. S1-11) in the actual bacteria. To this end, we adopted the following two methods.

3.1.1 Estimation of $\bar{k}$ from extracellular signal concentration

From Eq. S1-18 and S1-12, the normalized threshold in dual positive-feedback circuit (Eq. 3) is given by

$$\bar{k} = \frac{\gamma_X}{A_{syn} V_{\max} a_z^{\frac{1}{m}}} \gamma_{ex} \bar{K} = \frac{\gamma_{ex} \bar{K}}{A_{syn} X_{ON} a_z^{\frac{1}{m}}} \quad \text{(S3-1).}$$

Although A_{syn} can be estimated from the turnover rate of signal synthesis *in vitro*, concentration of the synthase X_{ON} and a_z, i.e., the ratio of Z and X at the steady state (Eqs. S1-16 and S1-17) were not available from literatures. Here we assumed $a_z = 1$ for simplicity. In case a_z is much lower than unity, i.e., concentration of LuxR (Z) is much lower than that of LuxI (X), the value of $\bar{k}$ must be $a_z^{-1/m}$ times larger than the estimated value in Text S1 3.2. For larger m, $a_z^{-1/m}$, i.e., the ratio between the real and the estimated value decreases to unity.

For X_{ON}, we tooled an alternative method based on the extracellular autoinducer concentration. From Eq. S1-5 and S1-7, we obtain

$$A_{syn} \bar{X} = \frac{\gamma_{ex}}{\rho_V} S_e \quad \text{(S3-2).}$$

After substituting Eq. S3-2 into $A_{syn}X_{ON}$, Eq. S3-1 becomes

$$\bar{k} = \bar{K} \frac{\rho_{V,ON}}{S_{e,ON}} \quad \text{(S3-3).}$$

$S_{e,ON}$ and $\rho_{V,ON}$ were estimated from the extracellular autoinducer concentration and the cell density at the time gene expression within the referencing operon, e.g., *luxI* or *luxR*, is fully induced. The volume cell density ρ_V is converted to the optical density (OD) reading using the proportional constant

$$\frac{\rho_V}{OD} = 3.6 \cdot 10^{-3} \qquad \text{(S3-4)},$$

which depends little on growth medium and growth rate of population in two strains of *E. coli* (Table 1 and Fig. 2A in ref. [26]; see also [27]). We estimated $\bar{K}$ from threshold concentration of autoinducer where expression of genes within the operon takes the half maximum.

3.1.2 Estimation of $\bar{k}$ from gene expression level

To estimate a set of parameters for both *luxI* and *luxR* feedback regulation, Thattai and colleagues examined engineered *E. coli* strains, each with different parts of the dual positive-feedback gene circuit, so as to separately analyze the feedback components [7]. Following their study (Eq. S29 in ref.[7]), the normalized threshold of the dual positive-feedback circuit (Eq. 3) equates to

$$\bar{k} = \frac{\rho_V}{OD} \frac{1}{\left(Q_R a_z\right)^{\frac{1}{m}} \tilde{\mu} Q_I} = \frac{2.6 \cdot 10^{-5}}{\sqrt{a_z}} \qquad \text{(S3-5)},$$

where m, Q_I, Q_R denote Hill coefficient of AHL-LuxR binding, protein production rates per transcript of *luxI* and *luxR* scaled by the protein decay rates (Eq. 4 in ref.[7]; Table S6), respectively. $\tilde{\mu}$ is ratio of autoinducer concentration and *luxI* expression level (Eqs. 1 and S14 in ref.[7]). Value of the parameters were substituted from Table S3 in ref. [7]. a_z denotes the concentration ratio of LuxR divided by LuxI (Eqs. S1-18 and S1-17), which was assumed to take between 1 and 0.01 [7] due to different translation rates. Here we assumed a_z = 1.

3.2 The order parameter ε

Here we estimate the value of the order parameter $\varepsilon = \gamma_{ex}/c_{\sec}\bar{k}$ (Eq. 2; Fig. 3B) in four well-studied autoinducer operons *rhl*, *las*, *car* and *lux*. To estimate $\bar{k}$, we applied Eq. S3-5 to one data set in *lux* system, and Eq. S3-3 to the other data sets, respectively. In Eq. S3-3, $\bar{k}$ can be estimated from the autoinducer concentration and cell density (Eq. S3-2) at the time when expression of genes within the quorum-sensing operon are fully induced. For the rates of secretion c_{sec} and degradation γ_{ex}, we adopted export [9,28] and hydrolysis [28] rate constants of AHLs, respectively, following the earlier study [29]. We assumed the oxo- and hydroxyl- derivatives have an identical hydrolysis rate. Table S6 summarizes the set of parameters that are estimated in detail below. Moreover, the estimation applies to the positive-and-negative-feedback circuit (Fig. 1E), since the expression of ε (Eq. S1-24) is identical with that of the autoinduction circuit $\varepsilon = \gamma_{ex}/c_{\sec}\bar{k}$ (Eq. 2).

3.2.1 *rhl* system

In *Pseudomonas aeruginosa*, the autoinducer N-butyryl-L-Homoserine lactone (C4-HSL) is produced from S-adenosylmethionine by the synthetase RhlI. Michaelis-constant of the enzymatic reaction is much lower than the intracellular concentration of the substrate [30] indicating that substrates are not rate-limiting. Thus we can assume the synthesis rate depends mainly on the synthetase concentration. A transcriptional activator RhlR forms a complex with C4-HSL that binds to a promoter at the upstream of *rhl* operon. At high cell density, C4-HSL induces expression of genes within the operon: e.g., the synthetase gene *rhlI*, the transcriptional activator gene *rhlR*, and *rhlA* that encodes surfactant rhamnolipid which is a virulence factor [31] used for biofilm formation [32]. Therefore, concentrations of RhlI and RhlR play the role of X and Z in the dual positive-feedback circuit (Eq. S1-15).

To calculate $\bar{k}$ and ε in Eq. S1-18 based on Eq. S3-3, we estimated the

extracellular C4-HSL concentration $S_{e,ON}$, the cell density $\rho_{V,ON}$ (Eqs. S3-3 and S3-4), the threshold of C4-HSL concentration $\bar{K}$, the rates of secretion c_{sec} and degradation γ_{ex} of C4-HSL. The extracellular concentration of C4-HSL in a batch culture is $S_{e,ON}$ = 26 μM at cell density of OD = 2.0 where *rhlR* expression is induced (Figs. 4B and 3B in ref.[33]). Based on measurement of C4-HSL concentration at half-maximal induction of both *rhlA-lacZ* reporter activity and rhamnolipid synthesis (0.5 μM from Fig. 4B in ref.[34] and 0.5 μM from Fig. 5B in ref.[35], respectively), the estimated threshold for the response by the cells to 3-oxo-C6-HSL was $\bar{K}$ = 0.5 μM. We substituted c_{sec} = 2 min^{-1} into the secretion rate, based on measurement of radioactive C4-HSL exported from *P. aeruginosa* cells [9]. The degradation rate of C4-HSL estimated from hydrolysis of 3-oxo-C4-HSL *in vitro* is γ_{ex} = 4.8 x10^{-4} min^{-1} [28]. When these data are substituted into Eqs. S3-3, 2 and S3-4, we obtain

$$\begin{cases} \bar{k} = \bar{K}\dfrac{\rho_{V,ON}}{S_{e,ON}} = 0.5\dfrac{2.0}{26}0.0036 = 1.4\cdot 10^{-4}, \\ \varepsilon = \dfrac{\gamma_{ex}}{c_{\sec}\bar{k}} = \dfrac{0.00048}{2\bar{k}} = 1.7. \end{cases}$$

To verify the above estimation, we compared it with an independent experimental report showing that *rhlR* expression becomes fully induced when the extracellular C4-HSL concentration reaches 5.8 μM at OD = 2.4 (Figs. 5B and 4B in ref.[36]). Thus we obtain

$$\begin{cases} \bar{k} = \bar{K}\dfrac{\rho_{V,ON}}{S_{e,ON}} = 0.5\dfrac{2.4}{5.8}0.0036 = 7.4\cdot 10^{-4}, \\ \varepsilon = \dfrac{\gamma_{ex}}{c_{\sec}\bar{k}} = \dfrac{0.00048}{2\bar{k}} = 0.32. \end{cases}$$

The difference between the two estimation is within one order of magnitude. The results are plotted in Fig. 3B.

3.2.2 *las* system

In *P. aeruginosa*, synthesis of another AHL,

N-3-oxo-dodecanoyl-L-Homoserine lactone (3-oxo-C12-HSL), is catalyzed by the synthetase LasI. When a complex of AHL and transcriptional activator LasR binds to the promoter of the *las* operon, it induces expression of genes in the operon such as the synthetase gene *lasI,* the transcriptional activator gene *lasR* and a virulence gene *lasB*. Here we estimated a set of the parameters of 3-oxo-C12-HSL in Eqs. S3-3 and 2, following the same scheme described above for the *rhl* system. The extracellular concentration of 3-oxo-C12-HSL in a batch culture is 4.6 μM at cell density of OD = 2.5 at a time when *lasI* expression is fully induced (Figs. 2F and 2C in ref.[37]). According to a study based on *lasB-lacZ* reporter activity, the threshold of autoregulatory response by the cells to 3-oxo-C12-HSL was $\bar{K}$ = 1 μM (Fig. 1A in ref.[38]). The secretion rate of c_{sec} = 0.2 min^{-1} was estimated from measurement of tritium labeled 3-oxo-C12-HSL exported from *P. aeruginosa* [9]. We estimated the degradation rate as γ_{ex} = 2.9 x10^{-4} min^{-1} based on the rate of hydrolysis of 3-oxo-C12-HSL *in vitro* [28]. By substituting these parameters into Eqs. S3-3, 2 and, S3-4, we obtain

$$\bar{k} = 2.0 \cdot 10^{-3}, \ \ \varepsilon = 0.74.$$

As in the *rhl* systems, we compared the above estimation with another independent experimental result showing that *lasR* expression becomes fully induced when extracellular 3-oxo-C12-HSL concentration reaches 5.5 μM at OD = 1.5 (Figs. 4A and 2 in ref.[33]). By substituting these parameters into Eqs. S3-3, 2 and S3-4, we obtain

$$\bar{k} = 9.8 \cdot 10^{-4}, \ \ \varepsilon = 1.5$$

The difference of $\bar{k}$ and ε between the two cases is below one order of the magnitude.

3.2.3 *car* system

In plant pathogen *Erwinia carotovora*, production of an AHL N-3-oxo-hexanoyl-L-Homoserine lactone (3-oxo-C6-HSL), is catalyzed by the synthetase CarI to induce state of virulence at high cell density. The

autoinduction works through its binding to the transcriptional activator CarR. Here we estimate a set of parameters on 3-oxo-C6-HSL in *car* operon to calculate $\bar{k}$ in Eq. S3-3 and ε in Eq. 2. The extracellular concentration of 3-oxo-C6-HSL in batch cultures is about 8 μM at cell density of OD = 3.4 which is just before the stationary phase (Fig. 5a in ref.[39]). Based on the measurements of 3-oxo-C6-HSL binding to CarR (1.5 μM, Table 3 in ref.[40]; 2.0 μM, Fig. 3 in ref.[41]), the estimated threshold for the autoregulatory response by the cells to 3-oxo-C6-HSL was $\bar{K}$ = 1.75 μM. 3-oxo-C6-HSL freely diffuses out from *V. fischeri* cells at a rate c_{sec} = 3 min^{-1} [42]. We assumed that *E. carotovora* also secrete at the identical rate. The degradation rate is estimated at γ_{ex} = 1.8 x10^{-3} min^{-1} based on the rate of hydrolysis of 3-oxo-C6-HSL *in vitro* [28]. Thus we obtain

$$\bar{k} = 2.7 \cdot 10^{-3}, \ \ \varepsilon = 0.22.$$

3.2.4 *lux* system

In *V. fischeri*, synthetase LuxI catalyzes production of 3-oxo-C6-HSL. When a complex of 3-oxo-C6-HSL and transcriptional activator LuxR binds to the promoter of the *lux* operon, it induces expression of the synthetase gene *luxI,* the transcriptional activator gene *luxR* and genes *luxAB* that encode subunits of luciferase required for their luminescence at high cell density. The autoinduction works by its binding to the transcriptional activator LuxR. We estimated a set of parameters for the *lux* operon to obtain the values of $\bar{k}$ in Eq. S3-3 and ε in Eq. 2. Extracellular concentration of 3-oxo-C6-HSL in a batch culture is 18 μM when the bioluminescence is fully induced at cell density of OD = 1.0 (Fig. 5c in ref.[39]). Based on the measurements of 3-oxo-C6-HSL concentration at half-maximum of LuxR binding to DNA (0.07 μM, Fig. 2A in ref.[15]) and bioluminescence (< 0.12 μM, Table 1 in ref.[43]; < 0.2 μM, Fig. 2 in ref.[42]; 0.12 ~ 0.2 μM, Fig. 6B in ref.[6]; 0.12 ~ 0.18 μM, Table 1 in ref.[44]), the estimated threshold concentrations of 3-oxo-C6-HSL was $\bar{K}$ = 0.1 μM. We substituted c_{sec} = 3 min^{-1}, based on the measurement of radioactive

3-oxo-C6-HSL exported from *V. fischeri* [42]. The degradation rate of 3-oxo-C6-HSL γ_{ex} = 1.8 x10^{-3} min^{-1} was inferred from that of the *car* system. Thus we obtain

$$\bar{k} = 2.0 \cdot 10^{-5}, \ \varepsilon = 30.$$

When we compared this to another estimate based on independent experiment of *lux* system [7] using Eq. S3-5 instead of Eq. S3-3 and substituting a_z = 1 and the same estimated values of c_{sec} and γ_{ex} described above, we obtain

$$\varepsilon = \frac{\gamma_{ex}}{c_{\sec}} \frac{1}{\bar{k}} = \frac{0.0018}{3.0} \frac{\sqrt{a_z}}{2.6 \cdot 10^{-5}} = 23\sqrt{a_z} = 23.$$

The difference of $\bar{k}$ between the two cases is below one order of magnitude. ε in the two cases are about 10 times larger than AB/GB boundary $\varepsilon \sim 2$ (Eqs. S2-15 and S2-23; solid lines in Fig. 3B). These value might be an overestimate if $a_z \ll 1$. Because the expression of ε is identical for the simple autoinduction circuit and the positive-and-negative feedback circuit (Eq. 2; Eq. S1-24), the above estimate is valid for the engineered *E. coli* that harbors a negative feedback of *luxI* gene downstream of *lux* promoter in *E. coli* [18]. As long as the value of $\bar{k}$ is close to the above estimate, it is reasonable to expect $\varepsilon > 2$ suggesting a graded transition for the oscillatory circuit.

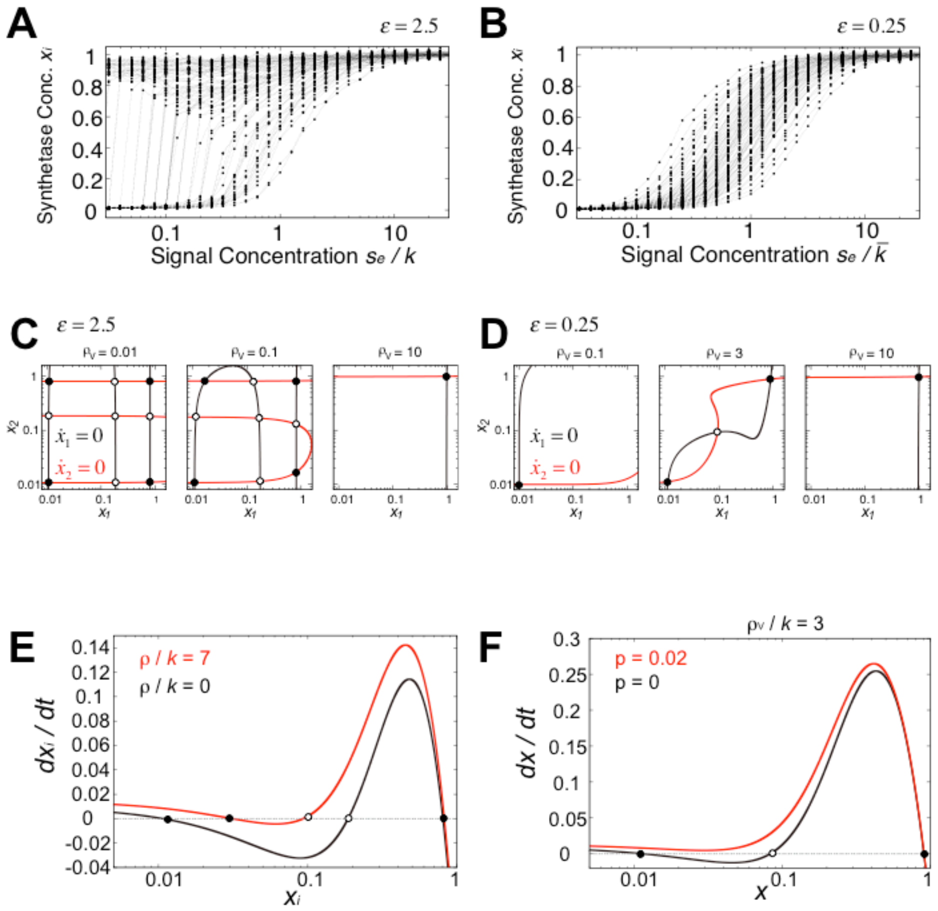

Figure S1. Supporting figure for Figure 2.

(A)-(B) Heterogeneous response of isolated cells (N = 100) to exogenously applied autoinducer due to cell-cell heterogeneity in k_i,. $\rho_V \bar{x}$ in Eq. 1 is replaced by exogenous signal concentration s_e. **(C)-(D)** Nullclines of Eq. 1 in case of two cells, where the values of $\varepsilon = \gamma_{ex} / c_{\mathrm{sec}} \bar{k}$ and λ are identical with those used in Figs. 2A and 2B, respectively. Closed and open circles indicate stable and unstable fixed points, respectively. At the both stable fixed points, x_1 and x_2 are identical indicating group-level bistability. $(x_1, x_2) \sim (1, 0.01)$ and $(0.01, 1)$ are also allowed in **(B)** indicating coexistence of ON and OFF states. **(E)-(F)** Activity of synthetase dx / dt plotted as a function of x; autonomous (**E**; Eq. S2-16) and group-level (**F**; Eq. S2-7) bistability. The values of ε in **(E)** and **(F)** are identical with Figs. 2A and 2B, respectively.

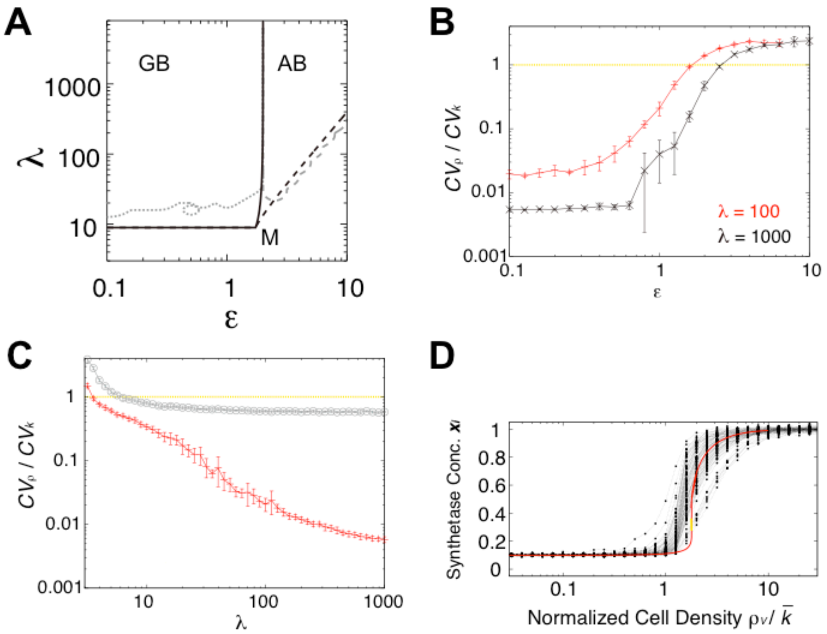

Figure S2. Supporting figure for Figure 3A.

(A) Phase boundaries AB/GB (solid line), AB/M (dashed line), and GB/M (dotted line) determined analytically (black, Eqs. S2-6 and S2-14) and numerically (grey). In numerical simulations of cell population (Eq. 1), the AB phase is assigned when the ON- and OFF-state cells coexisted at the steady state. The GB phase is assigned when the entire population uniformly takes either the ON- or the OFF state at all density. **(B)-(C)** CV_ρ/CV_k plotted as a function of ε for λ = 100 and 1000 **(B)** and λ for ε = 0.25 **(C)**, respectively, for the simple autoinduction circuit (Eq. 1). In case of group-level bistabiltiy ($\varepsilon < 2$ in **(B)**; red points in **(C)**), CV_ρ/CV_k decreases with decreasing ε and increasing λ. On the other hand, CV_ρ is approximately equal to the intrinsic variation CV_k, when the group-level bistability disappears ($\varepsilon > 2$ in **(B)** for autonomous bistability; grey points in **(C)** represent simple autoinduction without cooperativity $\frac{dx_i}{dt}=\left(1-\lambda^{-1}\right)\frac{\rho_V\bar{x}+\gamma_{ex}x_i/c_{\sec}}{\rho_V\bar{x}+\gamma_{ex}x_i/c_{\sec}+k_i}+\lambda^{-1}-x_{\boldsymbol{i}}$). **(D)** The response of synthetase concentration x_i to cell density ρ is highly variable between the cells at λ = 10 compared to λ = 100 (Fig. 2B). The value of ε and standard deviation of k_i are the same as Fig. 2B. Each point in indicates time average at the steady state.

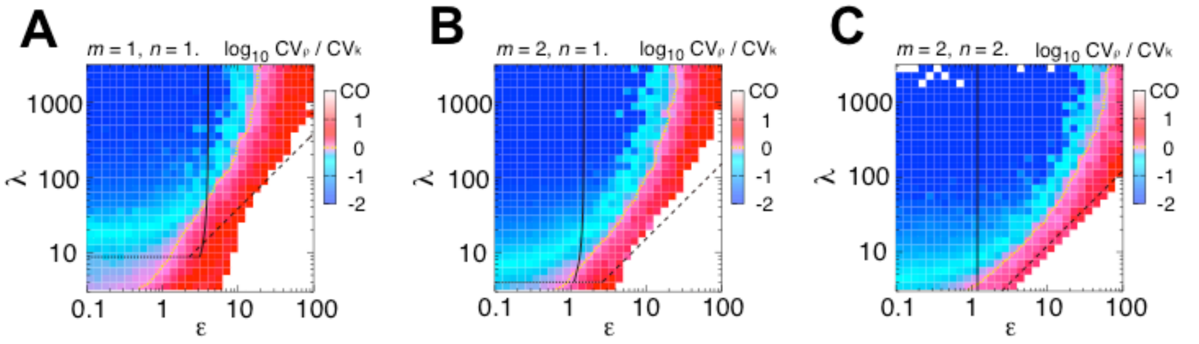

Figure S3. Supporting figure for Figure 3B.

(A)-(C) Phase diagram of CV_ρ/CV_k in dual positive-feedback circuit (Eq. 3). (m, n) = (1, 1) **(A)**, (2, 1) **(B)**, and (2, 2) **(C)**, respectively. Solid, dashed and dotted black lines indicate analytically determined boundary AB/GB (Eq. S2-23), AB/M (Eq. S2-23), and GB/M, respectively. Yellow line is $\log_{10} CV_\rho/CV_k = 0$ determined numerically.

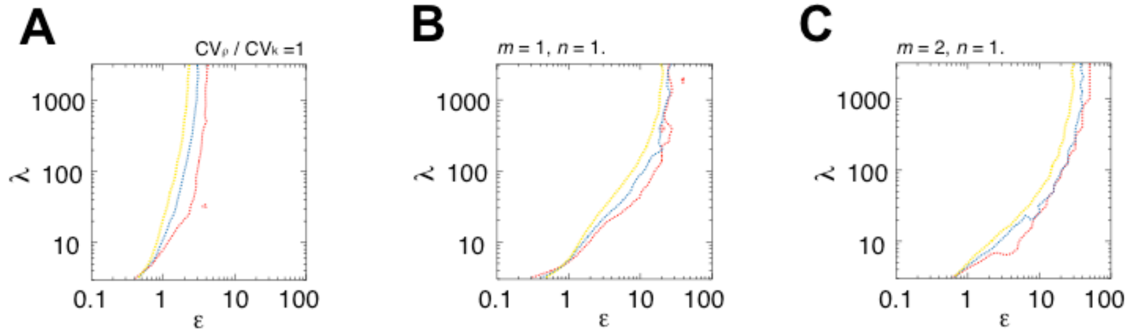

Figure S4. Growth rate dependence of the phase diagram.

The boundary line between all-or-none and graded transition, i.e., $CV_\rho/CV_k = 1$ is plotted for three different increasing rate of cell density, i.e., growth rate (Models). The ratio of the increasing rate to the degradation rate of synthetase (γ_X in Eq. S1-3) is set to 1/2 (red), 1/10 (blue) and 1/40 (yellow), respectively, for the simple autoinduction circuit (**A**; Fig. 1C) and the dual positive-feedback circuit (**B**-**C**; Fig. 1D). The yellow line was imported from Figs. 3A. S3A, and S3B to **(A)**, **(B)** and **(C)**, respectively. The boundary lines are almost independent of the growth rate.

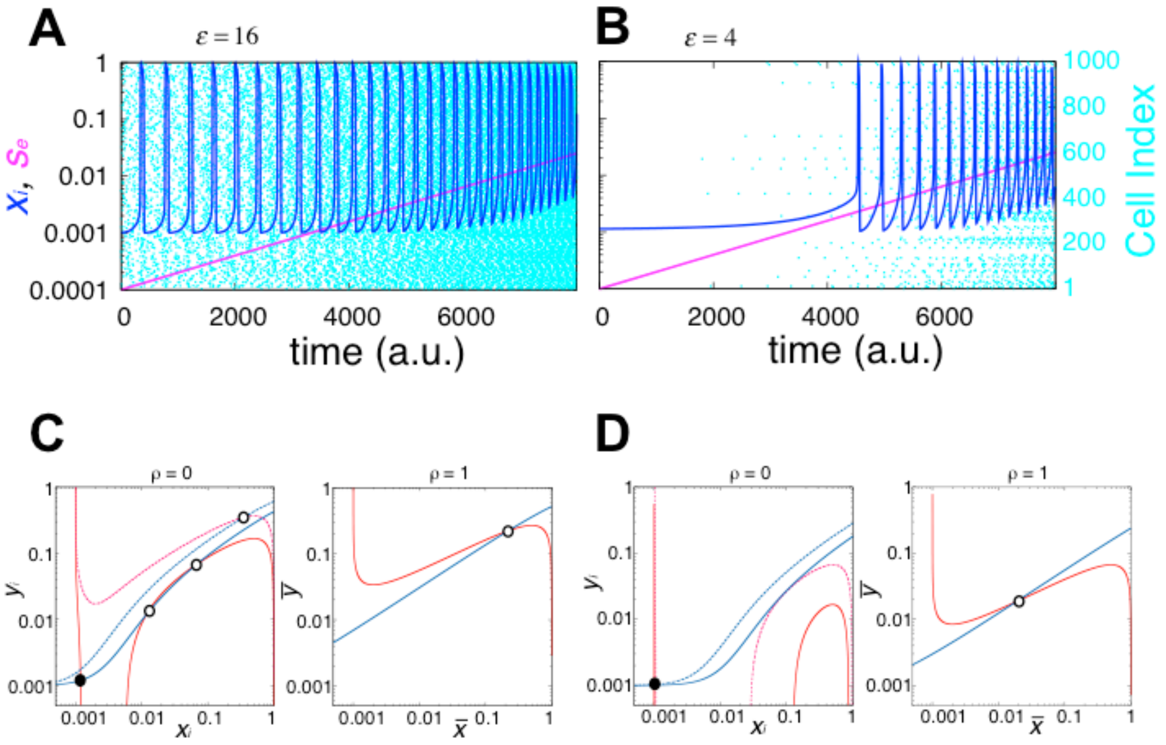

Figure S5. Supporting figure for Figure 4.

(A)-(B) Heterogeneous response of synthetase concentration x_i in isolated cells (blue line: time course of a representative cell; light blue point: pulsatile response of each cell indexed in Y-axis). $\rho\bar{x}$ in Eq. 4 is replaced by the extracellular autoinducer concentration s_e that is applied exogenously as an exponentially increasing function (violet line). ε = 16 in (A) and 4 (B) as in Figs. 4A and 4B, respectively. $\lambda = 10^3$ and $g = 30$ in (A) and (B). At $s_e = 0$, a fraction of cells are already oscillatory in (A), whereas all cells are quiescent in (B). (C)-(D) Nulcllines of isolated condition (Eq. S1-26) for ρ = 0 and population mean (Eq. S1-28) for $\rho = 1$. Value of the parameters in (C) and (D) are same with (A) and (B) respectively. At ρ = 0, the i-th cell is either excitatory or oscillatory depending on k_i (C). In (D), cells are always excitatory regardless of k_i. At $\rho = 1$, all cells are oscillatory in both (C) and (D). Red and blue lines indicate dx/dt = 0 and dy/dt = 0, respectively. Solid and dotted lines at ρ = 0 indicate k_i is 50% larger and smaller than $\bar{k} = \varepsilon^{-1}$ (Eq. S1-24).

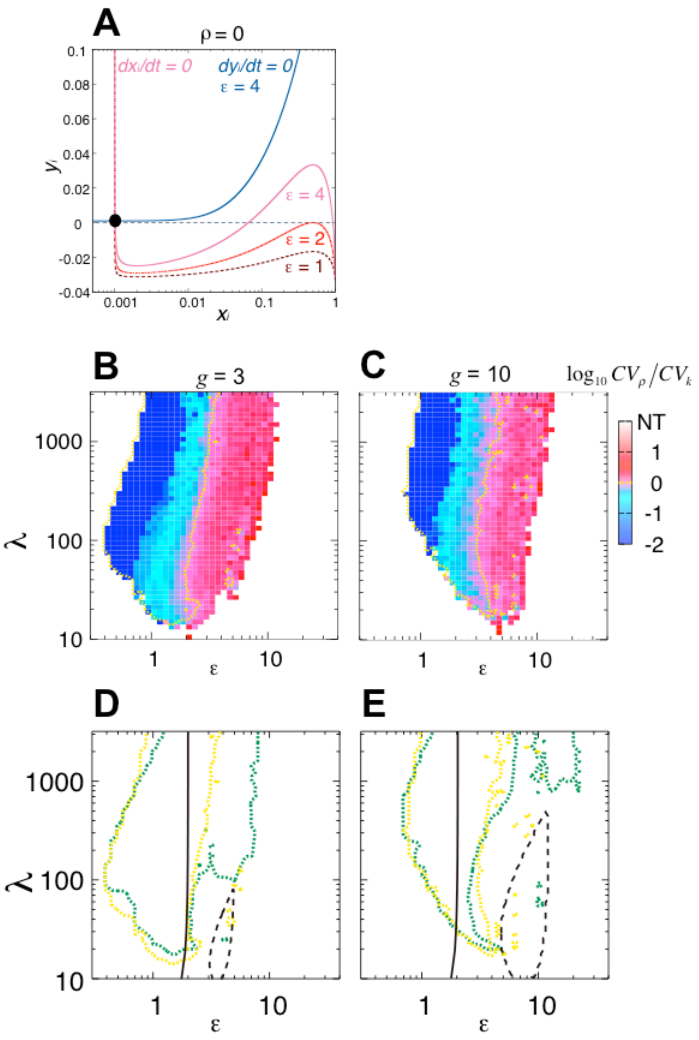

Figure S6.

Supporting figure for Figure 5.

(A) The necessary condition for cell-autonomous excitability. $dx_i/dt = 0$ in isolated condition (Eq. S1-26) for $\varepsilon = 1$, 2 and 4 ($1/k_i = \varepsilon$ in Eq. S1-24). For ease of view, $dy_i/dt = 0$ is plotted only for $\varepsilon = 4$ (light blue line). The local maximum ($x_i \sim 0.5$) is positive at $\varepsilon = 4$, zero at $\varepsilon = 2$ and negative at $\varepsilon = 1$ indicating no excitability at $\varepsilon = 1$ and 2. Thus cell-autonomous excitability requires $\varepsilon > 2$, consistent with analytical derivation (Text S1 2.4). **(B)-(C)** Phase diagram of CV_ρ/CV_k for $g = 3$ **(B)** and 10 **(C)** in presence of intrinsic noise (η_i in Eq. 4; $|\eta_i| = 0.1$). The other parameters are identical with Fig. 5. **(D)-(E)** $\log_{10}$ $CV_\rho/CV_k = 0$ plotted for cases with noise (yellow dotted line) or without (green dotted line). Black solid line indicates the excitable/oscillatory boundary $\varepsilon \sim 2$ derived analytically (Eq. S2-27). The region surrounded by black dashed curves supports autonomous oscillations in isolated condition in numerical simulations. $g = 3$ **(D)** and 10 **(E)**.

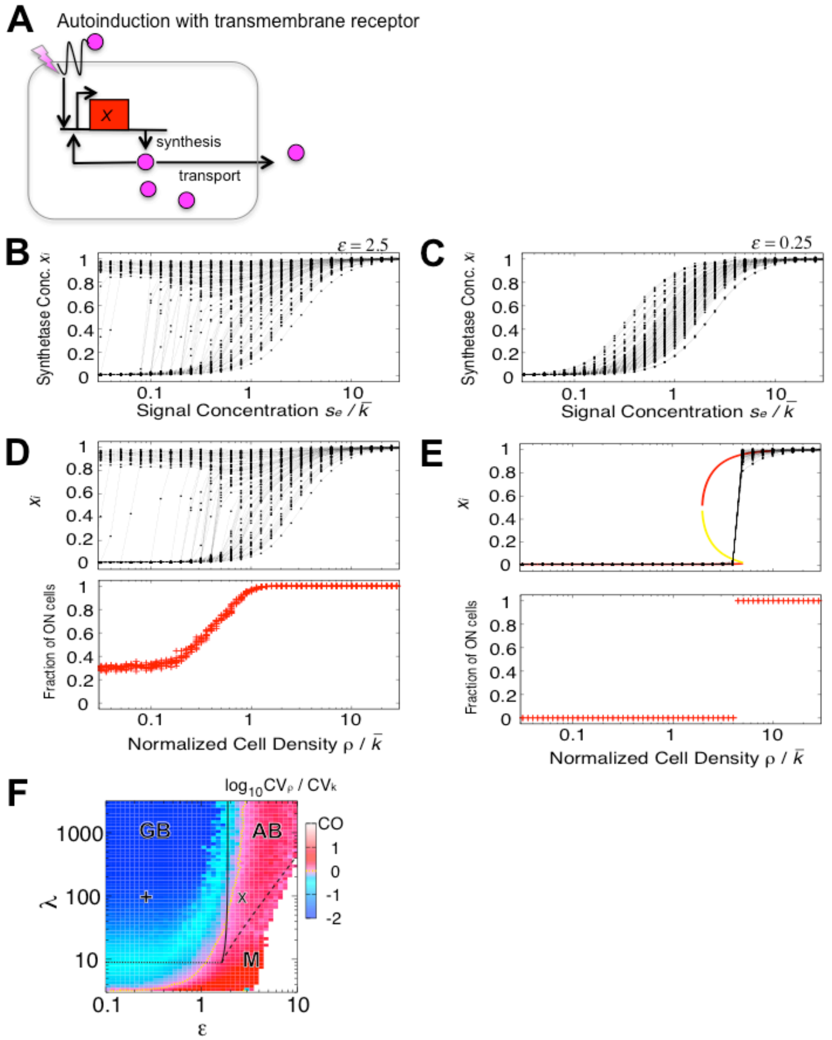

Figure S7. Graded and all-or-none quorum sensing transitions in a trans-membrane receptor model. (A) Schematics of an autoinduction circuit with transmembrane receptor model (Eq. S1-32) in operation. (B)-(C) Heterogeneous response in isolated cells to exogeneously applied autoinducer signal (Eq. S1-32; k_i has intrinsic variability as in Eq. 1; $|\eta_i| = 0.1$). $\varepsilon = 2.5$ (B) and 0.25 (C). $\lambda = 100$. Bistability appears cell-autonomously in (B) but not in (C). (D) Autonomous bistability and (E) group-level bistability (red line, analytical solution for the population mean) underlie graded and all-or-none transitions, respectively. Parameters in (D) and (E) are identical with (B) and (C), respectively. (F) Phase diagram of CV_ρ/CV_k. Solid, dashed and dotted black lines indicate analytically determined boundary AB/GB, AB/M, and GB/M, respectively. Yellow line is $\log_{10} CV_\rho/CV_k = 0$ determined numerically. x and + correspond to (D) and (E), respectively.

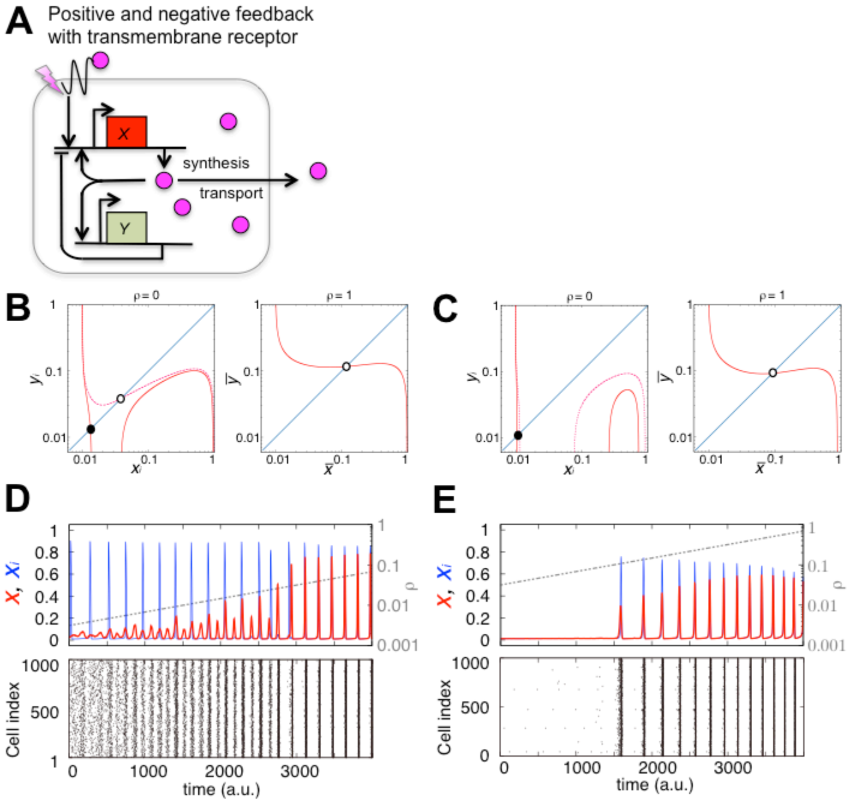

Figure S8. Dynamical quorum sensing transitions in a negative-feedback mediated trans-membrane receptor model.

(A) Schematics of positive-and-negative feedback circuit with transmembrane receptor model (Eq. S1-34). **(B)-(C)** Nullclines for the isolated cell (Eq. S1-36; left panel) and the population (Eq. S1-37; right panel) predict transitions from quiescence to oscillations depending on cell density ρ. Similar to the direct import model (Eq. 4; Fig. S5C-D), at $\rho = 0$, cells are either excitatory or oscillatory depending on k_i in case of $\varepsilon = \alpha/\bar{k} = 5.6$ **(B)**. Cells are always excitatory irrespective of k_i in $\varepsilon = \alpha/\bar{k} = 2.8$ **(C)**. Solid and dotted lines at $\rho = 0$ indicate k_i is 25% larger and smaller than $\bar{k} = \varepsilon^{-1}$ (Eq. S1-24). $\alpha = 2.4$, $\beta = 10$, and $\lambda = 100$ in **(B)** and **(C)**. **(D)-(E)** Simulations of communicating cell populations demonstrate graded **(D)** and all-or-none **(E)** transitions during exponential increase in cell density ρ (grey line). The randomized parameter k_i has a lognormal distribution with $CV_k = 0.25$. $\varepsilon = \alpha/\bar{k}$ in **(D)** and **(E)** are the same with **(B)** and **(C)**, respectively. λ, α, and β in **(D)** and **(E)** are also identical with **(B)** and **(C)**. $a_y = 100$. $|\eta_i| = 0.0$.

Supporting Tables

Table S1. Representative examples of autoinduction.

Species	Signal	Autoinduction λ	Hill coefficient	Ref.
E. coli	AHL	36	2	[7]
P. putida	AHL	10	2.5	[3]
D. discoideum	cAMP	10~40	N/A	[20,45]

Table S2. List of variables and parameters of autoinduction kinetics Eq. S1-1 to Eq. S1-3.

i	Index of cells
S_e	Extracellular signal concentration
S_i	Intracellular signal concentration
$\bar{S}$	Population mean of S_i
X_i	Concentration of signal synthetase
ρ_V	Volume cell density, i.e., volume fraction of cells $\rho_V = N_{cell}V_{cell} / V_{tot}$, where N_{cell}, V_{cell}, and V_{tot} denote number of cells, volume of single cell and culture volume, respectively.
c_{sec}	Secretion rate of signal molecule
A_{syn}	Synthesis rate of signal molecule
γ_X	Degradation rate of the synthetase
γ_{ex}	Extracellular degradation rate of signal molecule
K_i	Threshold concentration of signal response
$\bar{K}$	Population mean of K_i

Table S3. Parameter values chosen in dual positive-feedback circuit Eq. 3.

Parameters in present model	Expression in earlier model [7]	Present model (Figs. 3B and S3)	Earlier model
$\bar{k}$	$\frac{\rho_V}{OD}\frac{1}{(Q_R A)^{1/m}\tilde{\mu}Q_I}$	6 x 10^{-6} ~ 6 x 10^{-3} #	7 x 10^{-6} ~ 7 x 10^{-5} *
a_Z	A	1	0.01 ~ 1
m	m	1 ~ 2	2
n	n	1 ~ 2	1.45

# Range of ε in Fig. 3B is calculated from Eq. 2 by substituting rates of degradation γ_{ex} = 1.8 x10^{-3} min^{-1} and transport c_{sec} = 3 min^{-1} which were estimated from literatures (Text S1 3.2.4).

* The parameters were referred to Table S3 in ref.[7], except for OD / ρ_V from Eq. S3-4.

Table S4. Parameter values chosen in positive-and-negative feedback circuit Eq. 4.

Parameters in present model	Expression in earlier model [18]	Present model (Figs. 4, 5, S5 and S6)	Earlier model *
λ	$\frac{\alpha}{\delta k_1}$	10^1 ~ 3 x 10^3	2.5 x 10^7
G	$\frac{C_I\alpha}{k_1}\frac{\gamma_H}{\gamma_A D}$	3 ~ 30	26.7
$\bar{k}$	$\frac{\gamma_I D k_1}{C_A \alpha b}$	0.03 ~ 3	0.01
a_Y	γ_I / γ_A	100	1.6
a_S	γ_I / D	1	9.6

* Calculated from Supplementary Information of ref. [18].

Table S5. Dimensional analysis of spatial scale of extracellular environment.

	Autoinducer	Diffusion coefficient D	Time scale T	Spatial scale L
V. fischeri	AHL	550 μm^2/sec [#1]	560 min [*1]	4.3 mm
engineered *E. coli*	AHL	550 μm^2/sec	50~80 min [*2]	1.3~1.6 mm
D. discoideum	cAMP	250~1,000 μm^2/sec [#2]	300~600 sec [*3]	0.27~0.77 mm

[#1]: [46,47]. [#2]: [48-50]. [*1]: Inverse of hydrolysis rate of AHL *in vitro* [28]. [*2]: Oscillation period [18]. [*3]: Oscillation period [20].

Table S6. Parameters estimated from literatures.

System	$S_{e,ON}$ μM	OD	$\bar{K}$ μM	γ_{ex} min^{-1}	c_{sec} min^{-1}	$\bar{k}$	ε
rhl	26	2.0	0.5	4.8×10^{-4}	2.0	1.4×10^{-4}	1.7
	5.8	2.4				7.4×10^{-4}	0.32
las	4.6	2.5	1.0	2.9×10^{-4}	0.2	1.9×10^{-3}	0.74
	5.5	1.5				9.8×10^{-4}	1.5
car	8	3.4	1.75	1.8×10^{-3}	3.0	3.7×10^{-3}	0.22
lux	40	2.0	0.1	1.8×10^{-3}	3.0	2.0×10^{-5}	32
	N/A					2.6×10^{-5}	23

References

1. James S, Nilsson P, James G, Kjelleberg S, Fagerstrom T (2000) Luminescence control in the marine bacterium Vibrio fischeri: An analysis of the dynamics of lux regulation. J Mol Biol 296: 1127-1137.
2. Dockery JD, Keener JP (2001) A mathematical model for quorum sensing in Pseudomonas aeruginosa. Bull Math Biol 63: 95-116.
3. Fekete A, Kuttler C, Rothballer M, Hense BA, Fischer D, et al. (2010) Dynamic regulation of N-acyl-homoserine lactone production and degradation in Pseudomonas putida IsoF. FEMS Microbiol Ecol 72: 22-34.
4. Maamar H, Dubnau D (2005) Bistability in the Bacillus subtilis K-state (competence) system requires a positive feedback loop. Mol Microbiol 56: 615-624.
5. Suel GM, Kulkarni RP, Dworkin J, Garcia-Ojalvo J, Elowitz MB (2007) Tunability and noise dependence in differentiation dynamics. Science 315: 1716-1719.
6. Perez PD, Hagen SJ (2010) Heterogeneous response to a quorum-sensing signal in the luminescence of individual Vibrio fischeri. PLoS One 5: e15473.
7. Rai N, Anand R, Ramkumar K, Sreenivasan V, Dabholkar S, et al. (2012) Prediction by promoter logic in bacterial quorum sensing. PLoS Comput Biol 8: e1002361.
8. Tu KC, Long T, Svenningsen SL, Wingreen NS, Bassler BL (2010) Negative feedback loops involving small regulatory RNAs precisely control the Vibrio harveyi quorum-sensing response. Mol Cell 37: 567-579.
9. Pearson JP, Van Delden C, Iglewski BH (1999) Active efflux and diffusion are involved in transport of Pseudomonas aeruginosa cell-to-cell signals. J Bacteriol 181: 1203-1210.
10. Gillespie DT (1977) Exact Stochastic Simulation of Coupled Chemical Reactions J Phys Chem 21: 2340-2361.

11. Shibata T, Fujimoto K (2005) Noisy signal amplification in ultrasensitive signal transduction. Proc Natl Acad Sci U S A 102: 331-336.
12. Pfeuty B, Kaneko K (2009) The combination of positive and negative feedback loops confers exquisite flexibility to biochemical switches. Phys Biol 6: 046013.
13. Williams JW, Cui X, Levchenko A, Stevens AM (2008) Robust and sensitive control of a quorum-sensing circuit by two interlocked feedback loops. Mol Syst Biol 4: 234.
14. Taga ME, Bassler BL (2003) Chemical communication among bacteria. Proc Natl Acad Sci U S A 100 Suppl 2: 14549-14554.
15. Urbanowski ML, Lostroh CP, Greenberg EP (2004) Reversible acyl-homoserine lactone binding to purified Vibrio fischeri LuxR protein. J Bacteriol 186: 631-637.
16. Taylor AF, Tinsley MR, Wang F, Huang Z, Showalter K (2009) Dynamical quorum sensing and synchronization in large populations of chemical oscillators. Science 323: 614-617.
17. De Monte S, d'Ovidio F, Dano S, Sorensen PG (2007) Dynamical quorum sensing: Population density encoded in cellular dynamics. Proc Natl Acad Sci U S A 104: 18377-18381.
18. Danino T, Mondragon-Palomino O, Tsimring L, Hasty J (2010) A synchronized quorum of genetic clocks. Nature 463: 326-330.
19. Prindle A, Samayoa P, Razinkov I, Danino T, Tsimring LS, et al. (2012) A sensing array of radically coupled genetic 'biopixels'. Nature 481: 39-44.
20. Gregor T, Fujimoto K, Masaki N, Sawai S (2010) The onset of collective behavior in social amoebae. Science 328: 1021-1025.
21. Kamino K, Fujimoto K, Sawai S (2011) Collective oscillations in developing cells: insights from simple systems. Dev Growth Differ 53: 503-517.
22. Goldbeter A (1996) Biochemical oscillations and cellular rhythms. The molecular bases of periodic and chaotic behaviour.: Cambridge Univ. Press.

23. Keener J, Sneyd J (2008) Mathematical Physiology: Springer-Verla.
24. Alon U (2006) An Introduction to Systems Biology: Design Principles of Biological Circuits: Chapman & Hall.
25. Rue P, Garcia-Ojalvo J (2011) Gene circuit designs for noisy excitable dynamics. Math Biosci 231: 90-97.
26. Volkmer B, Heinemann M (2011) Condition-dependent cell volume and concentration of Escherichia coli to facilitate data conversion for systems biology modeling. PLoS One 6: e23126.
27. Milo R, Jorgensen P, Moran U, Weber G, Springer M (2010) BioNumbers--the database of key numbers in molecular and cell biology. Nucleic Acids Res 38: D750-753.
28. Kaufmann GF, Sartorio R, Lee SH, Rogers CJ, Meijler MM, et al. (2005) Revisiting quorum sensing: Discovery of additional chemical and biological functions for 3-oxo-N-acylhomoserine lactones. Proc Natl Acad Sci U S A 102: 309-314.
29. Pai A, You L (2009) Optimal tuning of bacterial sensing potential. Mol Syst Biol 5: 286.
30. Parsek MR, Val DL, Hanzelka BL, Cronan JE, Jr., Greenberg EP (1999) Acyl homoserine-lactone quorum-sensing signal generation. Proc Natl Acad Sci U S A 96: 4360-4365.
31. Zulianello L, Canard C, Kohler T, Caille D, Lacroix JS, et al. (2006) Rhamnolipids are virulence factors that promote early infiltration of primary human airway epithelia by Pseudomonas aeruginosa. Infect Immun 74: 3134-3147.
32. Glick R, Gilmour C, Tremblay J, Satanower S, Avidan O, et al. (2010) Increase in rhamnolipid synthesis under iron-limiting conditions influences surface motility and biofilm formation in Pseudomonas aeruginosa. J Bacteriol 192: 2973-2980.
33. Chugani SA, Whiteley M, Lee KM, D'Argenio D, Manoil C, et al. (2001) QscR, a modulator of quorum-sensing signal synthesis and virulence in

Pseudomonas aeruginosa. Proc Natl Acad Sci U S A 98: 2752-2757.
34. Pearson JP, Pesci EC, Iglewski BH (1997) Roles of Pseudomonas aeruginosa las and rhl quorum-sensing systems in control of elastase and rhamnolipid biosynthesis genes. J Bacteriol 179: 5756-5767.
35. Ochsner UA, Reiser J (1995) Autoinducer-mediated regulation of rhamnolipid biosurfactant synthesis in Pseudomonas aeruginosa. Proc Natl Acad Sci U S A 92: 6424-6428.
36. van Delden C, Comte R, Bally AM (2001) Stringent response activates quorum sensing and modulates cell density-dependent gene expression in Pseudomonas aeruginosa. J Bacteriol 183: 5376-5384.
37. Seet Q, Zhang LH (2011) Anti-activator QslA defines the quorum sensing threshold and response in Pseudomonas aeruginosa. Mol Microbiol 80: 951-965.
38. Pearson JP, Passador L, Iglewski BH, Greenberg EP (1995) A second N-acylhomoserine lactone signal produced by Pseudomonas aeruginosa. Proc Natl Acad Sci U S A 92: 1490-1494.
39. Ravn L, Christensen AB, Molin S, Givskov M, Gram L (2001) Methods for detecting acylated homoserine lactones produced by Gram-negative bacteria and their application in studies of AHL-production kinetics. J Microbiol Methods 44: 239-251.
40. Welch M, Dutton JM, Glansdorp FG, Thomas GL, Smith DS, et al. (2005) Structure-activity relationships of Erwinia carotovora quorum sensing signaling molecules. Bioorg Med Chem Lett 15: 4235-4238.
41. Welch M, Todd DE, Whitehead NA, McGowan SJ, Bycroft BW, et al. (2000) N-acyl homoserine lactone binding to the CarR receptor determines quorum-sensing specificity in Erwinia. EMBO J 19: 631-641.
42. Kaplan HB, Greenberg EP (1985) Diffusion of autoinducer is involved in regulation of the Vibrio fischeri luminescence system. J Bacteriol 163: 1210-1214.
43. Lupp C, Urbanowski M, Greenberg EP, Ruby EG (2003) The Vibrio fischeri

quorum-sensing systems ain and lux sequentially induce luminescence gene expression and are important for persistence in the squid host. Mol Microbiol 50: 319-331.

44. Perez PD, Weiss JT, Hagen SJ (2011) Noise and crosstalk in two quorum-sensing inputs of Vibrio fischeri. BMC Syst Biol 5: 153.
45. Gerisch G, Wick U (1975) Intracellular oscillations and release of cyclic AMP from Dictyostelium cells. Biochem Biophys Res Commun 65: 364-370.
46. Stewart PS (2003) Diffusion in biofilms. J Bacteriol 185: 1485-1491.
47. Dilanji GE, Langebrake JB, De Leenheer P, Hagen SJ (2012) Quorum activation at a distance: spatiotemporal patterns of gene regulation from diffusion of an autoinducer signal. J Am Chem Soc 134: 5618-5626.
48. Bowen WJ, Martin HL (1964) The Diffusion of Adenosine Triphosphate through Aqueous Solutions. Arch Biochem Biophys 107: 30-36.
49. Dworkin M, Keller KH (1977) Solubility and diffusion coefficient of adenosine 3':5'-monophosphate. J Biol Chem 252: 864-865.
50. Cohen MH, Drage DJ, Robertson A (1975) Iontophoresis of cyclic AMP. Biophys J 15: 753-763.
